# A structural and mechanistic study of π-clamp-mediated cysteine perfluoroarylation

**DOI:** 10.1038/s41598-017-08402-2

**Published:** 2017-08-11

**Authors:** Peng Dai, Jonathan K. Williams, Chi Zhang, Matthew Welborn, James J. Shepherd, Tianyu Zhu, Troy Van Voorhis, Mei Hong, Bradley L. Pentelute

**Affiliations:** Department of Chemistry, Massachusetts Institute of Technology, Cambridge, Massachusetts 02139 United States

## Abstract

Natural enzymes use local environments to tune the reactivity of amino acid side chains. In searching for small peptides with similar properties, we discovered a four-residue π-clamp motif (Phe-Cys-Pro-Phe) for regio- and chemoselective arylation of cysteine in ribosomally produced proteins. Here we report mutational, computational, and structural findings directed toward elucidating the molecular factors that drive π-clamp-mediated arylation. We show the significance of a *trans* conformation prolyl amide bond for the π-clamp reactivity. The π-clamp cysteine arylation reaction enthalpy of activation (ΔH^‡^) is significantly lower than a non-π-clamp cysteine. Solid-state NMR chemical shifts indicate the prolyl amide bond in the π-clamp motif adopts a 1:1 ratio of the *cis* and *trans* conformation, while in the reaction product Pro3 was exclusively in *trans*. In two structural models of the perfluoroarylated product, distinct interactions at 4.7 Å between Phe1 side chain and perfluoroaryl electrophile moiety are observed. Further, solution ^19^F NMR and isothermal titration calorimetry measurements suggest interactions between hydrophobic side chains in a π-clamp mutant and the perfluoroaryl probe. These studies led us to design a π-clamp mutant with an 85-fold rate enhancement. These findings will guide us toward the discovery of small reactive peptides to facilitate abiotic chemistry in water.

## Introduction

Chemo- and regioselective modification of proteins is a chemical challenge because polypeptide chains contain numerous similarly reactive functional groups^1, 2^. Often the polymer, fluorophore, or small-molecule drug is rendered electrophilic for conjugation chemistry at nucleophilic protein side-chains. Many undesired side reactions occur and purification often fails. Major advances to overcome these chemistry challenges have been made, including the use of non-natural clickable amino acids within proteins for bio-orthogonal chemistry^3, 4^, engineered enzymatic systems^5^ such as sortase A^6^, and non-enzymatic routes that leverage selective chemistry between a matched reaction pair^7–11^ such as the reaction of a N-terminal cysteine with a thioester^11^.

**Figure 1:**
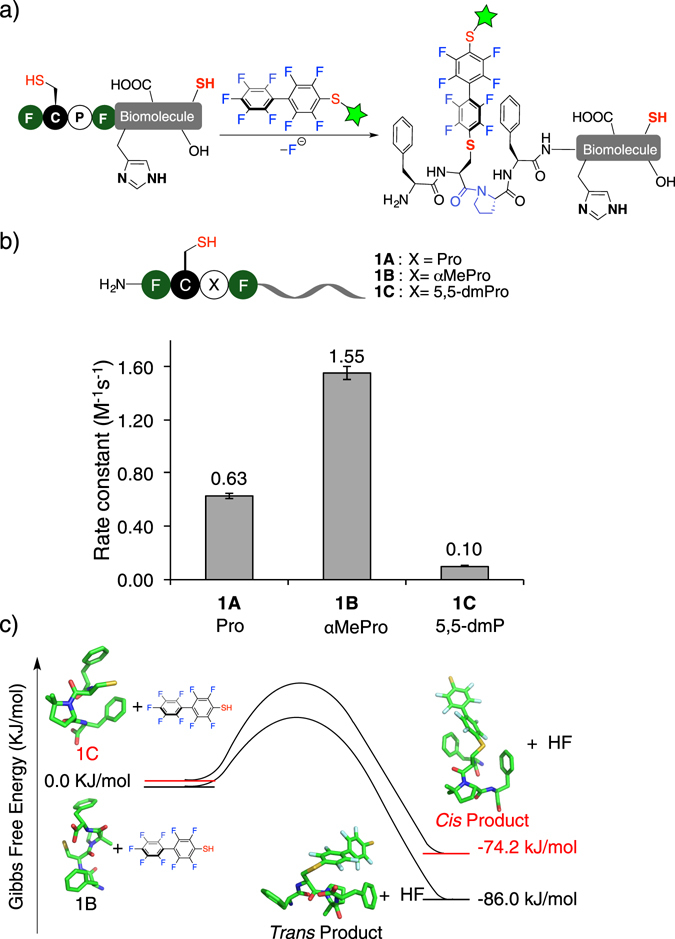
*Trans* conformation of the Cys-Pro amide bond is critical for π-clamp-mediated arylation. (**a**) Scheme of π-clamp-mediated cysteine arylation. (**b**) Reaction rate of π-clamp-mediated arylation is influenced by proline mutation. Peptides **1A**–**1C** were reacted with probe **2**. Reaction conditions: 200 mM phosphate, 20 mM TCEP, pH 8.0, 37 °C. (**c**) Calculated Gibbs free energy changes of the perfluoroarylation reaction involving *trans* Pro π-clamp mutant (**1B**, black) and *cis* Pro π-clamp mutant (**1C**, red). Representative computed geometries of the π-clamp mutants (**1B** and **1 C**) and the perfluoroarylated products used in the DFT calculation are also shown.

Recently we reported an enzyme-free, one-step, regio- and chemoselective protein-modification method based on cysteine arylation^12^. We discovered that the π-clamp tetrapeptide (Phe-Cys-Pro-Phe) possessed unique reactivity to achieve self-labeling with a perfluoroaryl (PFA) electrophile (Fig. 1a). The reaction even proceeded selectively in complex glycosylated IgG proteins. Each residue within the π-clamp is important as mutations often diminished the reaction efficiency. Further, the π-clamp-mediated arylation reaction rate is tunable by over four orders of magnitude with different salts^13^. Ammonium sulfate and other structure-stabilizing salts accelerated the reaction, while the addition of a denaturing salt such as guanidinium chloride impeded the reaction. Taken together, these results prompted us to carry out a systematic investigation of the π-clamp-promoted reaction. Here we employ kinetic studies, solution and solid-state NMR, and density functional theory (DFT) calculations to determine the structure-reactivity relationships that underscore this π-clamp mediated reaction. Our findings suggest several structural and chemical features contribute to the unique reactivity of π-clamp including the *trans* prolyl amide bond, lowered reaction ΔH^‡^, reduced cysteine p*K*_a_ and side chain-perfluoroaryl electrophile interactions.

## Results

### *Trans* prolyl amide accelerates π-clamp-mediated arylation reaction

We first investigated if the prolyl amide conformation being either *cis* or *trans* in the π-clamp was important. Proline is conformationally unique among amino acids as the backbone phi (ϕ) torsion angle is restricted by the side chain ring structure. The Xaa-Pro amide bond can undergo *cis*/*trans* isomerization while other amide bonds are found in the *trans* conformation for most proteins^14, 15^. Pro is thought to be a key player in the π-clamp, as mutation to D-Pro abrogates the reaction rate^12^. To understand if the conformation of the Cys-Pro amide bond perturbs the reaction we used mutation studies. Proline analogues containing certain functional groups can constrain the Cys-Pro conformation; analogues α-methylproline (αMePro)^16, 17^ and 5,5-dimethylproline (5,5-dmP)^18, 19^ strongly promote the *trans* and *cis* conformations, respectively. Three peptides incorporating proline (the π-clamp, peptide **1A**), α-methylproline (peptide **1B**) and 5,5-dimethylproline (peptide **1C**) at residue 3 were synthesized. The rate constants of their reactions with the PFA probe **2** were measured (Figs S1–S3). We found the α-methylproline mutation (**1B**) increased the rate constant by a factor of 2.5 relative to π-clamp **1A**, whereas the 5,5-dimethylproline mutation (**1C**) led to a significant loss of reactivity (Fig. 1b), indicating that *trans* Pro promoted the π-clamp-mediated conjugation.

Computational tools were employed to calculate the Gibbs free energy change (∆G) of π-clamp-mediated arylation reaction, to further study whether a *trans* proline analogue provided any energetic advantage. We previously investigated the reaction energy pathway for *cis* Pro π-clamp^12^. To calculate ∆G of the arylation reaction between peptide **1B** and PFA probe using density functional theory (DFT), we first extracted three snapshots from molecular dynamics (MD) simulations of the *trans* proline π-clamp and then manually added a methyl group to form αMePro. Similarly, for starting structures of peptide **1 C**, we extracted four snapshots from MD simulations of the *cis* proline π-clamp and manually added two methyl groups to form 5,5-dmP. For the product’s starting structure, we manually connected the PFA group to the peptide cysteine. After performing geometry optimizations on the starting structures of the peptide and product (Fig. S4), ∆G of the arylation reaction was calculated using DFT (see Section 3 in supporting information for details). We found that *trans* proline provided a thermodynamic advantage (lower Gibbs free energy of reaction) over *cis* proline by 11.8 kJ/mol for π-clamp-mediated arylation (Fig. 1c and Table S3). Taken together, both mutation and computational studies show a *trans* prolyl amide bond is preferred in the π-clamp-mediated arylation reaction.

### Enthalpy of activation is decreased in π-clamp-mediated arylation

The reactivity of π-clamp cysteine was higher than that of other cysteines, leading to the site-selectivity of π-clamp. Compared to the reaction between PFA probe **2** and the π-clamp double-glycine mutant peptide **3** (GCPG, secondary rate constant *k* = 0.00065 ± 0.00005 M^−1^ s^−1^, Fig. S5), the rate constant for π-clamp peptide **1 A** (0.63 ± 0.02 M^−1^s^−1^) was enhanced 1000-fold. Ammonium sulfate further accelerated π-clamp mediated arylation without affecting the regioselectivity^13^.

We investigated whether and how the π-clamp cysteine arylation reaction activation parameters were different from other cysteine using Eyring equation. We chose to compare π-clamp peptide **1 A**, double glycine mutant **3**, π-clamp proline analogue mutants **1B**–**1 C** and π-clamp mutants with phenylalanine mutated to leucine (**1D**), cyclohexylalanine (**1E**) and pyrenylalanine (**1 F**). PFA probe **2** was reacted with these peptides and the reaction rate constants (*k*) were measured at different temperatures (T) (Table S4, Figs S6–S36) to calculate the enthalpy of activation (ΔH^‡^), the entropy of activation (ΔS^‡^), and the Gibbs energy of activation (ΔG^‡^) (see Methods Section for details). As summarized in Table S5, the ∆H^‡^ for π-clamp and mutants (**1A**–**1 F**) were lowered by 37–84 kJ/mol compared to that of the double glycine mutant **3**, contributing to the significantly enhanced reaction rate. Similarly, to shed light on whether ammonium sulfate changed activation parameters to accelerate the π-clamp-mediated arylation, the rate constant for the reaction between **1 A** and **2** in the presence of two molar (2 M) ammonium sulfate was measured at different temperatures (Figs S38–S42). We found both ΔH^‡^ and ΔS^‡^ were changed by ammonium sulfate (Table 1), but the change of ΔH^‡^ is more significant and is responsible for the reaction acceleration.

**Table 1:** Influence of ammonium sulfate on thermodynamic parameters (310 K) for the arylation reaction between peptide **1 A** and probe **2**.

2 M (NH_4_)_2_SO_4_	ΔH^‡^ (KJ·mol^−1^)	−TΔS^‡^ (KJ·mol^−1^)	ΔG^‡^ (KJ·mol^−1^)	k (M^−1^·s^−1^)
−	89 ± 6	−11 ± 6	78 ± 8	0.63
+	49 ± 3	18 ± 3	67 ± 4	43.3

### π-clamp cysteine p*K*_a_ is reduced even for Phe mutants

We have shown that the cysteine p*K*_a_ of π-clamp is lowered by 0.6 pH unit compared to that of the double glycine mutant^12^. To test whether the cysteine p*K*_a_ values in π-clamp mutants are also perturbed and how important the cysteine p*K*_a_ value is for enhancing its reactivity, we measured the cysteine p*K*_a_ in **1D**, **1E**, **1 F** and **3**.

**Figure 2:**
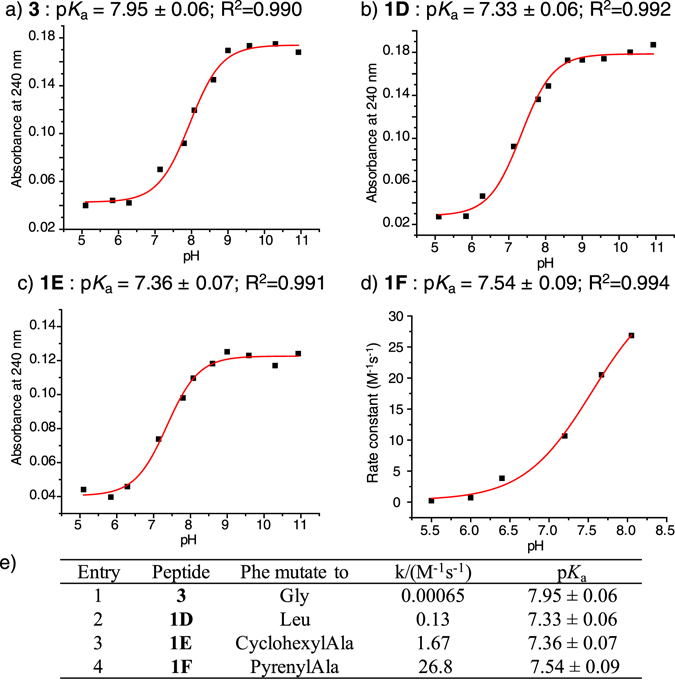
The cysteine p*K*_a_ in π-clamp mutants are reduced. Absorbance at 240 nm across different pHs were measured to determine the cysteine p*K*_a_ in peptide **3** (**a**), **1D** (**b**) and **1E** (**c**). (**d**) Rate constants for the reaction between **1 F** and **2** were measured at different pHs to determine cysteine p*K*_a_ in peptide **1 F**. (**e**) Summary of p*K*_a_ and the reaction rates of π-clamp mutants.

Several methods have been developed to measure the cysteine p*K*_a_ in biomolecules. Spectrophotometric titration^20–22^, where UV absorbance of the cysteine containing molecule is plotted against different pH values, is a convenient method. This method is based on the fact that ionization of cysteine thiol to thiolate results in a large increase in its molar absorption coefficient at 240 nm^20^. To calculate the cysteine p*K*_a_ of **1D**, **1E** and **3**, we measured the absorbance at 240 nm (A240) of the peptides dissolved in buffers at different pHs. The absorbance of the buffer was used as a blank. Then the p*K*_a_ was determined (Fig. 2a–c) by fitting the UV absorbance and pH values with the following equation:

$$y=\frac{A\times {10}^{pH}+B\times {10}^{p{K}_{a}}}{{10}^{p{K}_{a}}+{10}^{pH}}$$

where y is the absorbance of the peptide at 240 nm, A is the upper plateau of absorbance at high pH, and B is the lower plateau at low pH. The cysteine p*K*_a_ in the double glycine mutant **3** was measured to be 7.95, while the cysteine p*K*_a_ of both **1D** and **1E** were 0.6 pH unit lower than that of **3**.

The A240 method could not be applied to **1 F** because of the strong absorbance of the pyrenyl group at 240 nm. Instead we measured p*K*_a_ based on the reactivity of cysteine at different pHs. The p*K*_a_ values of cysteine in proteins have been measured by determining their reaction rates with a reagent that is more reactive with thiolate than thiol^22, 23^. Here we used the rate constant of the S_N_Ar reaction between **1 F** and **2** to fit for p*K*_a_, since thiolate is more reactive than thiol in the perfluoroarylation reaction. The rate constants were plotted against pH and fit with the same equation as in the A240 method, where y is the reaction rate constant, and A and B are the upper and lower plateaus for the reaction rates (Fig. 2d). The cysteine p*K*_a_ of **1 F** was determined to be 0.4 pH unit lower than **3**. In Fig. 2e, we summarize the cysteine p*K*_a_ values and the rate constants for the peptides studied. We observed a more than 10-fold rate constant difference between **1D** and **1E**, even though their p*K*_a_ values were similar. In addition, peptide **1 F** was more reactive than peptides **1D** and **1E**, but the cysteine p*K*_a_ of **1 F** was higher. Although all three π-clamp mutants had a lower cysteine pK_a_ compared to that of the double glycine mutant **3** which facilitated ionization of the thiol to the more reactive thiolate, this p*K*_a_ effect was not the only factor to account for the enhanced reactivity of the π-clamp.

### ssNMR indicates π-clamp adopts 1:1 *cis*/*trans* Pro3 while the perfluoroarylated product adopts only *trans*

**Figure 3:**
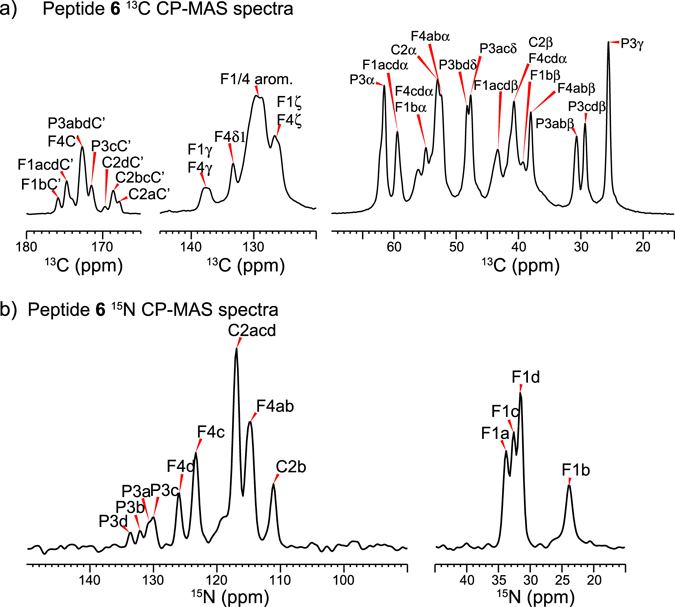
^13^C (**a**) and ^15^N (**b**) cross-polarization magic-angle spinning (CP-MAS) spectra of the PFA-tagged π-clamp peptide **6** show four sets (denoted as (**a**,**b**,**c**,**d**) after the residue number) of well resolved peaks, indicating conformational polymorphism.

To characterize the backbone conformation of the π-clamp and the S-perfluoroarylated product (PFA-tagged π-clamp), 1D and 2D ^13^C magic-angle-spinning (MAS) NMR spectra were measured on π-clamp peptide **5** (Fig. S43) and π-clamp PFA-tagged product **6** (Fig. S44), in which the π-clamp motif Phe-Cys-Pro-Phe were uniformly ^13^C and ^15^N labeled. The ^13^C cross-polarization (CP) spectrum of peptide **6** (Fig. 3a) shows sharp resonances, but with more peaks than expected for four labeled residues, indicating the presence of multiple resolvable peptide conformations. The Pro3 conformational heterogeneity is manifested as two resolved Cβ peaks around 30 ppm, and two partially resolved Cδ peaks around 48 ppm. In the carbonyl region, structural polymorphism is also readily observed, especially among the multiple Cys2 CO peaks, ~169 ppm, which are resolved from the carbonyl signals of the other labeled residues. The ^15^N CP spectrum (Fig. 3b) further highlights this conformational polymorphism: the N-terminal Phe1 amino group shows four resolved ^15^N signals at 22–35 ppm, indicating four distinct conformations. Likewise, Pro3 exhibits four resolved ^15^N peaks at 130–135 ppm.

**Figure 4:**
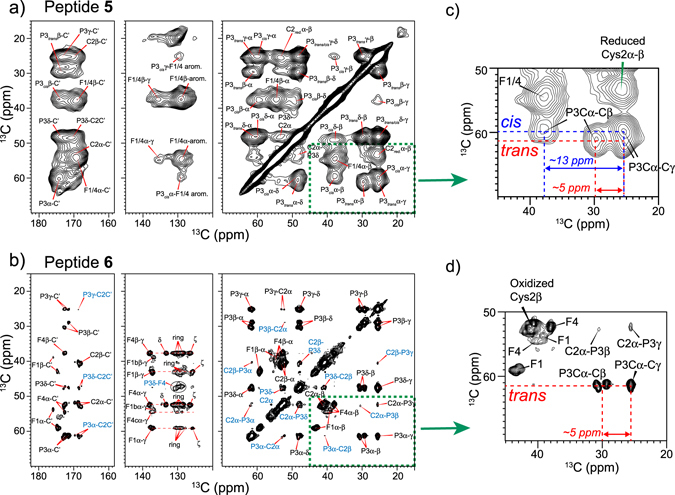
2D ^13^C-^13^C correlation spectra of the (**a**) unreacted π-clamp peptide **5** and (**b**) PFA-tagged peptide **6**. The spectra were acquired at 298 K. (**c**,**d**) Enlarged regions showing Pro3 and Cys2 cross peaks. The Cys2 Cβ chemical shift indicates that Cys2 is in a reduced state (~25 ppm) in peptide **5** (**c**), and in an oxidized state (~40 ppm) in the PFA-tagged peptide **6** (**d**). The chemical shift difference between Pro Cβ and Cγ indicates the isomer state. The Pro intensity in (**c**) indicates an about 1:1 ratio between the *cis* and *trans* isomers in the unreacted peptide **5**. The Pro signal in (**d**) shows only the *trans* Pro conformation is adopted in PFA-tagged peptide **6**.

To further resolve and assign the ^13^C and ^15^N chemical shifts, we measured 2D ^13^C-^13^C correlation spectra (Fig. 4) of peptides **5** and **6**, and 2D ^15^N-^13^C correlation spectra (Fig. S45) of peptide **6**. The 2D ^13^C-^13^C correlation spectrum of peptide **5** (Fig. 4a) shows larger ^13^C line widths than the PFA-tagged peptide **6** (Fig. 4b) due to the conformational distribution and the dry state of the peptide. Measurement of the dried, unreacted peptide **5** by solid-state NMR was necessary due to the unfavorable dynamics of this very soluble peptide in a hydrated state. The aliphatic linewidths of peptide **5** range from 2.0 ppm to 3.0 ppm, while the PFA-tagged peptide **6** exhibits ^13^C linewidths of 0.7–1.4 ppm. Within this linewidth range, peptide **5** shows a single set of chemical shifts for each residue, with the exception of Pro3, which exhibits chemical shifts for both *cis* and *trans* isomers (*vide infra*). The Phe1 and Phe4 chemical shifts are not well resolved: their Cα-Cβ cross peaks partially overlap, and a single set of Cβ cross peaks with the aromatic side chain carbons are observed (Fig. 4a), indicating that the two Phe residues have similar conformations. Cys2 displays a Cβ chemical shift of ~25 ppm (Fig. 4a and c), which corresponds to the reduced state, confirming that the unreacted peptide **5** does not form disulfide bonds.

Upon reaction of peptide **5** with the PFA probe to form product **6**, all ^13^C and ^15^N peaks sharpen (Figs 4b,d and S45), allowing us to resolve four sets of chemical shifts for each residue. In peptide **6**, the inter-residue Cys2-Pro3 cross peaks allow the multiple sets of chemical shifts to be separated into four different molecules. No cross peaks between the different forms are observed at a ^13^C spin diffusion mixing time of 50 ms. Phe1 and Phe4 exhibit resolved Cα and Cβ chemical shifts that differ by ~5 ppm and ~3 ppm respectively (Fig. 4b). Multiple sets of aromatic Cγ, Cδ, Cε and Cζ chemical shifts are also resolved for Phe1 and Phe4, indicating that Phe1 and Phe4 experience different chemical environments, likely due to the PFA tag. Cys2 shows a Cβ chemical shift of ~40 ppm, characteristic of oxidized Cys2, as expected for PFA-modified cysteine.

We were especially interested in the prolyl amide bond conformation. Pro3 shows interesting chemical shift differences between the peptide **5** and the PFA-tagged peptide **6**. In the unreacted state (peptide **5**), two sets of Pro3 Cβ and Cγ chemical shifts are observed: one set shows a large Cβ and Cγ chemical shift difference of ~13 ppm while the other set shows a smaller chemical shift difference of ~5 ppm (Fig. 4c). These Cβ and Cγ chemical shift differences^24^ are characteristic of the *cis* and *trans* isomers of Pro, and peak intensities indicate that the two forms have a *cis*: *trans* ratio of 1:1 in the unreacted peptide **5**. Upon reaction with the PFA probe, the multiple resolved Pro3 chemical shifts all show similar Cβ and Cγ chemical shift differences of ~5 ppm, indicating that the Pro3 in peptide **6** predominantly shifts to the *trans* conformer (Fig. 4d).

Overall, these ^13^C and ^15^N chemical shifts (Tables S6, S7) indicate there is no single well-defined secondary structure for the π-clamp motif in peptide **5** or **6**. The unreacted π-clamp peptide **5** adopts a broadly distributed conformation with an approximate 1:1 ratio of the *cis* and *trans* Pro3 isomer. Reacting with the PFA probe causes peptide **6** to adopt four distinct conformations, but only the *trans* isomer at Pro3. Using the compiled ^13^C and ^15^N chemical shifts of peptide **6**, a TALOSN^25^ prediction yielded similar backbone (ϕ,ψ) torsion angles for the four forms (Fig. S46), with the largest torsion angle difference occurring at Phe4. This difference is likely from prediction uncertainty due to the lack of chemical shift information of the residue C-terminal to Phe4. For the unreacted peptide **5**, because of the large spectra linewidths, there is significant uncertainty in predicting these (ϕ,ψ) torsion angles. However, since all four distinct sets of peptide **6** chemical shifts fall within the broad linewidths of the unreacted peptide **5**, the average backbone conformations may not differ dramatically between the two peptides.

### A clamp-like claw structure is one of the two reaction products from ssNMR and modeling studies

**Figure 5:**
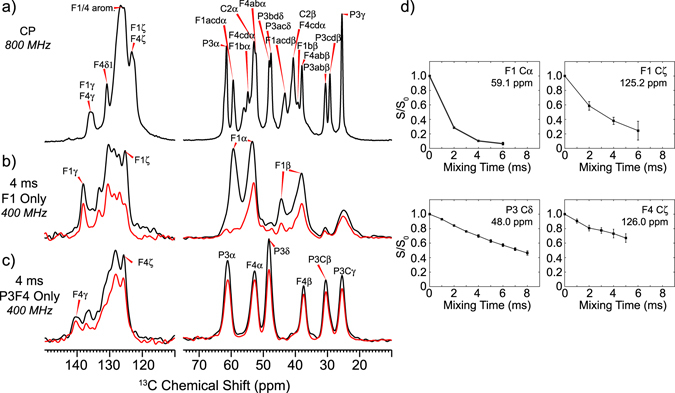
^13^C-^19^F distances are measured using ^19^F-dephased and ^13^C-detected REDOR spectra of the PFA-tagged π-clamp peptides. Representative spectra were shown. (**a**) ^13^C CP-MAS spectra of the fully labeled peptide **6**, showing the aliphatic (right) and aromatic (left) region. Full intensity (S_0_, black) and dephased (S, red) REDOR spectra at a REDOR mixing time of 4 ms for the F1-only labeled π-clamp peptide **7** (**b**) and P3F4-only labeled peptide **8** (**c**) are shown. (**d**) Experimental S/S_0_ dephasing values for four representative ^13^C resonances.

To generate a structural model for the perfluoroarylated π-clamp product based on the backbone conformation predicted from chemical shifts, ^13^C-^19^F distances were measured using ^13^C-^19^F REDOR of two partially labeled π-clamp peptides: peptide **7** with uniform ^13^C, ^15^N labeling at only Phe1 (Fig. S47) and peptide **8** with uniform ^13^C, ^15^N labeling at Pro3 and Phe4 (Fig. S48). By examining these two peptides, we can determine the distance between the PFA and each Phe. Figure 5 shows representative REDOR S_0_ and S spectra at a mixing time of 4 ms. ^13^C spins that are close to the ^19^F spins exhibit larger intensity differences between the S_0_ and S spectra than ^13^C spins that are farther from ^19^F spins. Figure 5d plots the intensity ratio S/S_0_ as a function of the REDOR mixing time for a few representative peaks; the full set of REDOR curves used for modelling are summarized in Fig. S49. The Phe1 backbone Cα peak has the largest dephasing among all peaks, reaching a value of 0.07 ± 0.02 at 6 ms, while the Phe1 Cζ shows a more moderate dephasing trend. The Pro3 Cδ and Phe4 Cζ show linear decay in contrast to the expected universal two-spin REDOR curve. We attribute these decay trajectories to the multi-spin nature of the system, with 8 ^19^F spins at varying distances from each ^13^C.

Given the system complexity, we first analyzed these ^13^C-^19^F REDOR data semi-quantitatively. In contrast with the Phe1 backbone Cα peak which has the largest degree of dephasing, the Phe4 Cα and Cβ backbone sites show minimal dephasing, reaching only S/S_0_ values of ~0.8 at 8 ms. This indicates that the PFA tag is closer to Phe1 than Phe4. Among the ^13^C signals from Pro3, Cδ reaches the lowest dephasing at a S/S_0_ value of ~0.4 at 8 ms, while Pro3 Cα, Cβ and Cγ each show minimum S/S_0_ of ~0.6 at 8 ms, indicating Pro3 Cδ is closer to the PFA tag.

**Figure 6:**
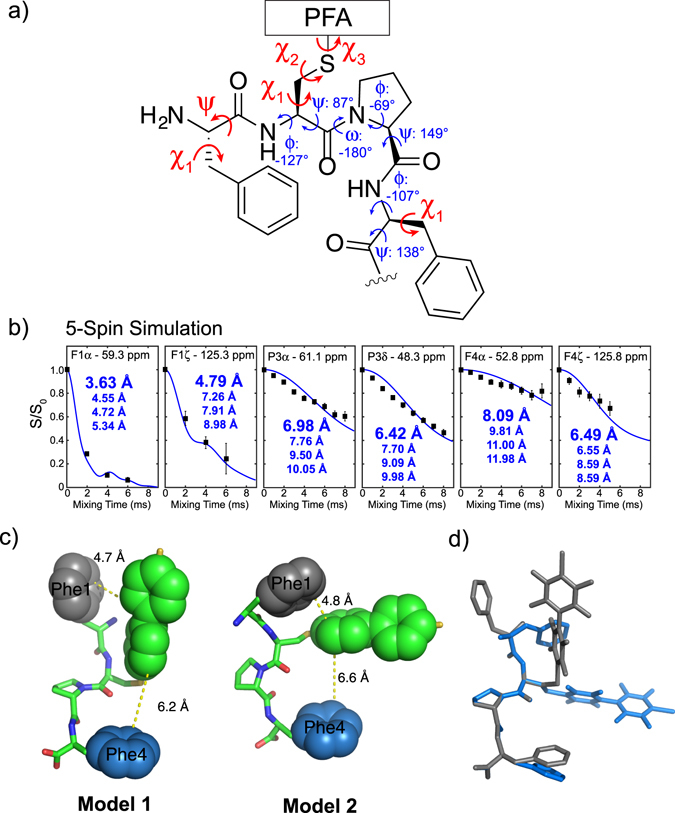
Two structural models for PFA-tagged π-clamp show similar backbone structures but distinct Phe1-PFA interaction. (**a**) The average predicted backbone torsion angles (shown in blue) and the torsion angles varied for fitting (shown in red). (**b**) Representative model-dependent, best-fit 5-spin SIMPSON simulations of the experimental REDOR curves for the resolved ^13^C sites. For each simulation of torsion angle combinations, the four nearest ^19^F atoms to a ^13^C site were included in the simulation. (**c**) Structural model of the PFA-tagged π-clamp motif based on the predicted backbone torsion angles and REDOR distance restraints. Model 1 (left) uses Phe1 Cα and Cβ for fitting, giving torsion angles of Phe1 ψ = 90° and χ_1_ = −60°, Cys2 χ_1_ = 60°, χ_2_ = −60°, and χ_3_ = −30°, and Phe4 χ_1_ = −90°. Model 2 (right) uses Phe1 Cα’ and Cβ’ for fitting, giving torsion angles of Phe1 ψ = −60° and χ_1_ = 150°, Cys2 χ_1_ = −120°, χ_2_ = 180°, and χ_3_ = 180°, and Phe4 χ_1_ = −120°. The shortest carbon-to-carbon distance between Phe side chains and PFA were shown. (**d**) Superimposition of the two models around Pro3.

More quantitative distance information was extracted by multi-spin simulations utilizing the SIMPSON program^26^. We compared the experimental data first to 2-spin simulations to obtain the apparent ^13^C-^19^F distance, and then to 5-spin system containing one ^13^C and four ^19^F spins in model-dependent simulations. In these simulations, the ψ and χ_1_ angle of Phe1, χ_1_, χ_2_ and χ_3_ of Cys2 and χ_1_ of Phe4 were iteratively set to 180°, 60° or −60°. For each simulated curve, the root-mean-square deviation (RMSD) was calculated between each experimental REDOR data set, and the minimum among all calculated RMSD values corresponds to the best fit. The 2-spin simulations provided reasonable fits for each ^13^C site (Fig. S50). For all sites in Pro3, the distances range from 6.3–7.0 Å. Slightly longer distances for Phe4 Cα and Cβ (7.7 Å and 7.4 Å respectively) are observed, and the distance for Phe4 Cζ is 6.0 Å. For Phe1, the fast dephasing Cα and Cζ fit relatively well to distances of 3.8 Å and 5.2 Å, respectively. The 5-spin model-dependent simulations unfortunately did not simultaneously fit well for all ^13^C sites with any set of dihedral angle combinations. However, taking the best-fit 5-spin REDOR curve for each ^13^C site (Figs 6b, S51), the shortest ^13^C-^19^F distance among the four nearest neighbor ^19^F agreed well with the best-fit distances determined from the 2-spin simulations.

These distances were taken as a set of REDOR derived, nearest-neighbor ^13^C-^19^F distances: 3.6 Å F1α, 4.6 Å F1β, 6.0 Å F1α’, 6.0 Å F1β’, 4.8 Å F1ζ, 7.0 Å P3α, 7.5 Å P3β, 7.7 Å P3γ, 6.4 Å P3δ, 8.1 Å F4α, 7.8 Å F4β, and 6.5 Å F4ζ. Using a smaller interval of 30°, the dihedral angles were rotated iteratively again and the nearest neighbor ^13^C-^19^F distance was compared to the above set of distances. In the generated best-fit models, shown in Fig. 6c, model 1 (left) uses F1 Cα and Cβ, and model 2 (right, wide clamp-like) uses F1 Cα’ and Cβ’ for fitting. The ^13^C-^19^F distances from these models were used in 5-spin REDOR simulations, and plotted against the experimental REDOR S/S_0_ values (Figs S52, S53) for validation. Model 2 gave decent fits for all ^13^C sites while model 1 has worse fits, even though the shortest ^13^C-^19^F distances are similar in the two models. In both models, distinct non-covalent interactions between Phe1 side chain and perfluoroaryl electrophile at around 4.7 Å distance and similar backbone structures were observed (Fig. 6c and d).

### A pyrenylalanine π-clamp mutant non-covalently interacts with the perfluoroaryl electrophile in solution

**Figure 7:**
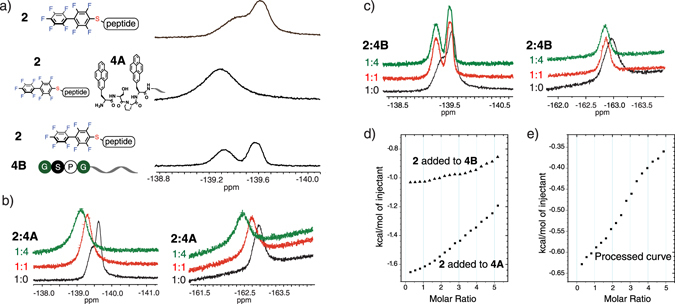
F NMR and ITC indicate interactions between peptide **4 A** side chain and PFA probe **2**. (**a**) ^19^F solution NMR spectra for probe **2** (top), **2** mixed with 1 equivalent **4 A** (middle), and **2** mixed with 1 equivalent **4B** (bottom). Conditions: 2 mM peptide or probe, 20 mM phosphate, pH 7, 10% D_2_O, 90% H_2_O. (**b**,**c**) ^19^F solution NMR spectra for the titration experiment in which the equivalent of peptide **4 A** (**b**) or **4B** (**c**) relative to **2** was increased. Concentration of **2** was fixed at 2 mM. (**d**) Isothermal titration calorimetry binding curves for **2** interacting with **4 A** and **4B**. 5 mM probe **2** was added into 0.2 mM **4 A** or **4B** in the titration experiment. **e**) Processed ITC binding curve where the interaction between **2** and **4B** was deducted as background from the interaction between **2** and **4 A**.

We hypothesized the phenyalanine side chains in π-clamp interact non-covalently with the PFA electrophile to facilitate the reaction. ^19^F solution NMR and isothermal titration calorimetry experiments were carried out to test this hypothesis. We started with an un-reactive π-clamp mutant (Phe-Ser-Pro-Phe), but were unable to detect interactions by NMR experiments. Prompted by our earlier experiments, we investigated a pyrenylalanine mutant which may interact with the PFA electrophile more strongly. Peptide **4 A** (Py-Ser-Pro-Py, where Py is pyrenylalanine) and the control peptide double-glycine mutant **4B** (Gly-Ser-Pro-Gly) were prepared. We mixed **4 A** with 1 equivalent of probe **2** and observed a change in ^19^F solution NMR chemical shift and peak shape when compared to the mixture of **4B** and probe **2**, or probe **2** alone (Fig. 7a). In addition, the ^19^F chemical shift was changed during titration when we added **4 A** to a fixed amount of **2** (Fig. 7b). In contrast, this effect was not observed for the same titration of peptide **4B** (Fig. 7c). These results suggest the pyrenyl side chains are interacting with the PFA electrophile. To corroborate the ^19^F solution NMR studies, we investigated the interaction of **4 A** and **2** by isothermal titration calorimetry. Probe **2** was added to peptide **4 A** or **4B** to detect non-covalent interaction (Fig. 7d). When compared to the non-pyrenylalanine case **4B** as a control, weak binding was observed between **4 A** and **2**. The binding curve (Fig. 7e) did not display simple two state interactions and could not be fit nor could we determine the binding stoichiometry.

## Discussion

The π-clamp accelerates abiotic perfluoroarylation chemistry in water. Here we carried out structural and mechanistic studies to understand this transformation. Several structural and chemical features were found to facilitate the reaction. We found that Pro3 played an important role in the π-clamp function. A π-clamp α-methylproline mutant favoring the *trans* Pro conformation accelerated the rate of arylation by more than 15-fold, compared to the 5,5-dimethylproline mutant that favored a *cis* Pro conformation. *Trans* Pro provided a thermodynamic advantage over *cis* Pro for π-clamp-mediated arylation as shown by DFT calculation. The ΔH^‡^ for the π-clamp arylation reaction is decreased by 64 kJ/mol when compared to a non-π-clamp cysteine, enabling 1000-fold rate enhancement and regioselective cysteine modification at the π-clamp. Ammonium sulfate salt lowers the π-clamp arylation reaction ΔH^‡^ by 40 kJ/mol relative to the reaction without the salt and thus promote the reaction. In addition, the π-clamp cysteine p*K*_a_ is reduced by 0.4–0.6 pH unit even for Phe mutants.

Structural and biophysical studies indicate certain conformations may promote the reaction, and side-chains in the π-clamp interact non-covalently with the PFA electrophile. Solid-state NMR indicated a mixture of *cis*/*trans* Pro in the unreacted π-clamp was fully converted to the *trans* conformation upon perfluoroarylation. Two ssNMR structural models of the reaction product highlight the interactions between the Phe1 side chain and perfluoroaryl electrophile moiety. ^19^F solution NMR titration experiments and the ITC data support the hypothesis that the π-clamp side chains may interact with the PFA electrophile.

The structural models from our ssNMR study are not fully consistent with the π-clamp α-methylproline mutant product structures used in DFT calculations, although they both have *trans* Pro. As the comparison shown in Fig. S54, the largest torsion angles differences between the calculated structures and clamp-like NMR structure model 2 occurred at Phe1-psi and Pro3-psi. These two angles contribute to the calculated structures being more compact than the NMR structure. In addition, all of the calculated structures have Phe4 in close proximity to the PFA tag. This would theoretically cause the Phe4 Cα and Cβ simulated REDOR curves to decay rapidly, while the experimental data shows a much slower decay (Fig. S54). The inconsistency may come from the fact that we did not directly sample the PFA-labeled π-clamp product in MD simulations, due to a lack of parameters to describe the PFA in the reaction product. In addition, two slightly different molecules have been used in our computation and ssNMR studies. We think that the ssNMR models better describe the structural feature of the PFA labeled π-clamp product. However, our DFT calculations which indicate that *trans* Pro is thermodynamically favored over *cis* proline in the π-clamp perfluoroarylation reaction should be valid, considering that for all the geometrically-optimized calculated structures we sampled, larger or similar reaction ∆G were observed for *trans* Pro compared to the *cis* Pro structures.

Our studies here have led to the discovery of a π-clamp mutant **1 G** with an 85-fold rate enhancement. A convergent effect was observed when we combined α-methylproline, which promoted the *trans* Pro conformation, and the large hydrophobic side chain in pyrenylalanine. Taken together, we generated π-clamp mutant **1 G** with a rate constant of 53.3 ± 2.3 M^−1^ s^−1^, which is 85-fold higher π-clamp peptide **1 A** (Figs S55, S56). We anticipate our findings could direct us toward the design and screening of other small reactive peptides to facilitate selective abiotic chemistry enabled by local environment.

## Methods

### Kinetics Study

The reactions were carried out with 200 mM phosphate, 20 mM TCEP at 37 °C unless otherwise noted. To measure the second order rate constants, reaction mixture was prepared on ice and divided into several 10-µL aliquots. All aliquots were immediately put in 37 °C water bath unless otherwise noted. For reactions that takes more than 1 hour to monitor, all aliquots were heated in a PCR machine set at 37 °C to prevent solvent evaporation. Reactions were quenched by addition of 100 µL 50% water: 50% acetonitrile: 0.5% TFA at different time points and then subjected to LC-MS analysis. The initial concentration of probe and substrate were known. The second-order rate constants were determined by fitting the following kinetics equation:

$$y=\frac{ln\frac{{[peptide]}_{0}{[probe]}_{t}}{{[peptide]}_{t}{[probe]}_{0}}}{{[probe]}_{0}-{[peptide]}_{0}}={k}_{2}t$$

Error of reaction rate constant was obtained from the linear fitting of the kinetics curves for measuring the reaction rate constants.

### Determination of the standard enthalpy/entropy of activation

The secondary rate constant (*k*) for the reaction between π-clamp peptide and probe **2** was experimentally measured at different temperatures (T). Then ln(*k*/T) was plotted against 1/T. The standard enthalpy of activation (ΔH^‡^) and the standard entropy of activation (ΔS^‡^) were calculated (Table S5) by fitting ln(*k*/T) against 1/T with the following Eyring equation:

$$ln\frac{k}{T}=ln\frac{\kappa {k}_{B}}{h}+\frac{{\rm{\Delta }}{S}^{\ddagger}}{R}+\frac{-{\rm{\Delta }}{H}^{\ddagger}}{R}\frac{1}{T}$$

where κ is transmission coefficient (κ = 1), *k*_*B*_ is Boltzmann constant, *h* is Planck’s constant, T is absolute temperature and R is gas constant. The errors for ΔH^‡^ and ΔS^‡^ were obtained from the linear fitting. ∆G^‡^ was calculated as ΔH^‡^−TΔS^‡^, and the error for ∆G^‡^ was calculated from error propagation. The Eyring plots were summarized in Fig. S37.

## Electronic supplementary material

Supplementary Information
