# Supplementary material for: A structural and mechanistic study of π-clamp-mediated cysteine perfluoroarylation
Source: Sci Rep. 2017 Aug 11;7:7954. doi: 10.1038/s41598-017-08402-2 (PMC5554146; doi:10.1038/s41598-017-08402-2)
Supplement: Supplementary file 1 — Supplementary Information [file 41598_2017_8402_MOESM1_ESM.pdf]

A structural and mechanistic study of π-clamp-mediated cysteine perfluoroarylation

Peng Dai, Jonathan K. Williams, Chi Zhang, Matthew Welborn, James J. Shepherd, Tianyu Zhu, Troy Van Voorhis, Mei Hong and Bradley L. Pentelute

Department of Chemistry, Massachusetts Institute of Technology,

Cambridge, Massachusetts 02139, United States

Supporting Information

Table of Contents

1. General Considerations 2
   a. Chemicals 2
   b. Solid phase peptide synthesis 2
   c. Peptide purification 3
   d. LC-MS analysis 3
   e. Reaction yield determination 3
   f. Solution NMR 4
   g. Isothermal titration calorimetry (ITC) 4

2. Preparation of S-Perfluoroaryl Modified Electrophile 2 and 2' 4

3. Kinetics Study 5

4. Determination of the standard enthalpy/entropy of activation 5

5. Computation Studies 5

6. Cysteine pK_a measurement 6

7. Solid State NMR Studies 7

Reference 9

Supplementary Tables and Figures 10

1. General Considerations

a. Chemicals

Decafluorobiphenyl was purchased from Oakwood Chemicals (West Columbia, SC). *Tris*(2-carboxyethyl)phosphine hydrochloride (TCEP•HCl) was purchased from Hampton Research (Aliso Viejo, CA). 1-[Bis(dimethylamino)methylene]-1H-1,2,3-triazolo[4,5-b] pyridinium 3-oxid hexafluorophosphate) (HATU), Fmoc protected amino acids were purchased from Chem-Impex International (Wood Dale, IL) unless otherwise noted. Fmoc-αMethylProline-OH was purchased from ChemPep Inc. (Wellington, FL). Fmoc U-13C, 15N-Phe-OH, Fmoc U-13C, 15N-Pro-OH and Fmoc U-13C, 15N-Cys(Trt)-OH were purchased from Cambridge Isotope Laboratories Inc. (Andover, MA). H-Rink Amide Chemmatrix resin was obtained from PCSS BioMatrix Inc. (Quebec, Canada). *N,N*-Dimethylformamide (DMF), dichloromethane (DCM), diethyl ether, HPLC-grade acetonitrile, and guanidine hydrochloride were obtained from VWR International (Philadelphia, PA). All other reagents were purchased from Sigma-Aldrich and used as received unless otherwise noted.

b. Solid phase peptide synthesis

<u>General Protocols</u>

Peptides were synthesized at 0.05 mmol scale on H-Rink Amide Chemmatrix resin. The resin was firstly swelled in dimethylformamide (DMF). General procedure for amino-acid residue coupling: 0.4 mmol Fmoc protected amino acids was dissolved in 1 mL 0.38 M HATU solution in DMF, 200 μL of diisopropylethylamine (DIEA) was added (for coupling of cysteine, 80 μL of DIEA was used to prevent racemization). The mixture was added to resin and react at room temperature for 20 minutes, followed by 3 times wash with DMF, 2 times 8 min deprotection with 20% (v/v) piperidine in DMF and 5 times wash with DMF. The resin was washed thoroughly with DCM and air dried after completion of the stepwise SPPS. The peptide is then simultaneously cleaved from the resin and side-chain deprotected by treatment with 2.5% (v/v) water, 2.5% (v/v) 1,2-ethanedithiol (EDT), and 1% (v/v) triisoproprylsilane in neat trifluoroacetic acid (TFA) for 2 hours at room temperature. Resulting solution containing peptide was evaporated by blowing a stream of nitrogen gas over its surface for 15 minutes, then triturated and washed with cold diethyl ether three times. Obtained gummy-like solid was dissolved in 50% H_2O: 50% acetonitrile containing 0.1% TFA and lyophilized.

<u>Special Case</u>

Peptide **1C-Pro**. The methyl groups on the δ carbon of 5,5 dimethylproline hampered formation of the X-dmP peptide bond.[1] So Fmoc-Cys (S-StBu) was used instead of Fmoc-Cys(Trt) to reduce steric

hindrance derived from the bulky trityl group. Coupling of cysteine was carried out as the following "double coupling" conditions. 0.4 mmol Fmoc-Cys(S-StBu) was mixed with 1 mL 0.4 M HATU, 200 μL of diisopropyl ethyl amine (DIEA) and added to resin. After reaction for 3 hours, the resin was washed twice with DMF and coupled with Fmoc-Cys(S-StBu) under the same condition for another 15 hours. Peptide **1C-Pro** was then reduced by TCEP and purified by HPLC to get peptide **1C**.

c. Peptide purification

The same solvent compositions were used in most experiments and will be referred to as A: 0.1% TFA in H_2O and B: 0.1% TFA in acetonitrile. The crude peptide was dissolved in 95% A: 5% B with 6 M guanidinium hydrochloride and purified by semi-preparative RP-HPLC (Agilent Zorbax SB C_{18} column: 21.2 x 250 mm, 7 μm, linear gradient: 5-50% B over 90 min, flow rate: 5 mL/min). 1 μL of each HPLC fraction was mixed with 1 μL of alpha-cyano-4-hydroxycinnamic acid (CHCA) matrix in 75% A: 25% B, spotted with MALDI, and checked for fractions with desired molecular mass. The purity of fractions was confirmed by analytical RP-HPLC (Agilent Zorbax SB C_3 column: 2.1 x 150 mm, 5 μm, gradient: 0-2 minutes 5% B, 2-11 minutes 5-65% B, 11-12 minutes 65% B, flow rate: 0.8 mL/min). HPLC fractions containing only product material were confirmed by LC-MS analysis, combined, and then lyophilized. Peptides synthesized by SPPS and purified by RP-HPLC are listed in Table S1.

d. LC-MS analysis

LC-MS chromatograms and associated mass spectra were acquired using Agilent 6520 ESI-Q-TOF mass spectrometer. Zorbax SB C_3 column: 2.1 x 150 mm, 5 μm was used with column temperature set at 40 °C. MS conditions: positive electrospray ionization (ESI) extended dynamic mode in mass range 300 – 3000 *m/z*, temperature of drying gas = 350 °C, flow rate of drying gas = 11 L/min, pressure of nebulizer gas = 60 psi, the capillary, fragmentor, and octupole rf voltages were set at 4000, 175, and 750, respectively. All chromatograms shown in supplementary figures are total ion currents (TIC); all insets are mass spectrums taken at the highest points of corresponding TIC peaks.

e. Reaction yield determination

Yields for peptide substrates were determined by integrating total ion current (TIC) spectra. First, using Agilent MassHunter software package, the peak area for all relevant peptidic species on the chromatogram were integrated. In cases where no side product was generated in the experiments, the conversion of the limiting reagent equals to the yield of the product. Conversion was calculated by integrating the total ion current (TIC) of the same limiting peptide species within the dynamic linear

range of the LC-MS instrument. Then the yield was calculated as following: %yield = %conversion = 1 - S_t/S_0 where S_t is the peak area of the limiting reagent at time t, and S_0 is the peak area of the limiting reagent at time 0.

f. Solution NMR

^{1}H NMR spectra were recorded on a Bruker Avance 400 MHz spectrometer and are reported in ppm using solvent as an internal standard (DMSO-d6 at 2.50 ppm). ^{19}F NMR spectra were recorded on a Bruker Avance 400 MHz (376 MHz) spectrometer and are reported in ppm with trifluoroacetic acid as an internal standard (at -76.6 ppm). ^{1}H NMR and ^{19}F NMR data are reported as (s = singlet, d = doublet, dd = doublet of doublets, t = triplet, q = quartet, m = multiplet, br = broad; coupling constant(s) in Hz; integration).

g. Isothermal titration calorimetry (ITC)

ITC experiments were carried out using Microcal Isothermal Titration Calorimeter. Peptide 4A and 4B were prepared as solutions containing 0.2 mM peptide, 20 mM phosphate, pH 7.6. Probe 2 was prepared as solution containing 5 mM probe, 20 mM phosphate, pH 7.6. The solutions were used for titration in ITC experiments. Data were processed using MicroCal Analysis software.

2. Preparation of S-Perfluoroaryl Modified Electrophile 2 and 2'

a. Probe **2** was prepared from peptide **2-Cys** as reported previously.[2]

b. Preparation of Probe **2'**

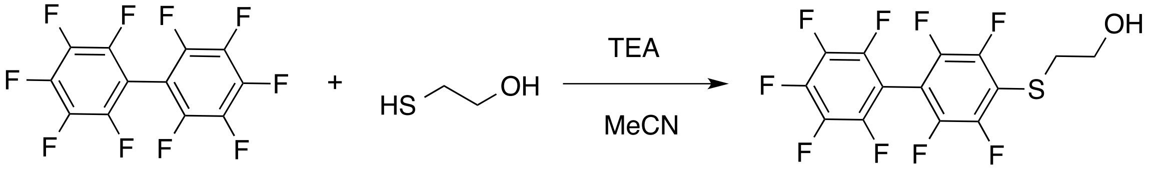

β-mercaptoethanol (39 mg, 0.5 mmol) was added to a solution of decafluorobiphenyl (4.2g, 12.5 mmol) in 15 mL acetonitrile. Then triethylamine (101 mg, 1 mmol) was added and the resulting solution was stirred for 2 hours at room temperature. The solvent was removed *in vacuo*, and the residue was purified by flash chromatography on silica gel, eluted with EtOAc:hexane gradually from 1:10 to 1:4 to give the desired product colorless oil (176 mg, 90%). TLC (R_f = 0.5, EtOAc:hexane=1:4). ^{1}H NMR (400 MHz, $CDCl_3$) δ 3.80 (q, *J*=5.9 Hz, 2H), 3.21 (t, *J*=4.8 Hz, 2H), 2.04 (t, *J*=6.2 Hz, 1H). ^{19}F NMR (376 MHz, $CDCl_3$) δ -132.38- -132.48 (m, 2F), -187.10- -137.25 (m,2F), -137.44- -137.60 (m, 2F), -149.80- -149.93 (m, 1F), -160.27- -160.42 (m, 2F).

3. Kinetics Study

The reactions were carried out with 200 mM phosphate, 20 mM TCEP at 37 °C unless otherwise noted. To measure the second order rate constants, reaction mixture was prepared on ice and divided into several 10-μL aliquots. All aliquots were immediately put in 37 °C water bath unless otherwise noted. For reactions that takes more than 1 hour to monitor, all aliquots were heated in a PCR machine set at 37 °C to prevent solvent evaporation. Reactions were quenched by addition of 100 μL 50% water: 50% acetonitrile: 0.5% TFA at different time points and then subjected to LC-MS analysis. The initial concentration of probe and substrate were known. The second-order rate constants were determined by fitting the following kinetics equation:

$$y = \frac{ln\frac{[peptide]_0[probe]_t}{[peptide]_t[probe]_0}}{[probe]_0 - [peptide]_0} = k_2 t$$

Error of reaction rate constant was obtained from the linear fitting of the kinetics curves for measuring the reaction rate constants.

4. Determination of the standard enthalpy/entropy of activation

The secondary rate constant (*k*) for the reaction between π-clamp peptide and probe **2** was experimentally measured at different temperatures (T). Then ln(*k*/T) was plotted against 1/T. The standard enthalpy of activation ($\Delta H^‡$) and the standard entropy of activation ($\Delta S^‡$) were calculated (Table S5) by fitting ln(*k*/T) against 1/T with the following Eyring equation:

$$ln\frac{k}{T} = ln\frac{\kappa k_B}{h} + \frac{\Delta S^‡}{R} + \frac{-\Delta H^‡}{R}\frac{1}{T}$$

where κ is transmission coefficient ($\kappa = 1$), k_B is Boltzmann constant, h is Planck's constant, T is absolute temperature and R is gas constant. The errors for $\Delta H^‡$ and $\Delta S^‡$ were obtained from the linear fitting. $\Delta G^‡$ was calculated as $\Delta H^‡ - T\Delta S^‡$, and the error for $\Delta G^‡$ was calculated from error propagation. The Eyring plots were summarized in Fig. S37.

5. Computation Studies

Density functional theory DFT was used to calculate the reaction free energy of the reaction between perfluoroaryl probe and π-clamp peptide, based on π-clamp representative structures observed in previous molecular dynamics (MD) sampling. All DFT computations were carried out using the Q-Chem 4.1[3] software package. To reduce the computational cost in the DFT calculation, the calculation only involved the 4-residue π-clamp sequence (FCPF; the rest of the peptide **1A** was not involved) and the perfluoroaromatic probe. The free energy (ΔG) was calculated as: $\Delta G = E_{Product} + E_{HF} - E_{Peptide}$ -

$E_{\text{Perfluoroaromatics}}$, where E_{product} is the energy of arylated product, E_{HF} is the energy of hydrogen fluoride, E_{peptide} is the energy of cysteine peptides, and $E_{\text{perfluoroaromatics}}$ is the energy of perfluoroaryl probe.

To calculate the free energy of peptides with 5,5-dimethylproline (5,5-dmP) in DFT, we extracted 4 snapshots from MD simulations with different starting structures of cis-proline peptides and manually added two methyl groups to form 5,5-dmP. Similarly, for starting structures of peptides with α-methylproline (αMePro), we extracted 3 snapshots from MD simulations of trans-proline peptides and manually added a methyl group to form αMePro. For the product's starting structure, we manually connected the perfluoroaryl group to the peptide cysteine.

In each case, four gas-phase geometry optimizations were performed on structures sampled from the MD trajectory, using the B3LYP exchange-correlation functional[4] in the 6-31G* basis set[5]. To account for potential π-π interactions, we also include Grimme's DFT-D3 empirical dispersion correction[6] for the optimization. Once a potential energy minimum was located, we refined the energy by performing a single point energy calculation with the more accurate combination of the rPW86 exchange functional[7], the PBE local correlation functional[8], and the VV10 non-local correlation functional[9] to accurately handle the long-range dispersions critical to the π-π interaction. For these calculations, we also employed the larger 6-31G** basis set[5] and a large non-local integration grid[10]. We then calculated the binding energies in both the gas phase and in water. We approximate the latter by the polarizable continuum model (PCM)[11], for which we used 302 PCM grid points and a dielectric of 78.39. The calculated free energy results are summarized in Table S3.

6. Cysteine pK_a measurement

Absorbance at 240 nm was measured in solutions containing 0.05 mM peptide and 20 mM buffer. Following buffers and pH values are used: MES buffers at pH 5.1, 5.8, and 6.3; phosphate buffers at pH 7.1 and 7.8; Tris buffers at pH 8.1 and 8.6; borate buffers at pH 9.0 and 9.6; and CAPS buffer at pH 10.3 and 10.9.

The reaction rate between **1F** and **2** at different pH were measured for the determination of cysteine pKa in peptide **1F**. Reaction conditions: 100 mM phosphate (for pH 7.2, 7.67 an 8.05) or MES (for pH 5.5, 6.0 and 6.4), 20 mM TCEP, 37 °C. Although the reaction rates were measured in the presence of two different salts, we assumed the changing from phosphate to MES did not significantly influence the reaction. The rate constants at pH 6.4 with MES and phosphate are 3.8 ± 0.2 $M^{-1}s^{-1}$ and 4.3 ± 0.2 $M^{-1}s^{-1}$ respectively, supporting our assumption.

7. Solid State NMR Studies

a. Sample preparation

Peptide **5** was synthesized by solid phase peptide synthesis (SPPS) as described before. To prepare peptide **6**, peptide **5** (0.09 mmol) was dissolved in 6 mL acetonitrile, followed by the addition of probe **2'** (0.2 mmol) and triehtylamine (1 mmol). The mixture was stirred at room temperature for 2h and filtered. The precipitation containing peptide **6** was collected, re-dissolve in 90%A, 10% B and peptide **6** was purified by reverse phase HPLC. Peptide **7** and **8** were prepared in a similar way from peptide **7-Cys** and **8-Cys**.

In order to minimize the interfering ^{19}F signal in REDOR experiment, the trifluoroacetic acid counter ion was exchanged to acetic acid counter ion by the following procedure. The lyophilized peptides **5**, **6**, **7** and **8** were dissolved in 99% water, 1% acetic acid and loaded to Superclean LC-4 SPE (solid phase extraction) columns. The columns were then washed with 100 column volumes of 98% water, 1% acetic acid, 1% acetonitrile to exchange the counter ion. The peptides were eluted with 50% water, 50% acetonitrile, 0.1% acetic acid and lyophilized.

Before solid-state NMR experiments, peptides **6**, **7** and **8** were mixed with a few drops of buffer containing 5 mM phosphate (pH 8.0) to form semi-dry samples. Peptide **5** was measured in dry state, due to unfavorable dynamics of this very soluble peptide in a hydrated state.

b. Solid-state NMR experiments

Solid-state NMR experiments were carried out on a 400 MHz (9.4 T) spectrometer using a 4 mm $^{1}H/^{19}F/^{13}C$ probe and on an 800 MHz (18.8 T) spectrometer using a 3.2 mm $^{1}H/^{13}C/^{15}N$ probe. Typical radiofrequency (rf) field strengths were 71-100 kHz for ^{1}H, 71 kHz for ^{19}F, 50-71 kHz for ^{13}C, and 36-42 kHz for ^{15}N. Chemical shifts were referenced to the CH_2 signal of adamantane at 38.48 ppm on the TMS scale for ^{13}C, and the amide signal of ^{15}N-acetylvaline at 122.0 ppm on the liquid ammonia scale for ^{15}N.

1D ^{13}C and ^{15}N cross-polarization (CP) magic-angle spinning (MAS) spectra were measured at 298 K under 16.5 kHz MAS. 2D ^{13}C-^{13}C correlation spectra were measured using a ^{1}H-driven spin-diffusion (PDSD) experiment with a mixing time of 30 or 50 ms, at 298 K under 11 kHz or 16.5 kHz MAS. 2D ^{15}N-^{13}C heteronuclear correlation (HETCOR) spectra were measured at 298 K under 16.5 kHz MAS using a REDOR period of 1.1 ms for polarization transfer.[12] These spectra were measured on the 800 MHz spectrometer to obtain high resolution. ^{13}C-^{19}F REDOR experiments for measuring ^{13}C-^{19}F

distances between the fluorinated tag and ^{13}C-labeled peptide were carried out at 243 K under 8 kHz MAS on the 400 MHz spectrometer. The REDOR mixing times ranged from 1 ms to 8 ms. ^{19}F 90˚-180˚-90˚ composite pulses were used to compensate for B_1 field inhomogeneity, while a ^{13}C selective Gaussian pulse of two rotor periods (250 μs) was used for ^{13}C refocusing.

c. Torsion angle prediction, REDOR simulations and structural modeling

^{13}C and ^{15}N chemical shifts of the FCPF segment of the peptide were compiled and used as input in the TALOSN program[13] to predict the backbone (ϕ, ψ) torsion angles for each conformation of the PFA-bound π-clamp peptide **6**. The predicted torsion angles do not vary significantly between different forms, so the (ϕ, ψ) torsion angles were averaged and used as a starting point for further refinement of the side chain torsion angles.

The ^{13}C-^{19}F REDOR S/S_0 dephasing curves provide information on the distances between ^{13}C and ^{19}F labeled sites, thus restraining the position of the perfluoroaromatic tag with respect to the peptide. The PFA tag has a total of eight ^{19}F atoms, which makes *de novo* determination of ^{13}C-^{19}F distances through 9-spin simulations time-prohibitive. The experimental data points were first compared to two spin ^{13}C-^{19}F REDOR simulations. A set of simulated REDOR curves was generated using the dipolar coupling between one ^{13}C and one ^{19}F atom as input, corresponding to distances in the range of 1-12 Å in 0.1 Å increments (111 total curves). For each curve, the RMSD was calculated for each experimental REDOR data set, and the minimum RMSD then corresponds to the best fit two-spin ^{13}C-^{19}F distance (**Fig. S51**).

We next ran 5-spin SIMPSON simulations to see if we could better capture the complex nature of the experimental spin system, and adopted a model-dependent approach to determine the optimal side chain conformations that agree with the measured REDOR data. With the backbone fixed to the chemical-shift constrained (ϕ, ψ) angles, we iteratively rotated the relevant sidechain dihedral angles and extracted the corresponding ^{13}C-^{19}F distances and atom positions. In the present case, the ψ and χ_1 angle of Phe1, χ_1, χ_2 and χ_3 of Cys2 and χ_1 of Phe4 were varied. The Phe1 ψ angle was rotated since Phe1 is the N-terminal residue of the peptide, and as such is not predicted by TALOSN. Cysteine does not usually have a χ_2 or χ_3 angle, but these are now present due to the PFA tag being attached. Since some of the ^{13}C signals overlap in the fully labeled peptide, peptides **7** and **8** were synthesized, one with only Phe1 uniformly labeled and the second with Pro3 and Phe4 uniformly labeled. For all six of the angles that were varied, each angle was set at either 60°, 180° or -60° based on the most common

rotamers[14], yielding a total of 729 dihedral angle combinations. With each combination of six dihedral angles, the extracted ^{13}C-^{19}F distances and relative ^{13}C-^{19}F dipolar orientation angles were used in 5-spin REDOR simulations using the SIMPSON program.[15] The five spins in the simulation reflect the ^{13}C site of interest and the four nearest-neighbor ^{19}F on the PFA tag. The resulting REDOR dephasing curves from these geometries were then compared with the experimental REDOR data, and the RMSD between the simulated and measured S/S_0 was calculated for each of the carbon and added together to obtain a total RMSD score for each angle combination.

Reference

1. An, S. S. a; Lester, C. C.; Peng, J. L.; Li, Y. J.; Rothwarf, D. M.; Welker, E.; Thannhauser, T. W.; Zhang, L. S.; Tam, J. P.; Scheraga, H. a. *J. Am. Chem. Soc.* **1999**, *121*, 11558.

2. Zhang, C.; Spokoyny, A. M.; Zou, Y.; Simon, M. D.; Pentelute, B. L. *Angew. Chemie - Int. Ed.* **2013**, *52*, 14001.

3. Shao, Y., et al, M., *Phys. Chem. Chem. Phys.* **2006**, 8, 3172.

4. Becke, A. D., *J. Chem. Phys.* **1993**, 98, 1372.

5. Hariharan, P. C.; Pople, J. A., *Theoret. Chim. Acta* **1973**, 28, 213.

6. Grimme, S.; Antony, J.; Ehrlich, S.; Krieg, H., *J. Chem. Phys*. **2010**, 132, 154104.

7. Murray, É. D.; Lee, K.; Langreth, D. C., *J. Chem. Theory Comput*. **2009**, 5, 2754.

8. Perdew, J. P.; Burke, K.; Ernzerhof, M., *Phys. Rev. Lett.* **1996**, 77, 3865.

9. Vydrov, O. A.; Van Voorhis, T., *J. Chem. Phys.* **2010**, 133, 244103.

10. Lebedev, V. I., Sibirsk. *Mat. Zh.* **1977**, 18, 132.

11. Cossi, M.; Rega, N.; Scalmani, G.; Barone, V., *J. Comput. Chem.* **2003**, 24, 669.

12. Hong, M. and R. G. Griffin. *J. Am. Chem. Soc.* **1998**, 120, 7113.

13. Shen, Y. and A. Bax *J. Biomol. NMR*, **2013**, 56, 227.

14. Lovell, S. C., et al. *Proteins*, **2000**, 40: 389-408.

15. Bak, M., et al. *J. Magn. Reson.* **2000**, 147, 296.

Supplementary Tables and Figures

Table S1. Sequences and masses of peptides

Peptide	Sequence	Note	Calc. mass	Obs. mass
**1A**	NH_2-FCPFGLLKNK-$CONH_2$		1164.65	1164.65
**1B**	NH_2-FCXFGLLKNK-$CONH_2$	X=L-α–methylproline	1178.66	1178.66
**1C-pro**	NH_2-FC(S-StBu)XFGLLKNK-$CONH_2$	X=L-5,5-dimethylproline	1280.71	1280.71
**1C**	NH_2-FCXFGLLKNK-$CONH_2$	X=L-5,5-dimethylproline	1192.68	1192.68
**1D**	NH_2-LCPLGLLKNK-$CONH_2$		1096.68	1096.68
**1E**	NH_2-XCPXGLLKNK-$CONH_2$	X=cyclohexylalanine	1176.74	1176.74
**1F**	NH_2-XCPXGLLKNK-$CONH_2$	X=3-pyrenyl-L-Ala	1412.71	1412.71
**1G**	NH_2-XCX'XGLLKNK-$CONH_2$	X=3-pyrenyl-L-Ala<br>X'=L-α–methylproline	1426.73	1426.73
**2-Cys**	NH_2-VTLPSTCGAS-$CONH_2$		933.46	933.46
**2**	NH_2-VTLPSTC'GAS-$CONH_2$	C'= Cys ($C_{12}F_9$)	1247.44	1247.44
**3**	NH_2-GCPGGLLKNK-$CONH_2$		984.55	984.55
**4A**	NH_2-XSPXGLLKNK-$CONH_2$	X=3-pyrenyl-L-Ala	1396.73	1396.73
**4B**	NH_2-GSPGGLLKNK-$CONH_2$		968.58	968.58
**5**	NH_2-F*C*P*F*GLLKNK	F*= U-13C, 15N-L-Phe<br>C*= U-13C, 15N-L-Cys<br>P*= U-13C, 15N-L-Pro	1194.6	1194.7
**6**	NH_2-F*C*($C_{12}F_8$-SC_2H_4OH)P*F*GLLKNK-$CONH_2$		1566.7	1566.7
**7-Cys**	NH_2-F*CPFGLLKNK-$CONH_2$		1174.7	1174.7
**7**	NH_2-F*C($C_{12}F_8$-SC_2H_4OH)PFGLLKNK-$CONH_2$		1546.7	1546.7
**8-Cys**	NH_2-FCP*F*GLLKNK-$CONH_2$		1180.7	1180.7
**8**	NH_2-FC($C_{12}F_8$-SC_2H_4OH)P*F*GLLKNK-$CONH_2$		1552.7	1552.7

*Amino acids are shown in one-letter code.

Table S2. Summary of rate constants for reactions between clamp peptides and probe **2**

Peptide	Rate constant ($M^{-1}s^{-1}$)	Error of Rate constant ($M^{-1}s^{-1}$)
**1A**	0.63	0.02
**1B**	1.55	0.05
**1C**	0.10	0.003
**1D**	0.133	0.003
**1E**	1.67	0.02
**1F**	26.8	1.2
**3**	0.00065	0.00005

*Reaction conditions: 200 mM phosphate, 20 mM TCEP, pH 8, 37 °C. Errors were calculated from linear fitting of kinetics equation.

Table S3: Free energy of reactions with 5,5-dmP and αMePro structures

Structure	PCM Solvated ΔG (kcal/mol)
5,5-dmP_A	-63.30
5,5-dmP _B	-73.89
5,5-dmP _C	-82.55
5,5-dmP _D	-77.24
**5,5-dmP Average**	**-74.22**
αMePro _A	-87.57
αMePro _B	-88.16
αMePro _C	-82.34
**αMePro Average**	**-86.02**

Table S4. Rate constants for reactions between clamp peptides and probe **2** at different temperature

Peptide	T (°C)	Rate ($M^{-1}s^{-1}$)	Peptide	T (°C)	Rate ($M^{-1}s^{-1}$)
**1A**	44.8	1.17	**1B**	44.8	2.83
	37.7	0.62		37.7	1.55
	33.3	0.41		32.7	0.87
	29	0.20		29	0.76
	24.4	0.11		24.4	0.42
**1C**	44.8	0.33	**1D**	44.8	0.27
	37.7	0.098		37.7	0.13
	33	0.075		33.3	0.088
	29	0.045		29	0.041
	24.4	0.025		24.4	0.022
**1E**	44.8	3.45	**1F**	44.3	50.5
	37.7	1.88		37.7	26.8
	33	1.07		33.3	14.9
	29	0.44		29	5.4
	24.4	0.29		24.4	2.77
**3**	48	0.0048			
	45	0.0030			
	41	0.0011			
	39	0.00093			
	37	0.00065			

Table S5. Summary of thermodynamic parameters (310 K) for cysteine arylation reaction.

Peptide	$\Delta H^{\ddagger}$ ($kJ \cdot mol^{-1}$)	$-T\Delta S^{\ddagger}$ ($kJ \cdot mol^{-1}$)	$\Delta G^{\ddagger}$ ($kJ \cdot mol^{-1}$)	k ($M^{-1}s^{-1}$)	Peptide Sequence
**3**	153 ± 13	-58 ± 13	95 ± 18	0.00065	**X**-C-P-**X** (X=Gly)
**1A**	89 ± 6	-11 ± 6	78 ± 8	0.63	F-C-P-F
**1B**	69 ± 4	5 ± 4	75 ± 6	1.55	F-C-**αMePro**-F
**1C**	92 ± 8	-10 ± 8	82 ± 11	0.10	F-C-**5,5-dmP**-F
**1D**	96 ± 6	-14 ± 6	82 ± 8	0.13	**X**-C-P-**X** (X=Leu)
**1E**	98 ± 9	-23 ± 9	75 ± 17	1.67	**X**-C-P-**X** (X=CyclohexylAla)
**1F**	116 ± 10	-48 ± 10	68 ± 14	26.8	**X**-C-P-**X** (X=PyrenylAla)

* $\Delta H^{\ddagger}$ and $\Delta S^{\ddagger}$ were calculated by fitting ln(*k*/T) against 1/T with Eyring equation and the errors were obtained from the linear fitting. $\Delta G^{\ddagger}$ was calculated as $\Delta H^{\ddagger}$ - $T\Delta S^{\ddagger}$.

Table S6. PFA modified π-Clamp Peptide **6** Chemical Shifts

Residue	Chemical Shifts					
	N	CO	Cα	Cβ	Cγ	Cδ
**F1a**	31.5	174.8	59.0	42.7	~137	~130
**F1b**	21.6	175.9	54.1	39.1	~137	~130
**F1c**	30.3	174.2	59.4	43.8	~137	~130
**F1d**	29.2	174.6	59.5	43.3	~137	~130
**C2a**	114.9	167.9	53.2	40.8	-	-
**C2b**	108.8	168.5	53.1	41.2	-	-
**C2c**	114.5	168.8	52.2	40.6	-	-
**C2d**	114.6	169.7	52.1	40.4	-	-
**P3a**	128.7	172.6	61.5	30.8	25.4	47.4
**P3b**	129.9	173.0	62.1	30.5	25.5	48.2
**P3c**	127.7	171.6	61.5	29.2	25.6	47.7
**P3d**	131.4	171.3	61.4	29.3	25.5	48.3
**F4a**	112.2	172.4	52.5	37.7	~138	~133
**F4b**	112.8	172.8	52.8	38.0	~138	~133
**F4c**	121.0	172.7	54.9	40.1	~138	~133
**F4d**	123.7	172.9	53.3	38.1	~138	~133

Table S7. π-Clamp Peptide **5** Chemical Shifts

Residue	Chemical Shifts					
	N	CO	Cα	Cβ	Cγ	Cδ
**F1**	-	174.4	56.5	37.2	~135	~130
**C2**	-	171.9	53.9	38.8	-	-
**P3**$_{cis}$	-	171.9	60.4	34.0	23.7	48.1
**P3**$_{trans}$	-	173.0	61.3	29.8	25.7	48.1
**F4**	-	169.1	54.9	37.4	~135	~130

Supplementary Figures

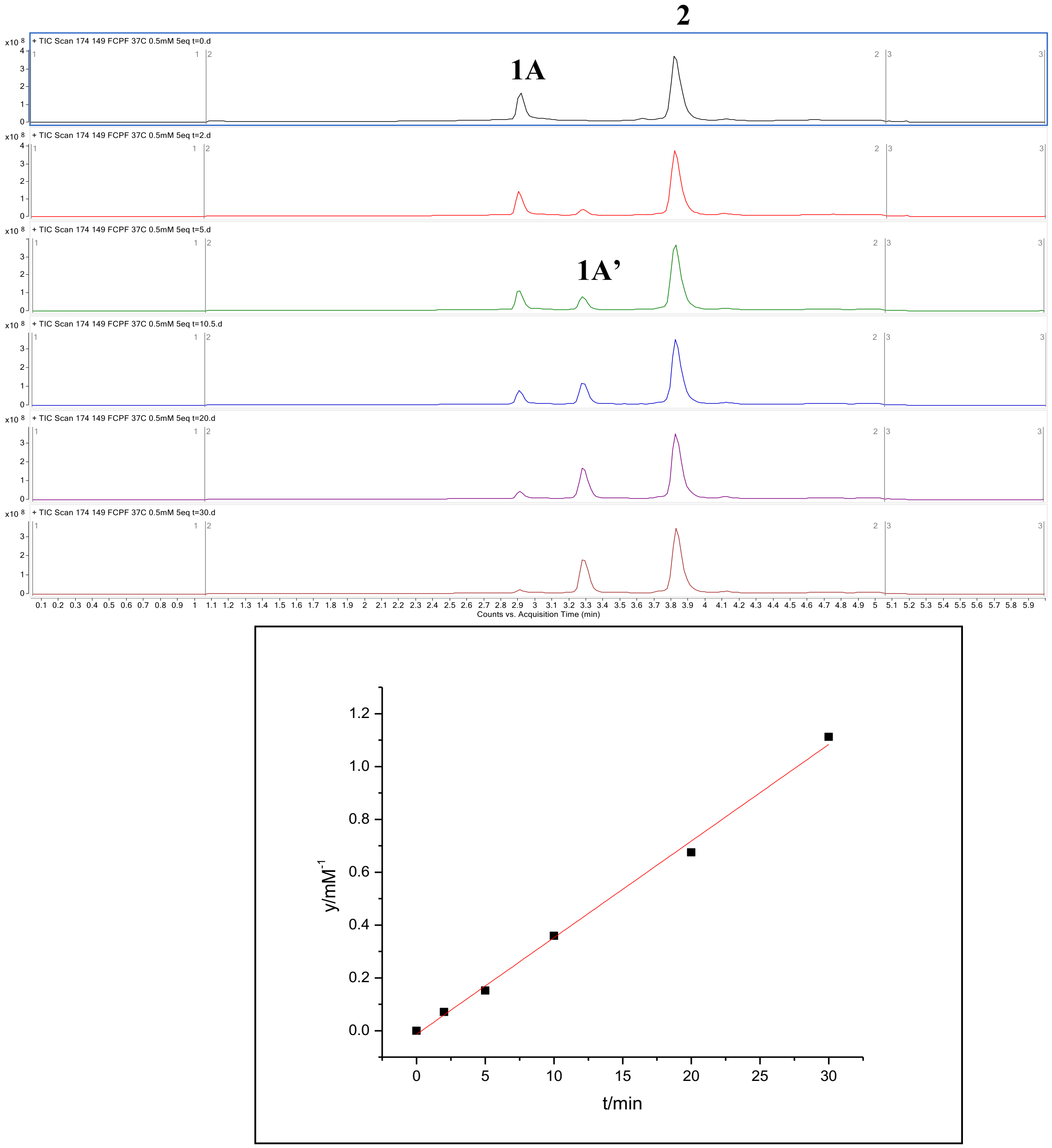

Figure S1. **Kinetics analysis for peptide 1A reacting with probe 2 at 37 °C.** a) LC-MS chromatograms for the reaction between peptide **1A** and probe **2** at different time points. Reaction conditions: 0.5 mM **1A**, 2.5 mM probe **2**, 200 mM phosphate, 20 mM TCEP, pH 8.0, 37 °C. b) Linear fitting of the kinetics data to second-order rate equation. Slope = 0.038, R^2 = 0.996. **1A'** refers to the product of the arylation reaction between **1A** and probe **2**.

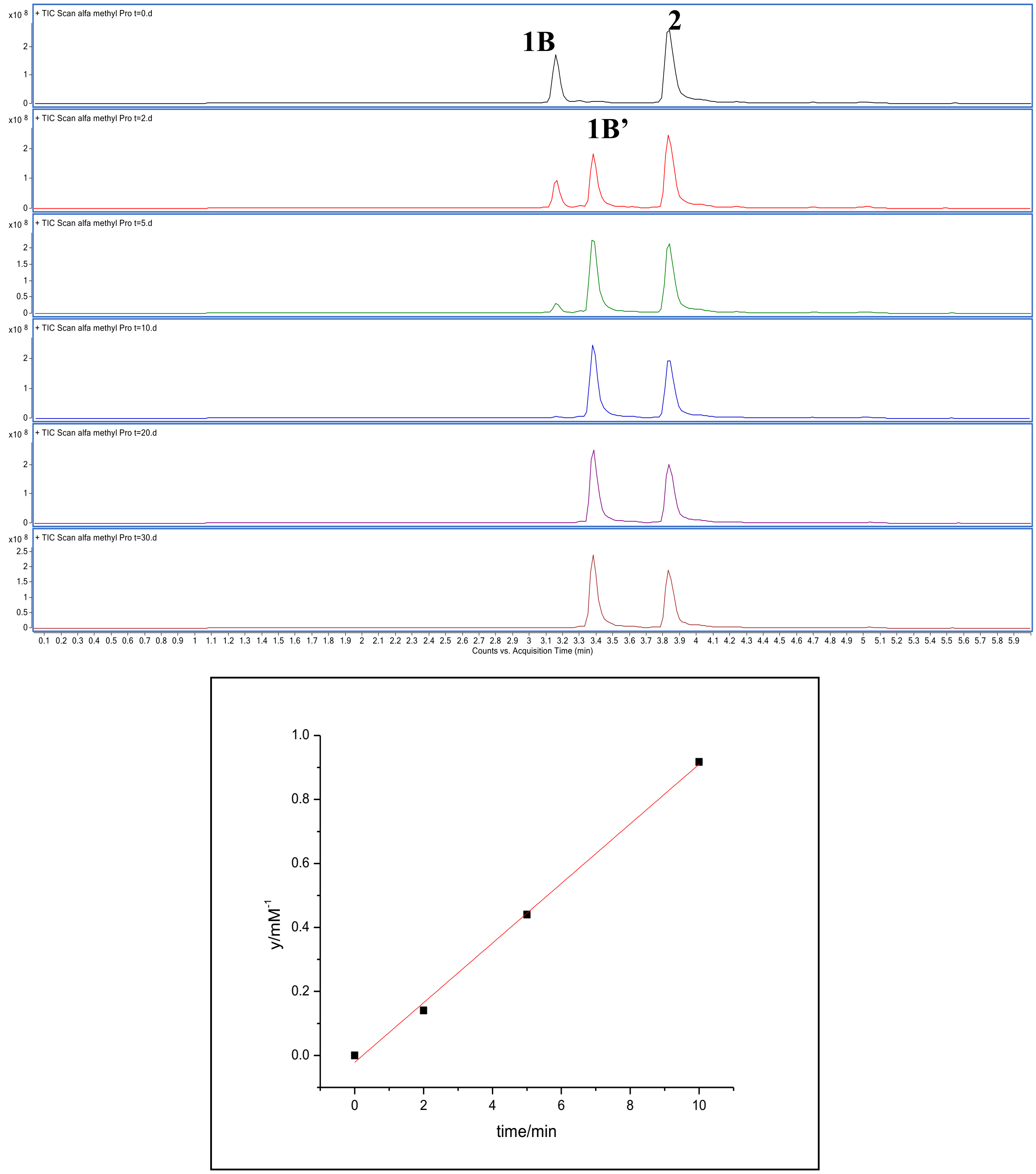

Figure S2. **Kinetics analysis for peptide 1B reacting with probe 2 at 37 °C.** Top, LC-MS chromatograms for the reaction between peptide **1B** and probe **2** at different time points (minutes). Reaction conditions: 1 mM **1B**, 5 mM probe **2**, 200 mM phosphate, 20 mM TCEP, pH 8.0, 37 °C. Bottom, linear fitting of the kinetics data to second-order rate equation. Slope = 0.093, R^2 = 0.996. **1B'** refers to the product of the arylation reaction between **1B** and probe **2**.

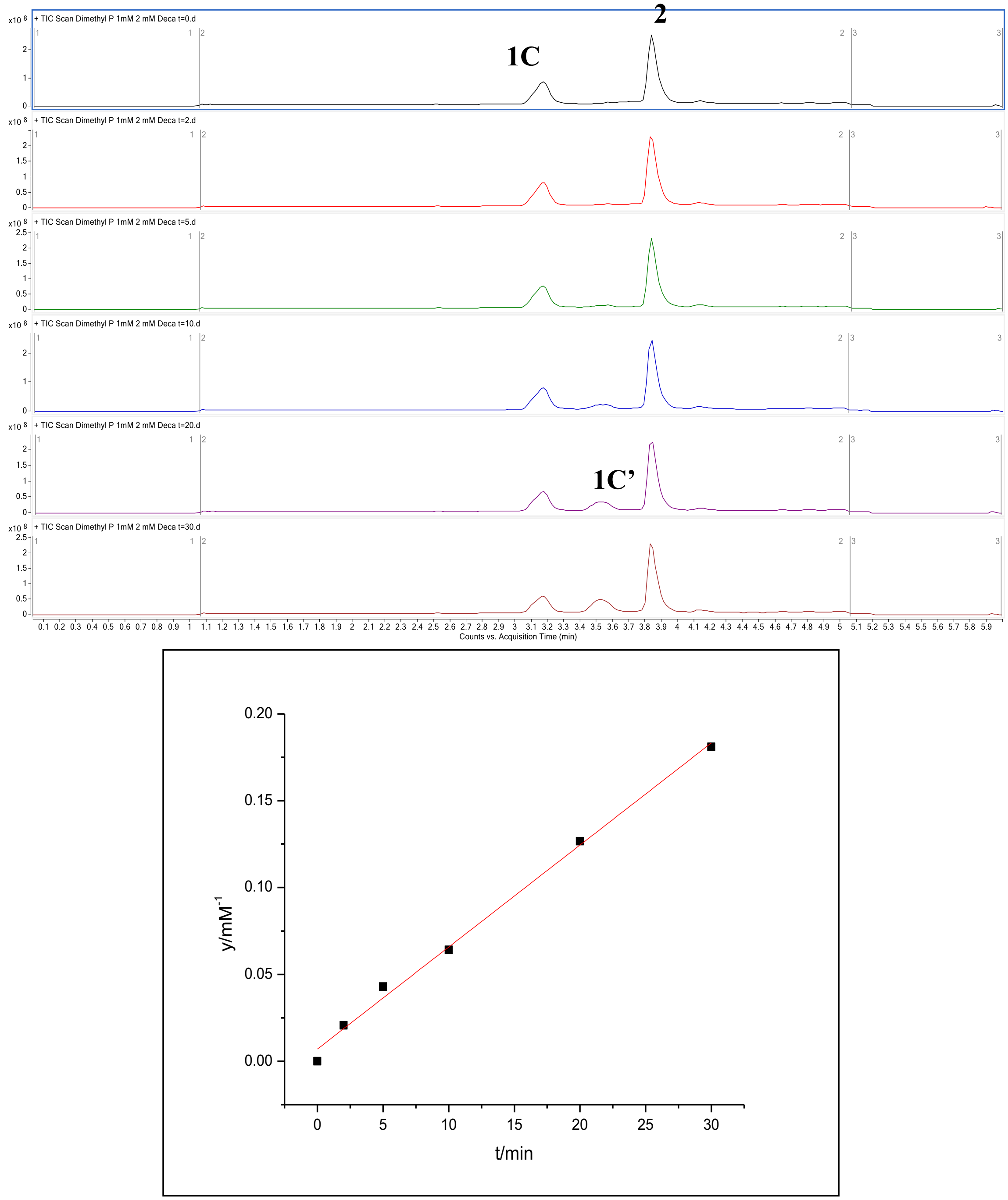

Figure S3. **Kinetics analysis for peptide 1C reacting with probe 2 at 37 °C.** Top, LC-MS chromatograms for the reaction between peptide **1C** and probe **2** at different time points (minutes). Reaction conditions: 1 mM **1C**, 2 mM probe **2**, 200 mM phosphate, 20 mM TCEP, pH 8.0, 37 °C. Bottom, linear fitting of the kinetics data to second-order rate equation. Slope = 0.093, R^2 = 0.996. **1C’** refers to the product of the arylation reaction between **1C** and probe **2**.

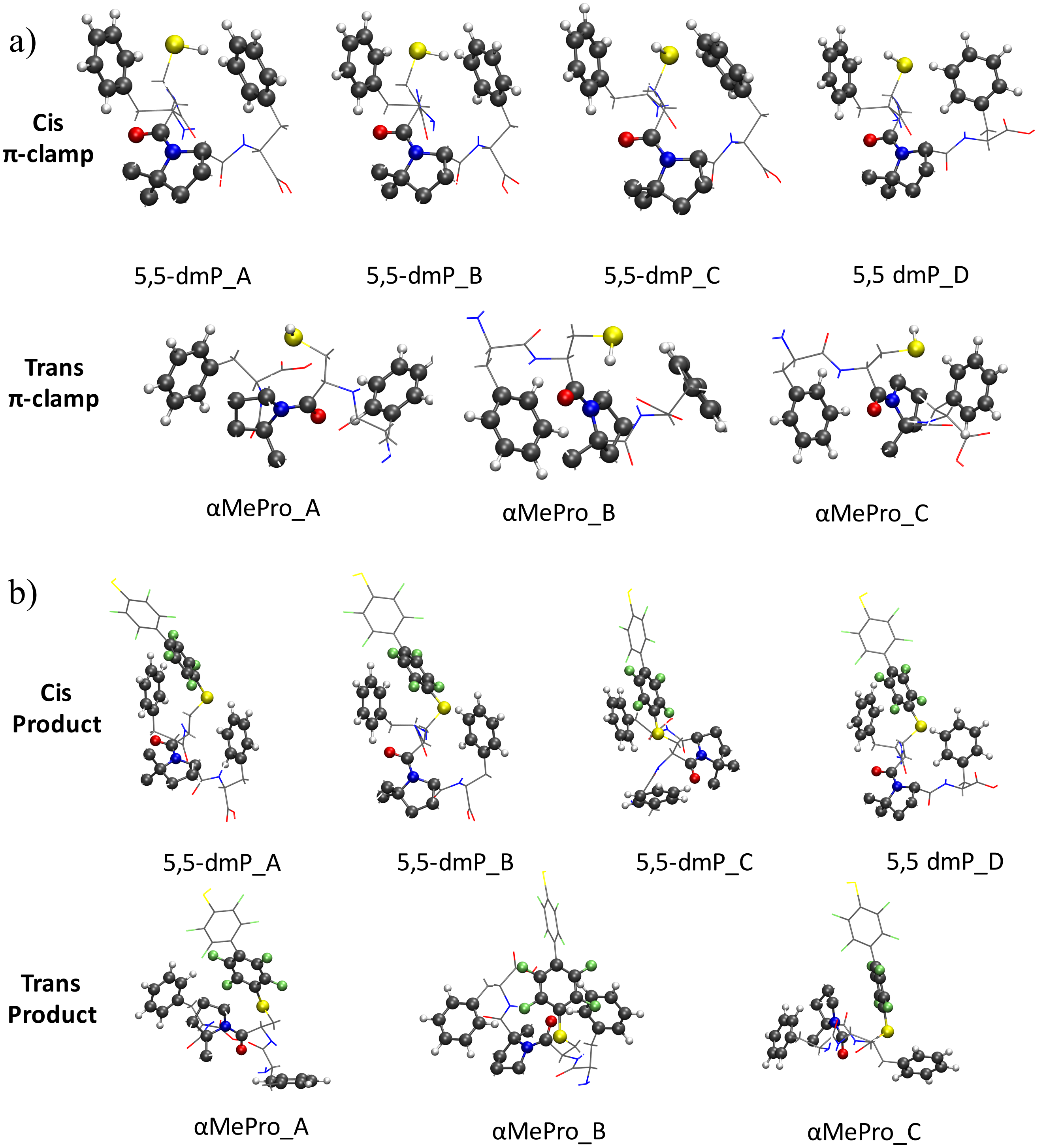

Figure 4. Calculated lowest energy structures for peptide 1B and 1C (a) and their arylation products (b). Several snapshots of starting structures of peptide **1A** from MD simulation were extracted. Methyl group was manually added to form The perfluoroaryl group was manually added, followed with geometry optimization to get lowest energy structures.

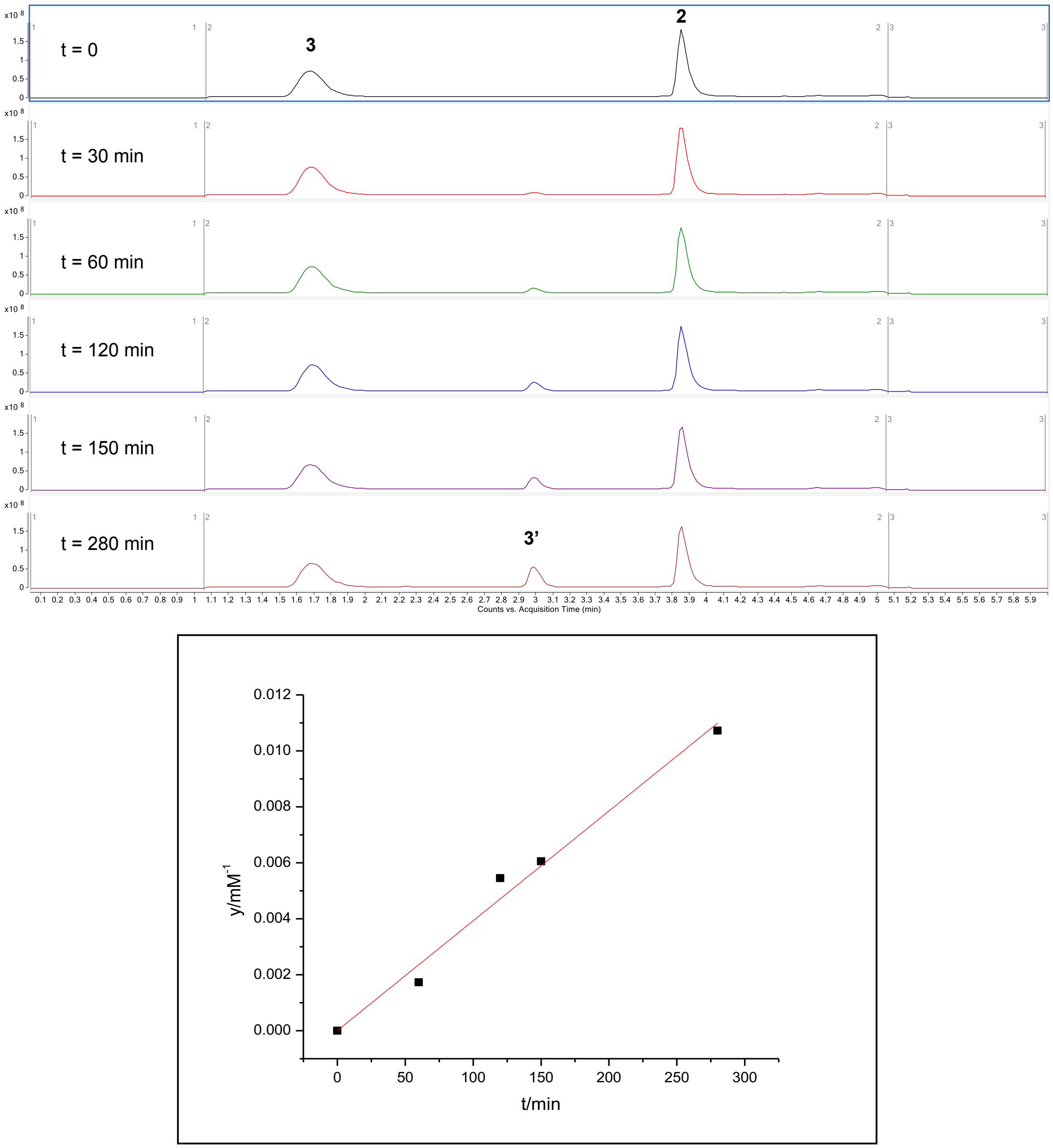

Figure S5. **Kinetics analysis for peptide 3 reacting with probe 2 at 37 °C.** Top, LC-MS chromatograms for the reaction between peptide **3** and probe **2** at different time points. Reaction conditions: 16.7 mM **3**, 11.2 mM probe **2**, 200 mM phosphate, 20 mM TCEP, pH 8.0, 37 °C. Bottom, linear fitting of the kinetics data to second-order rate equation. Slope = 0.000039, R^2 = 0.98. **3’** refers to the product of the arylation reaction between **3** and probe **2**.

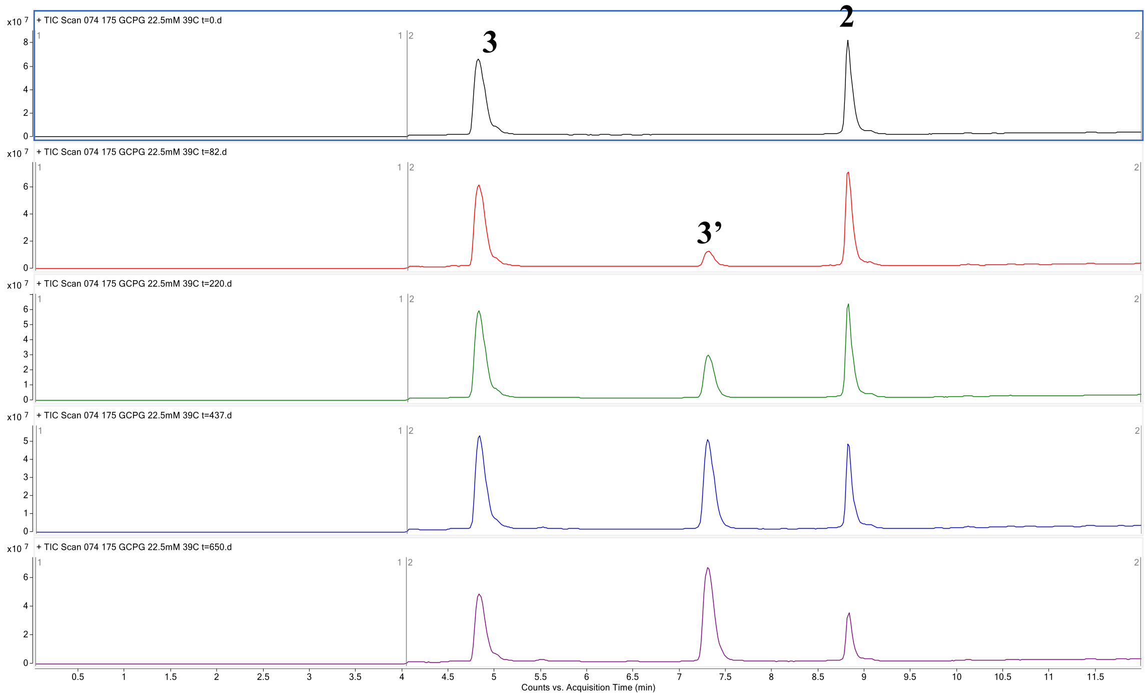

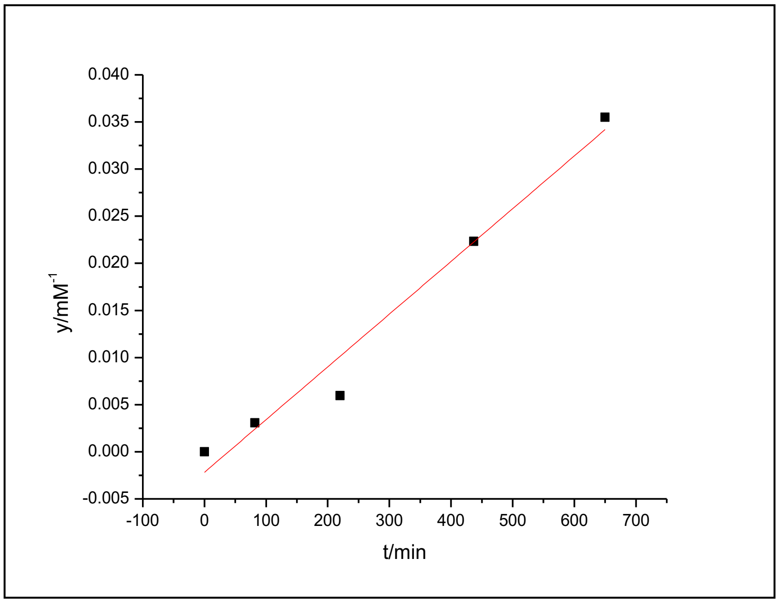

Figure S6. **Kinetics analysis for peptide 3 reacting with probe 2 at 39 °C.** Top, LC-MS chromatograms for the reaction between peptide **3** and probe **2** at different time points (min). Reaction conditions: 22.5 mM **3**, 11.25 mM probe **2**, 200 mM phosphate, 20 mM TCEP, pH 8.0, 39.0 °C. Bottom, linear fitting of the kinetics data to second-order rate equation. Slope = 0.000056, R^2 = 0.992. **3’** refers to the product of the arylation reaction between **3** and probe **2**.

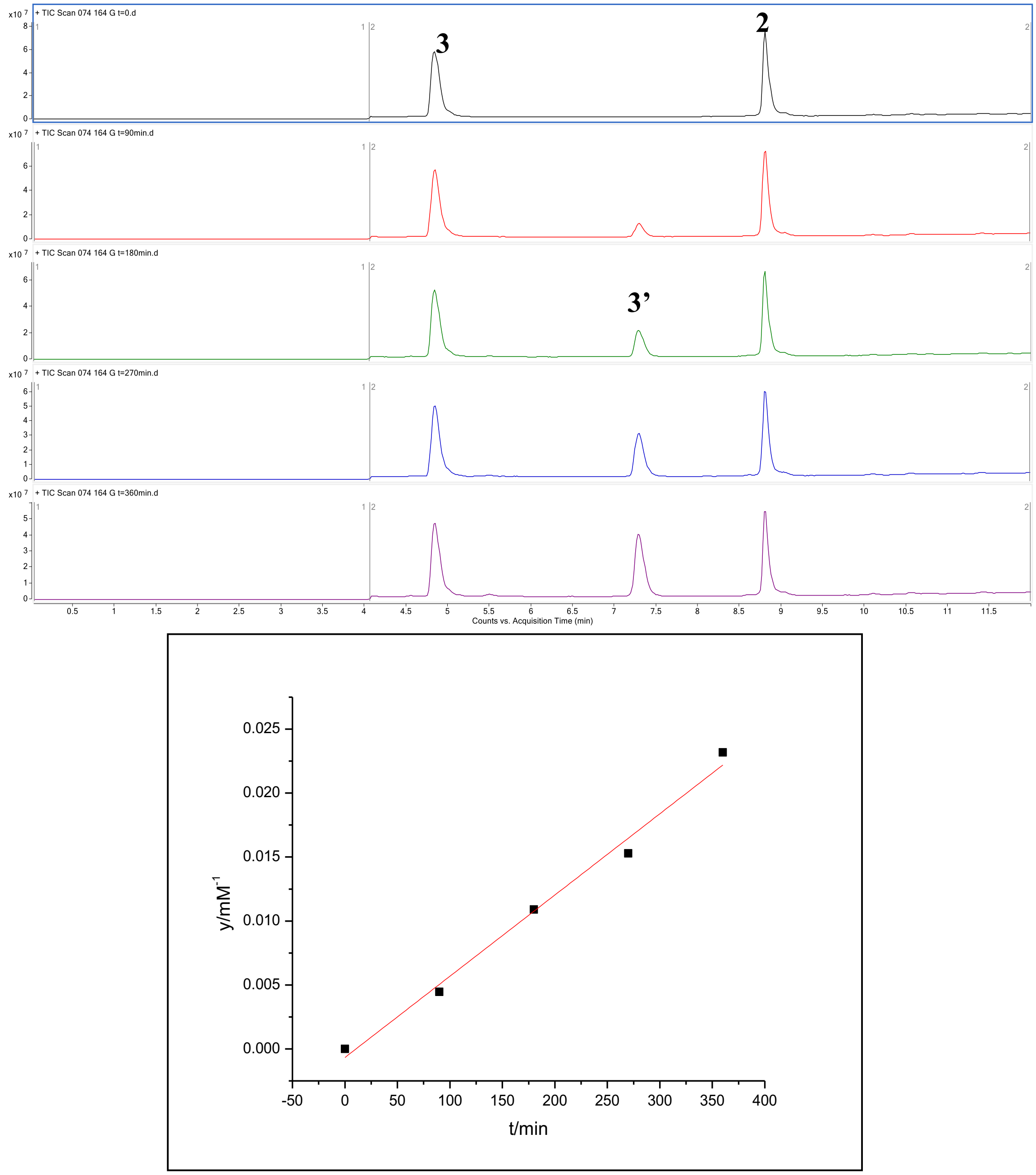

Figure S7. **Kinetics analysis for peptide 3 reacting with probe 2 at 41 °C.** Top, LC-MS chromatograms for the reaction between peptide **3** and probe **2** at different time points. Reaction conditions: 16.7 mM **3**, 11.2 mM probe **2**, 200 mM phosphate, 20 mM TCEP, pH 8.0, 41.0 °C. Bottom, linear fitting of the kinetics data to second-order rate equation. Slope = 0.000064, R^2 = 0.98. **3’** refers to the product of the arylation reaction between **3** and probe **2**.

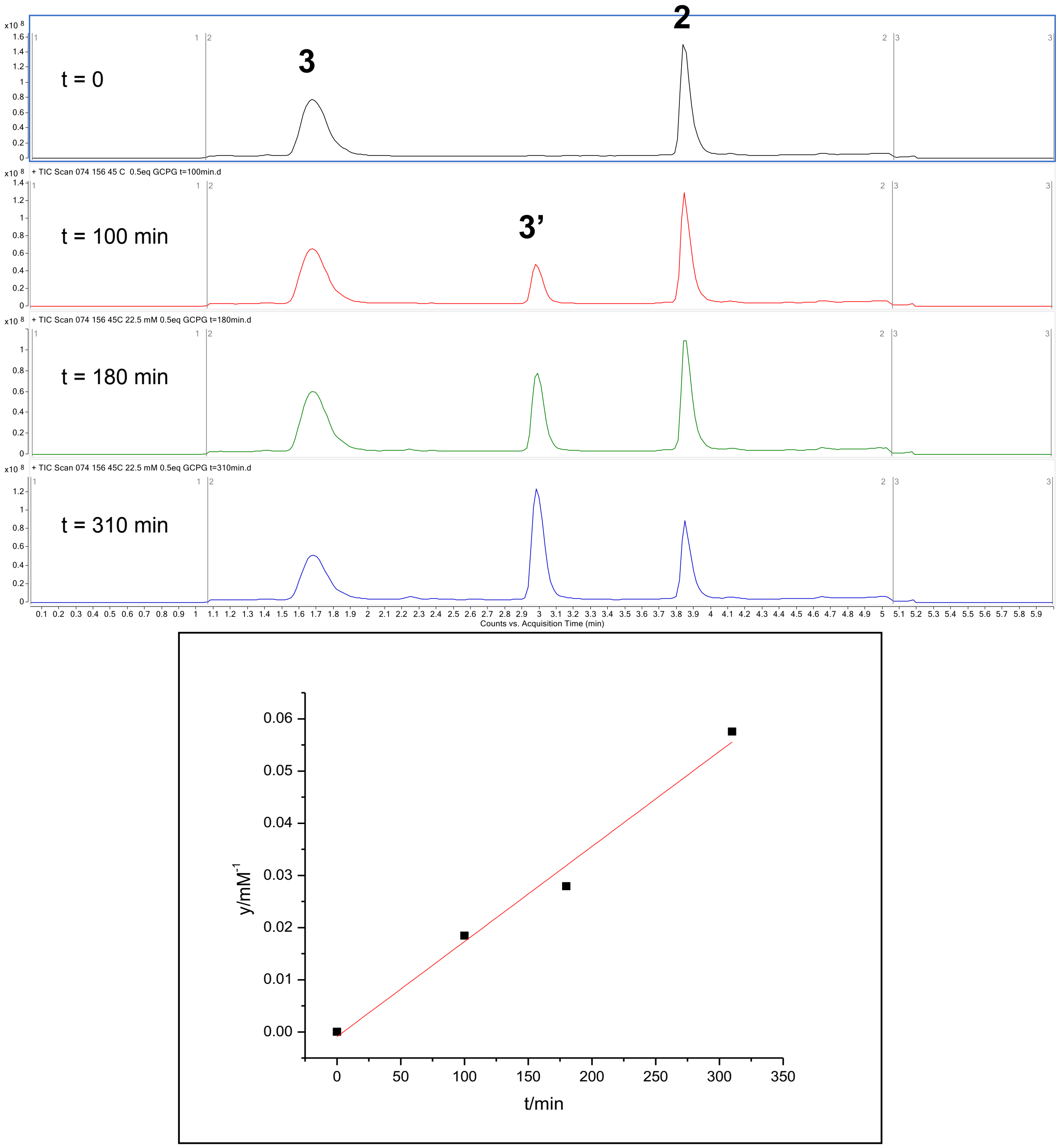

Figure S8. **Kinetics analysis for peptide 3 reacting with probe 2 at 45 °C.** Top, LC-MS chromatograms for the reaction between peptide **3** and probe **2** at different time points. Reaction conditions: 16.7 mM **3**, 11.2 mM probe **2**, 200 mM phosphate, 20 mM TCEP, pH 8.0, 45.0 °C. Bottom, linear fitting of the kinetics data to second-order rate equation. Slope = 0.00018, R^2 = 0.98. **3’** refers to the product of the arylation reaction between **3** and probe **2**.

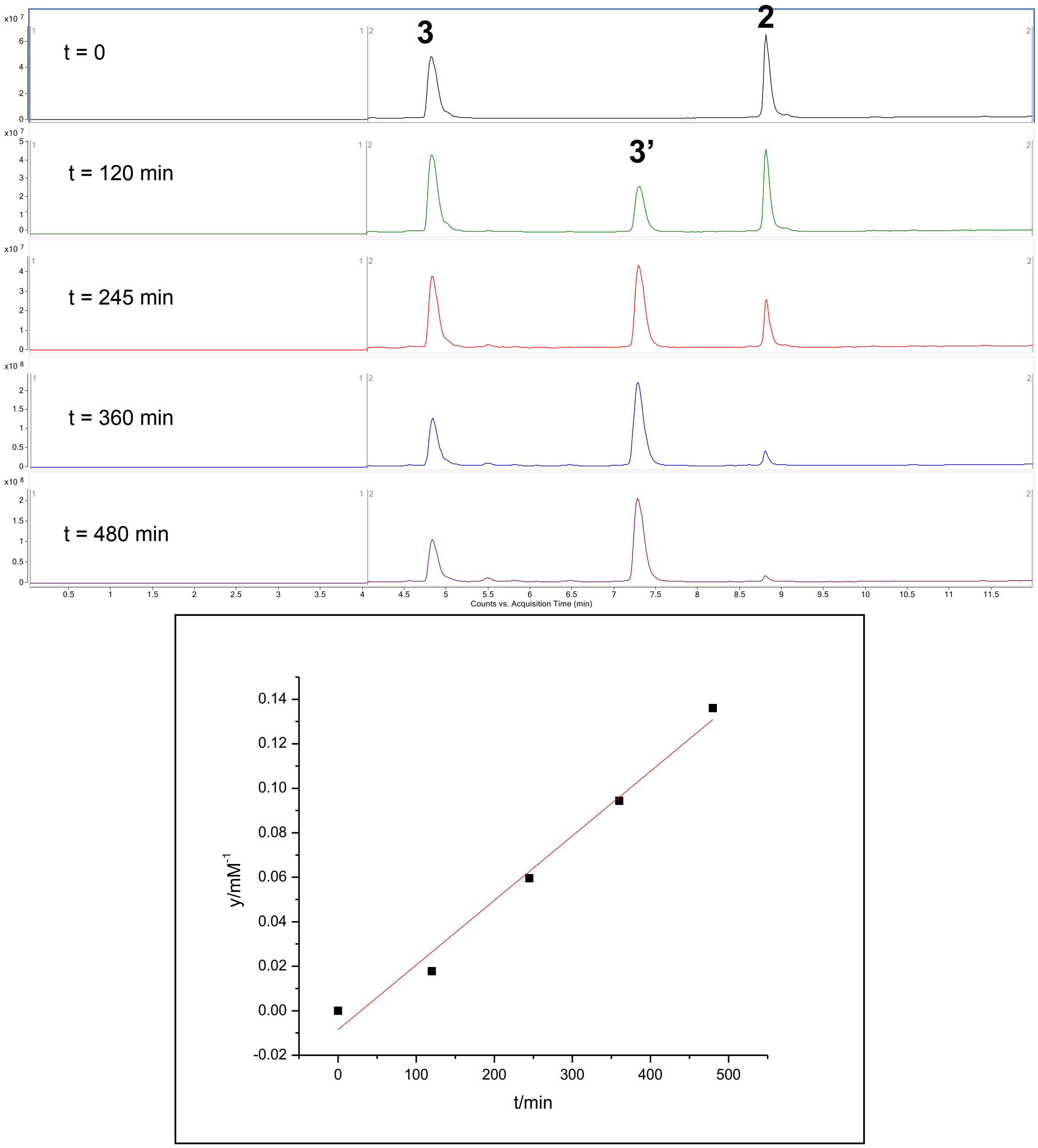

Figure S9. **Kinetics analysis for peptide 3 reacting with probe 2 at 48 °C.** Top, LC-MS chromatograms for the reaction between peptide **3** and probe **2** at different time points. Reaction conditions: 22.5 mM **3**, 11.25 mM probe **2**, 200 mM phosphate, 20 mM TCEP, pH 8.0, 48.0 °C. Bottom, linear fitting of the kinetics data to second-order rate equation. Slope = 0.00029, R^2 = 0.98. **3'** refers to the product of the arylation reaction between **3** and probe **2**.

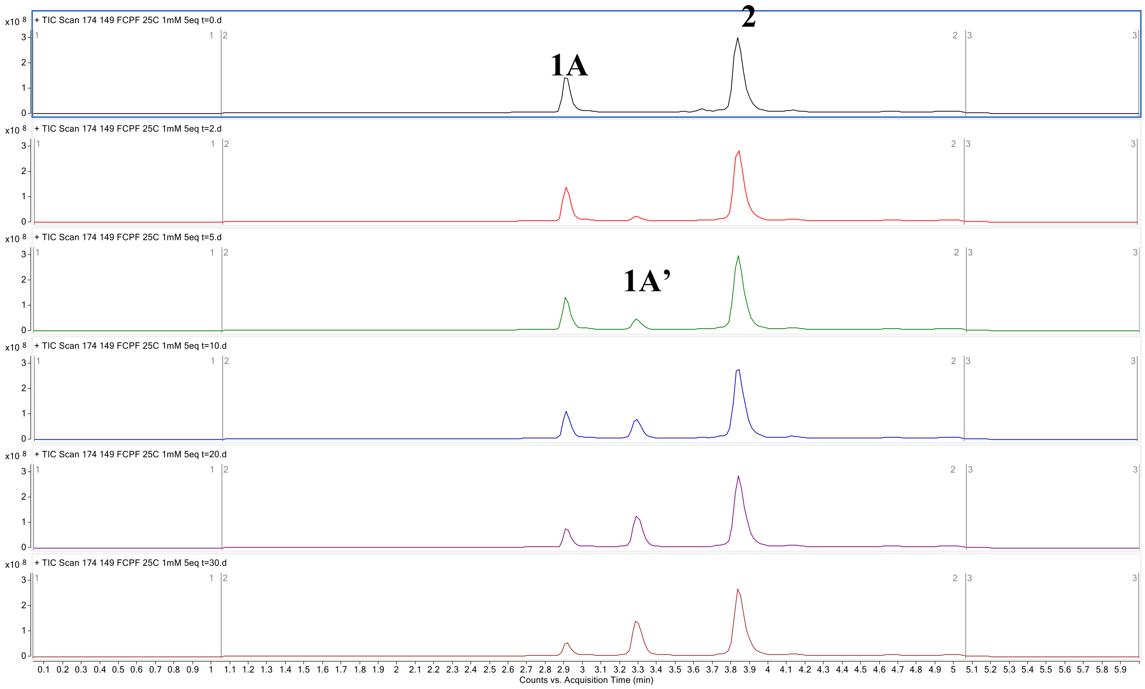

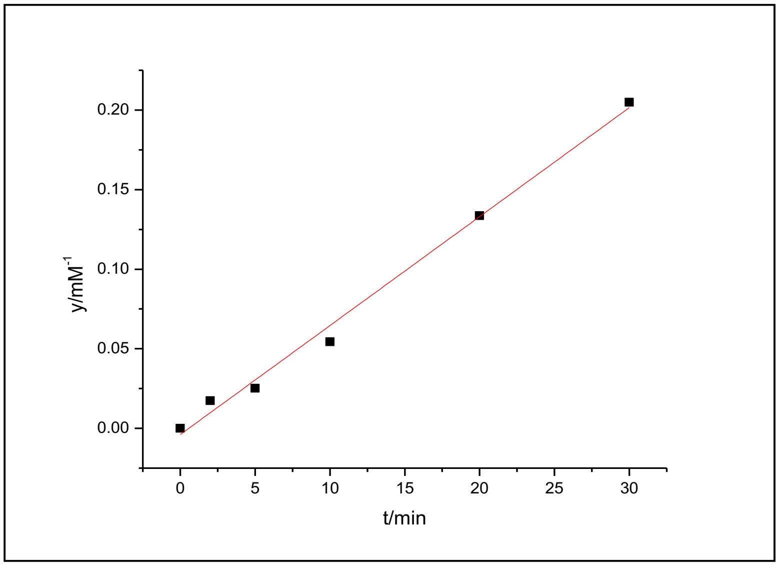

Figure S10. **Kinetics analysis for peptide 1A reacting with probe 2 at 24.4 °C.** Top, LC-MS chromatograms for the reaction between peptide **1A** and probe **2** at different time points (minutes). Reaction conditions: 1 mM **1A**, 5 mM probe **2**, 200 mM phosphate, 20 mM TCEP, pH 8.0, 24.4 °C. Bottom, linear fitting of the kinetics data to second-order rate equation. Slope = 0.0685, R^2 = 0.991. **1A'** refers to the product of the arylation reaction between **1A** and probe **2**.

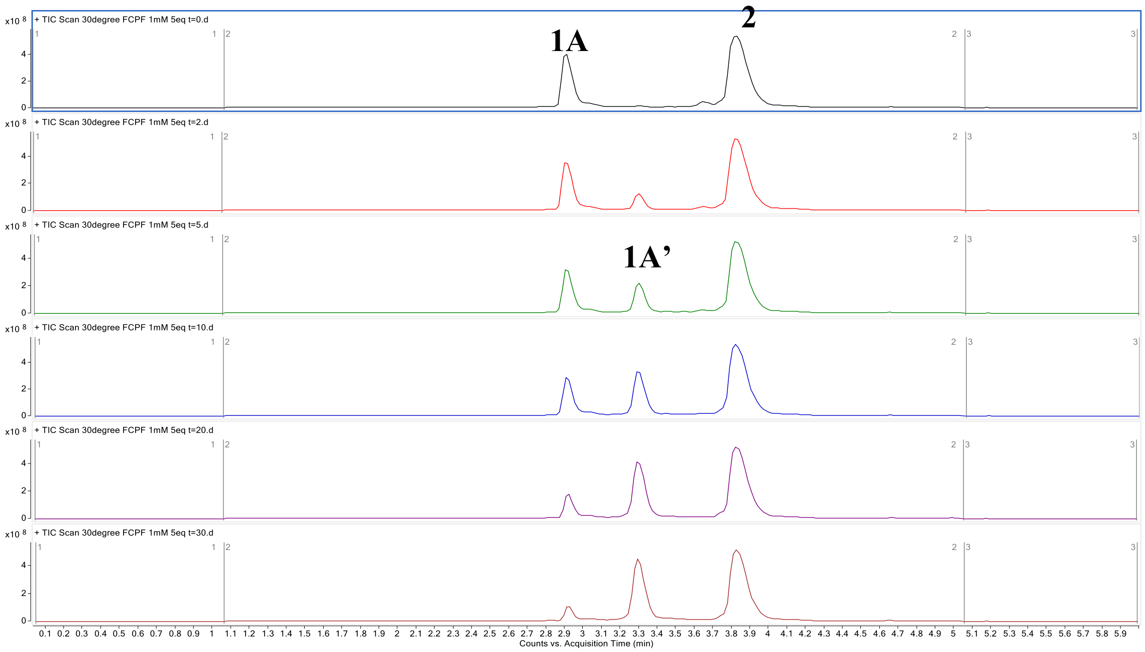

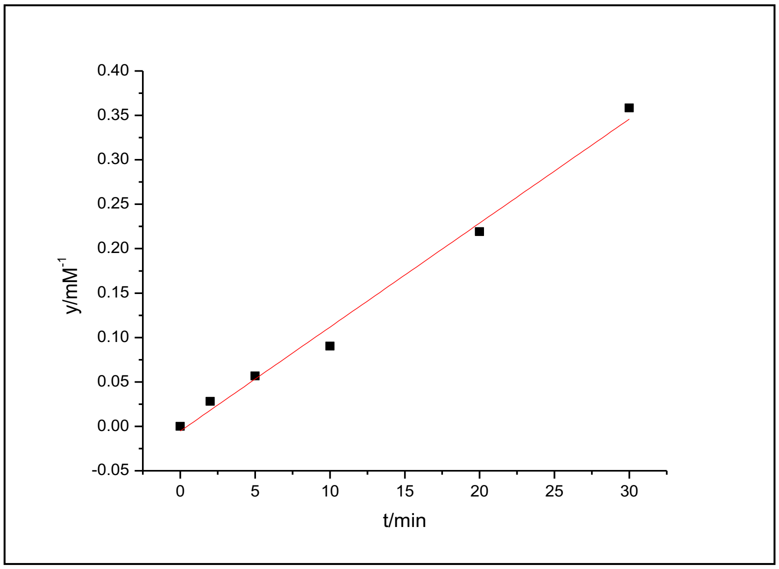

Figure S11. **Kinetics analysis for peptide 1A reacting with probe 2 at 29.0 °C.** Top, LC-MS chromatograms for the reaction between peptide **1A** and probe **2** at different time points (minutes). Reaction conditions: 1 mM **1A**, 5 mM probe **2**, 200 mM phosphate, 20 mM TCEP, pH 8.0, 29 °C. Bottom, linear fitting of the kinetics data to second-order rate equation. Slope = 0.012, R^2 = 0.98. **1A'** refers to the product of the arylation reaction between **1A** and probe **2**.

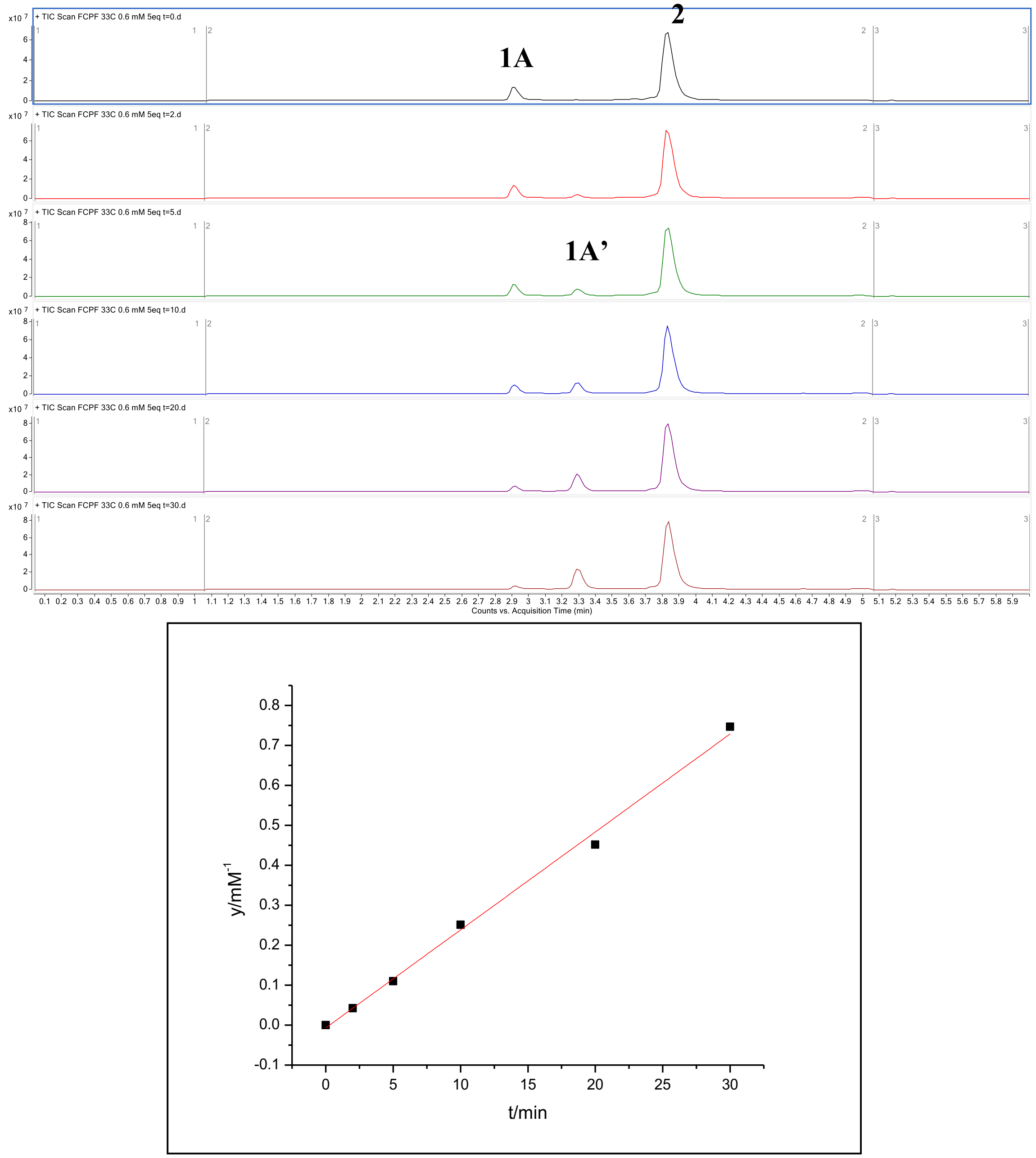

Figure S12. **Kinetics analysis for peptide 1A reacting with probe 2 at 33.3 °C.** Top, LC-MS chromatograms for the reaction between peptide **1A** and probe **2** at different time points (minutes). Reaction conditions: 0.6 mM **1A**, 3 mM probe **2**, 200 mM phosphate, 20 mM TCEP, pH 8.0, 33.3 °C. Bottom, linear fitting of the kinetics data to second-order rate equation. Slope = 0.0245, R^2 = 0.999. **1A'** refers to the product of the arylation reaction between **1A** and probe **2**.

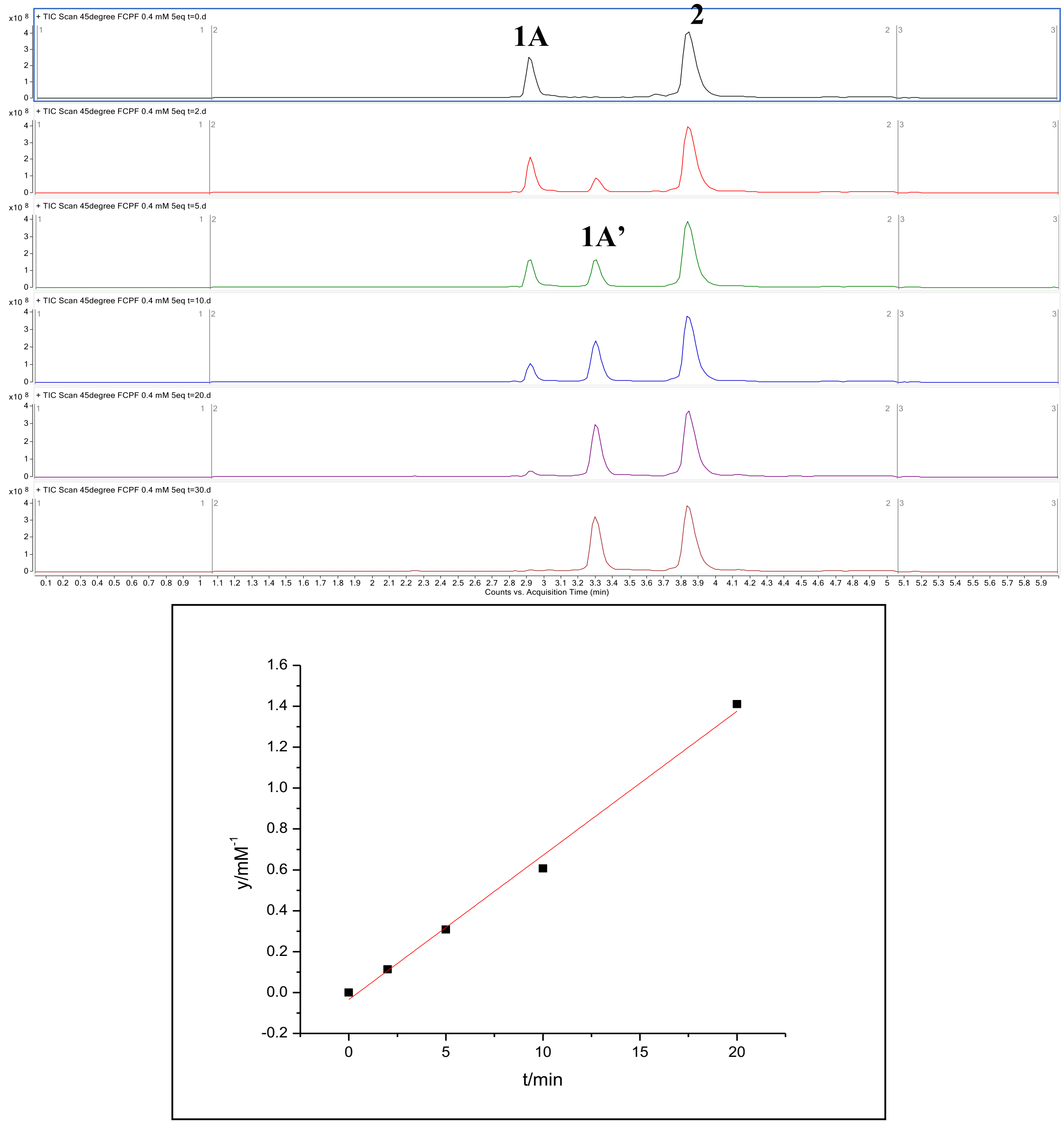

Figure S13. **Kinetics analysis for peptide 1A reacting with probe 2 at 44.8 °C.** Top, LC-MS chromatograms for the reaction between peptide **1A** and probe **2** at different time points (minutes). Reaction conditions: 0.4 mM **1A**, 2 mM probe **2**, 200 mM phosphate, 20 mM TCEP, pH 8.0, 44.8 °C. Bottom, linear fitting of the kinetics data to second-order rate equation. Slope = 0.070, R^2 = 0.993. **1A'** refers to the product of the arylation reaction between **1A** and probe **2**.

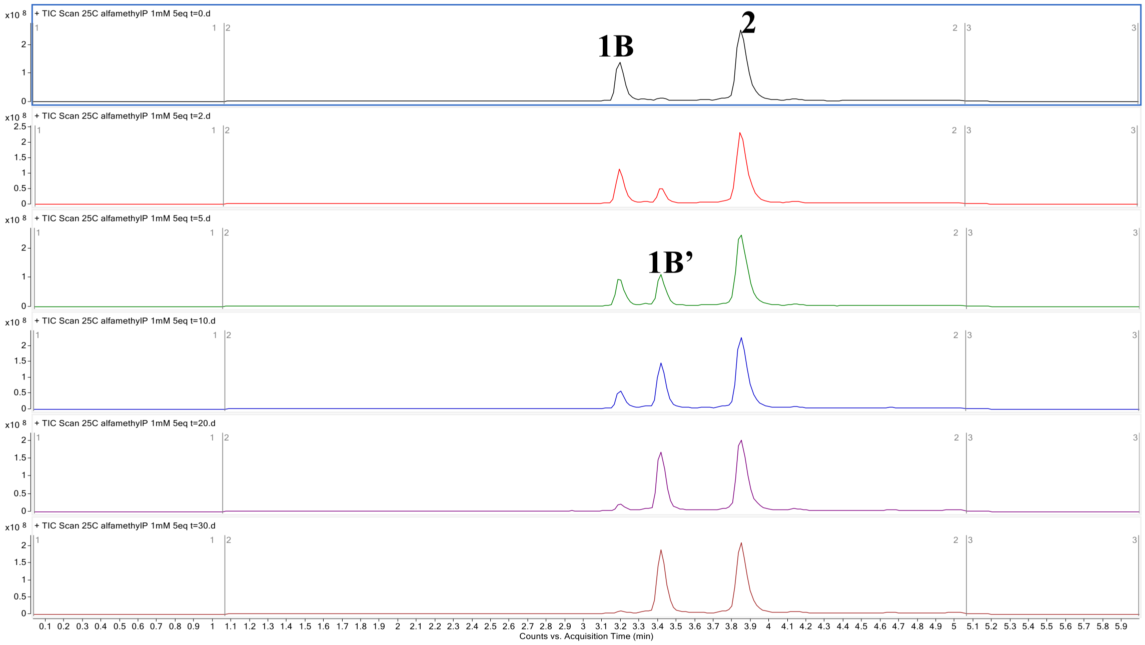

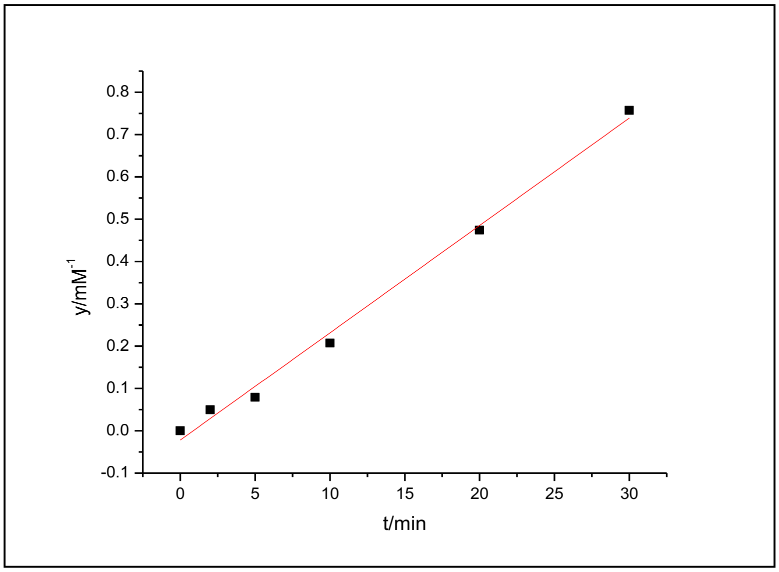

Figure S14. **Kinetics analysis for peptide 1B reacting with probe 2 at 24.4 °C.** Top, LC-MS chromatograms for the reaction between peptide **1B** and probe **2** at different time points (minutes). Reaction conditions: 1 mM **1B**, 5 mM probe **2**, 200 mM phosphate, 20 mM TCEP, pH 8.0, 24.4 °C. Bottom, linear fitting of the kinetics data to second-order rate equation. Slope = 0.025, R^2 = 0.992. **1B'** refers to the product of the arylation reaction between **1B** and probe **2**.

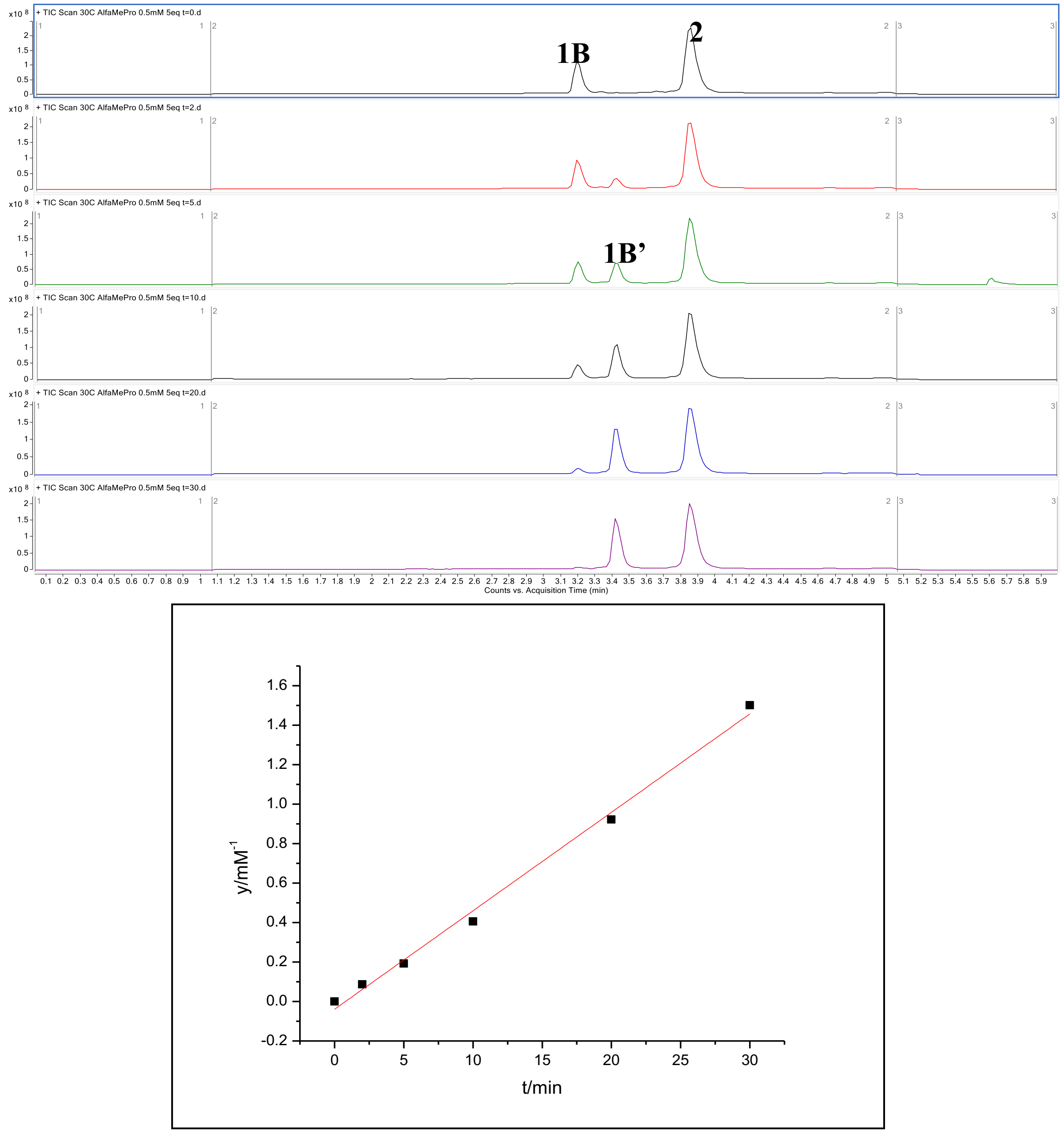

Figure S15. **Kinetics analysis for peptide 1B reacting with probe 2 at 29.0 °C.** Top, LC-MS chromatograms for the reaction between peptide **1B** and probe **2** at different time points (minutes). Reaction conditions: 0.5 mM **1B**, 2.5 mM probe **2**, 200 mM phosphate, 20 mM TCEP, pH 8.0, 29 °C. Bottom, linear fitting of the kinetics data to second-order rate equation. Slope = 0.046, R^2 = 0.993. **1B'** refers to the product of the arylation reaction between **1B** and probe **2**.

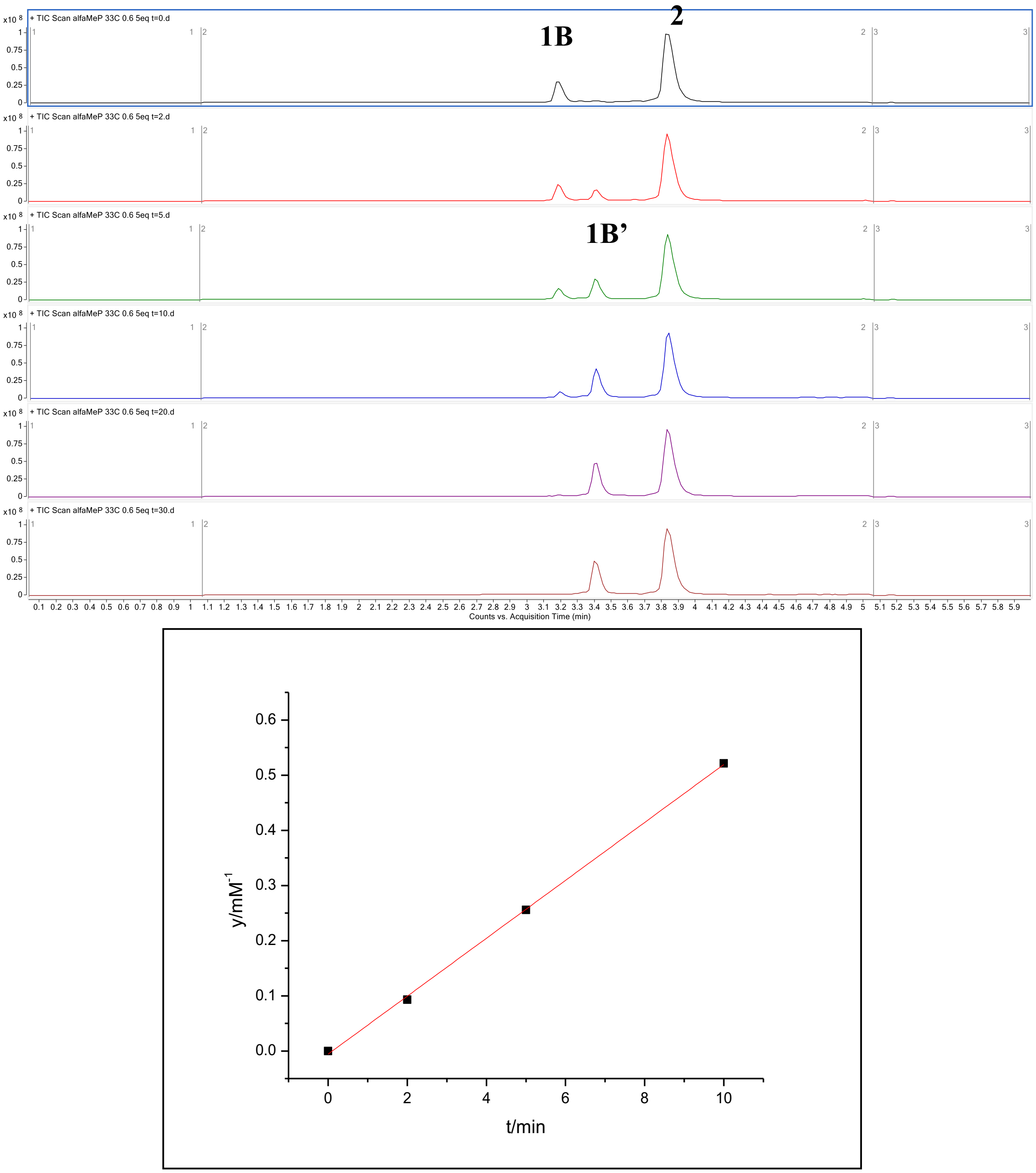

Figure S16. **Kinetics analysis for peptide 1B reacting with probe 2 at 32.7 °C.** Top, LC-MS chromatograms for the reaction between peptide **1B** and probe **2** at different time points (minutes). Reaction conditions: 0.6 mM **1B**, 3 mM probe **2**, 200 mM phosphate, 20 mM TCEP, pH 8.0, 32.7 °C. Bottom, linear fitting of the kinetics data to second-order rate equation. Slope = 0.053, R^2 = 0.999. **1B’** refers to the product of the arylation reaction between **1B** and probe **2**.

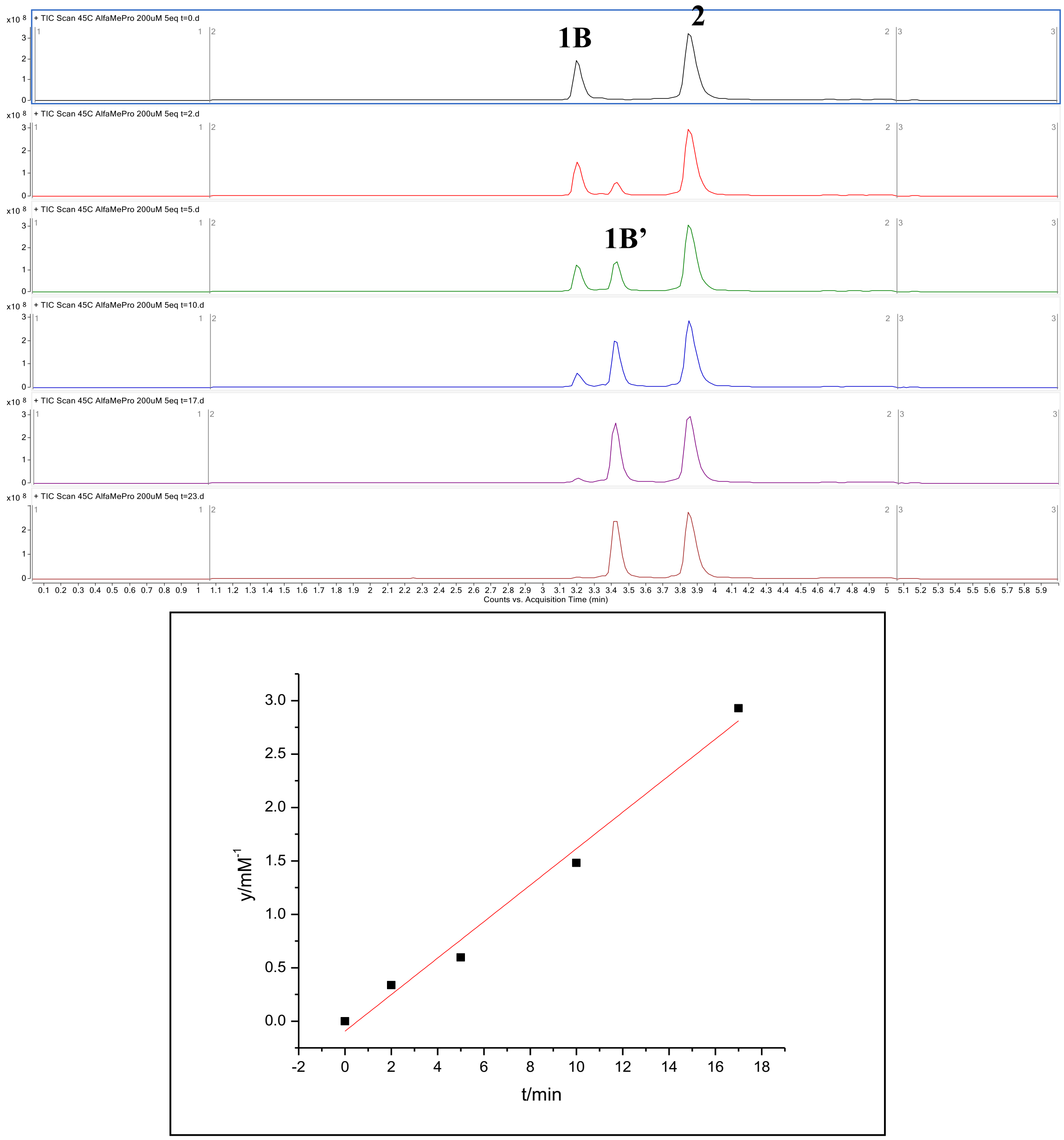

Figure S17. **Kinetics analysis for peptide 1B reacting with probe 2 at 44.8 °C.** Top, LC-MS chromatograms for the reaction between peptide **1B** and probe **2** at different time points (minutes). Reaction conditions: 0.2 mM **1B**, 1 mM probe **2**, 200 mM phosphate, 20 mM TCEP, pH 8.0, 44.8 °C. Bottom, linear fitting of the kinetics data to second-order rate equation. Slope = 0.17, R^2 = 0.98. **1B'** refers to the product of the arylation reaction between **1B** and probe **2**.

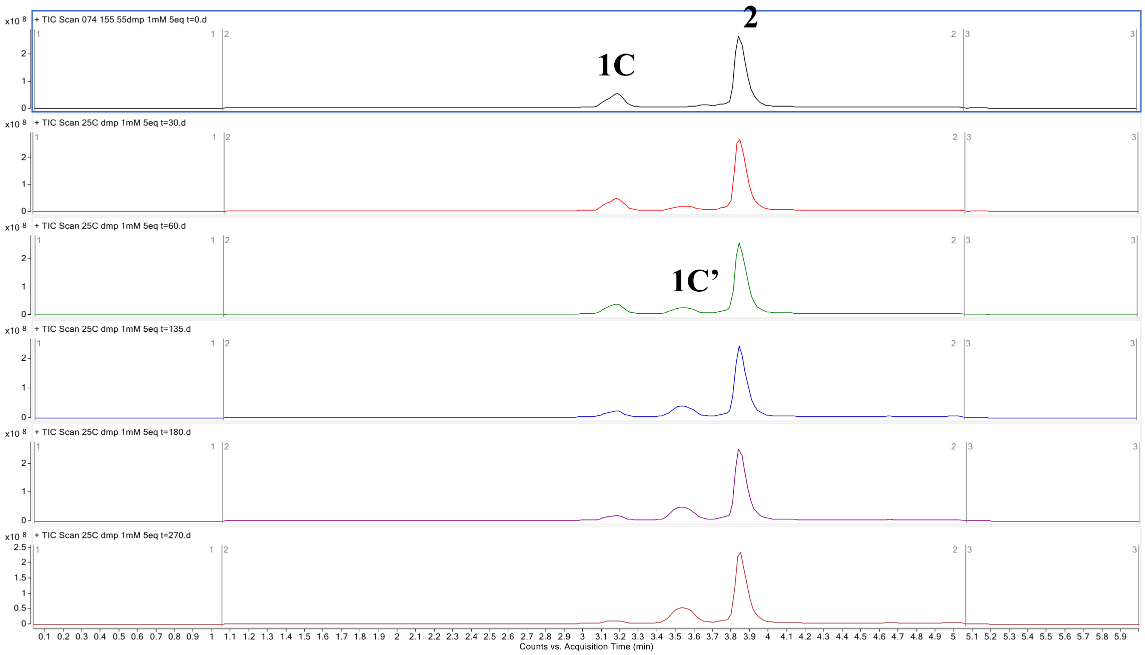

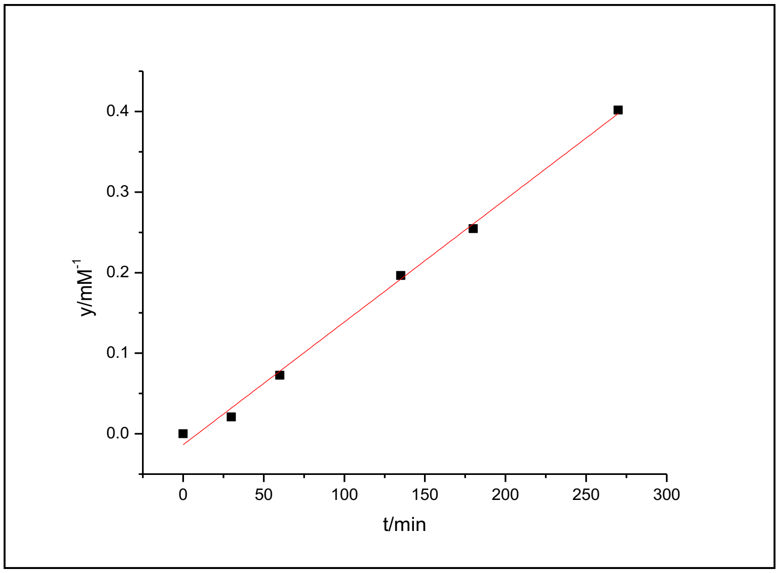

Figure S18. **Kinetics analysis for peptide 1C reacting with probe 2 at 24.4 °C.** Top, LC-MS chromatograms for the reaction between peptide **1C** and probe **2** at different time points (minutes). Reaction conditions: 1 mM **1C**, 5 mM probe **2**, 200 mM phosphate, 20 mM TCEP, pH 8.0, 24.4 °C. Bottom, linear fitting of the kinetics data to second-order rate equation. Slope = 0.0015, R^2 = 0.995. **1C'** refers to the product of the arylation reaction between **1C** and probe **2**.

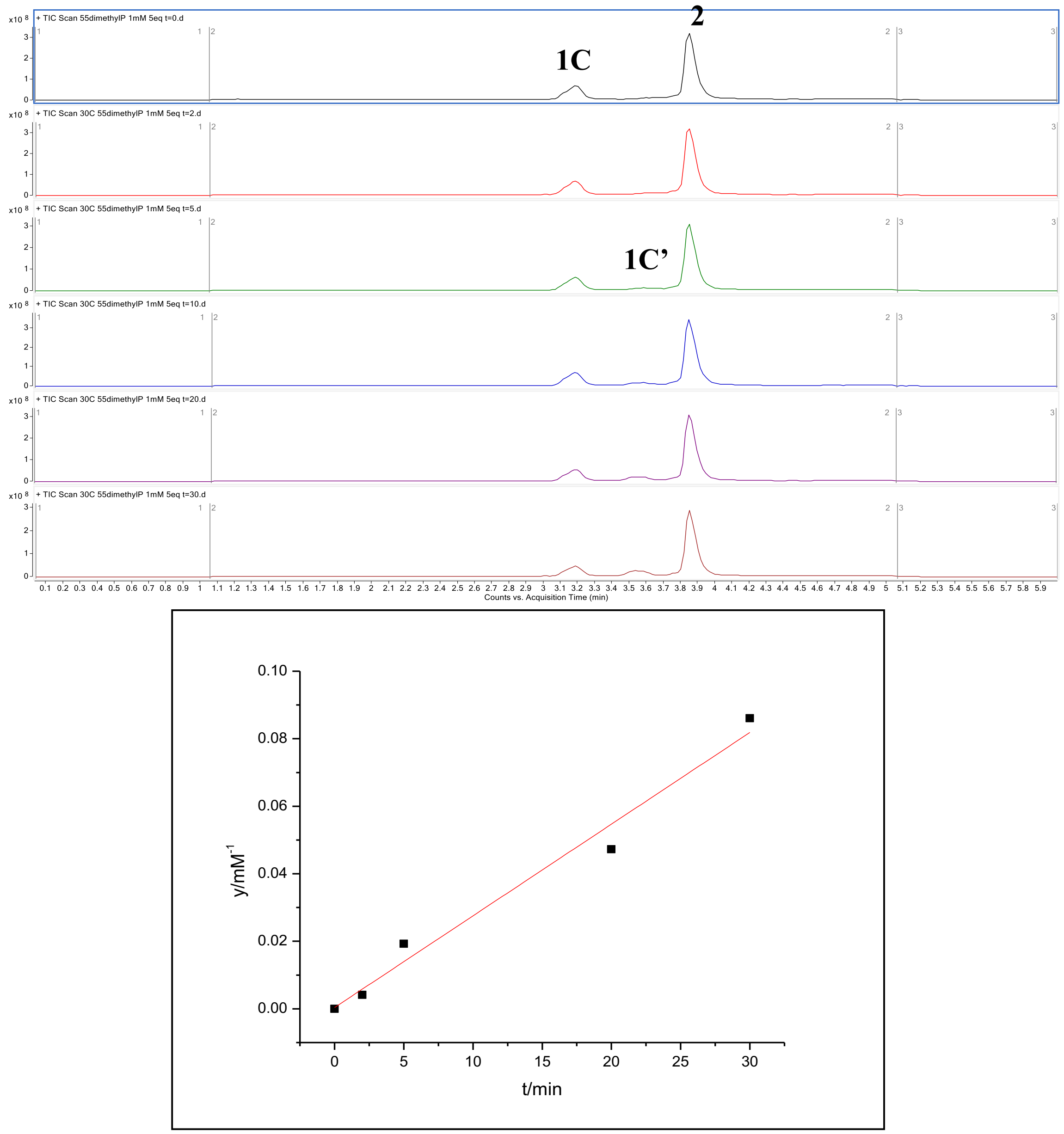

Figure S19. **Kinetics analysis for peptide 1C reacting with probe 2 at 29.0 °C.** Top, LC-MS chromatograms for the reaction between peptide **1C** and probe **2** at different time points (minutes). Reaction conditions: 1 mM **1C**, 5 mM probe **2**, 200 mM phosphate, 20 mM TCEP, pH 8.0, 29 °C. Bottom, linear fitting of the kinetics data to second-order rate equation. Slope = 0.0027, R^2 = 0.97. **1C’** refers to the product of the arylation reaction between **1C** and probe **2**.

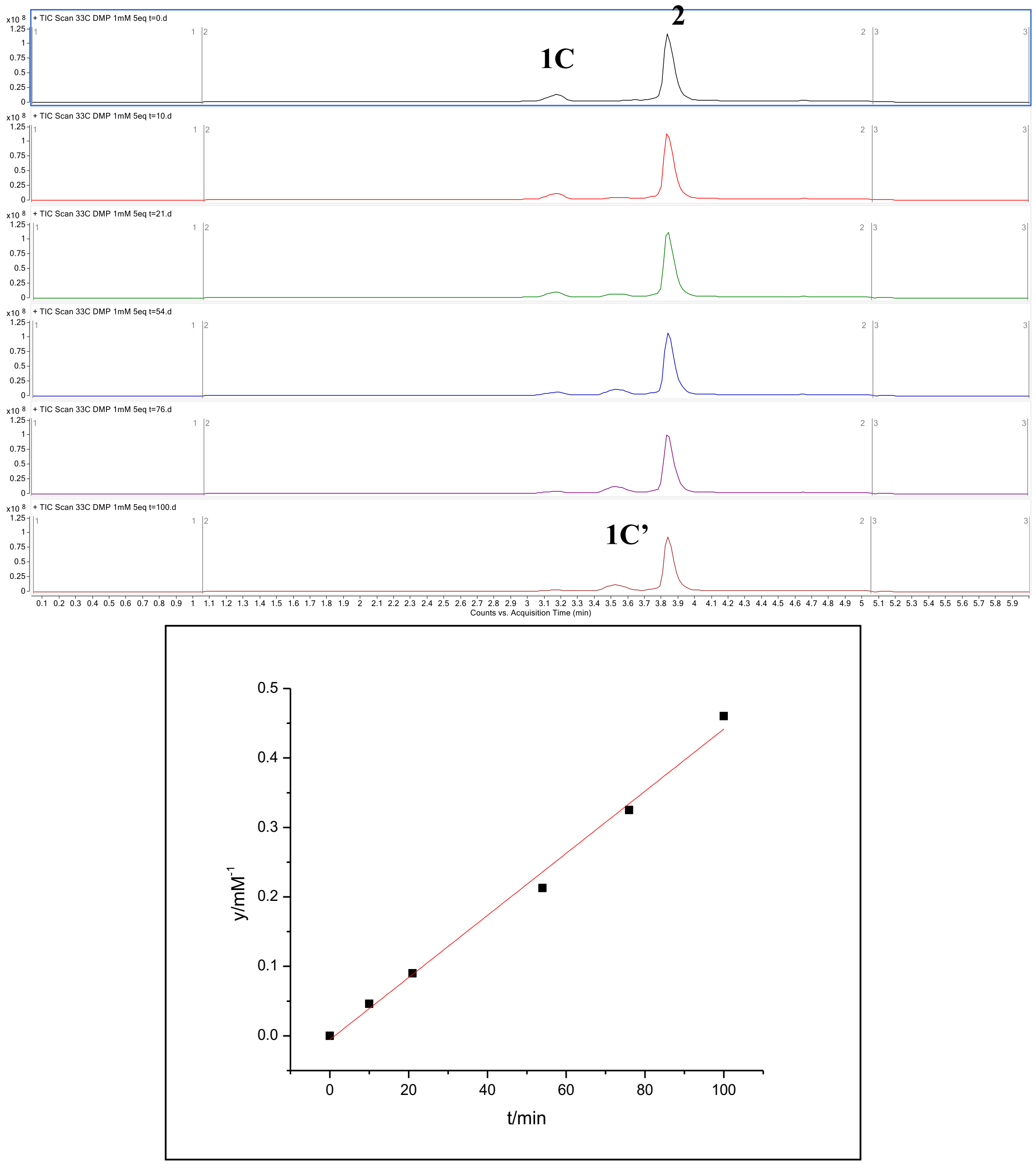

Figure S20. **Kinetics analysis for peptide 1C reacting with probe 2 at 33.0 °C.** Top, LC-MS chromatograms for the reaction between peptide **1C** and probe **2** at different time points (minutes). Reaction conditions: 1 mM **1C**, 5 mM probe **2**, 200 mM phosphate, 20 mM TCEP, pH 8.0, 33 °C. Bottom, linear fitting of the kinetics data to second-order rate equation. Slope = 0.0045, R^2 = 0.993. **1C'** refers to the product of the arylation reaction between **1C** and probe **2**.

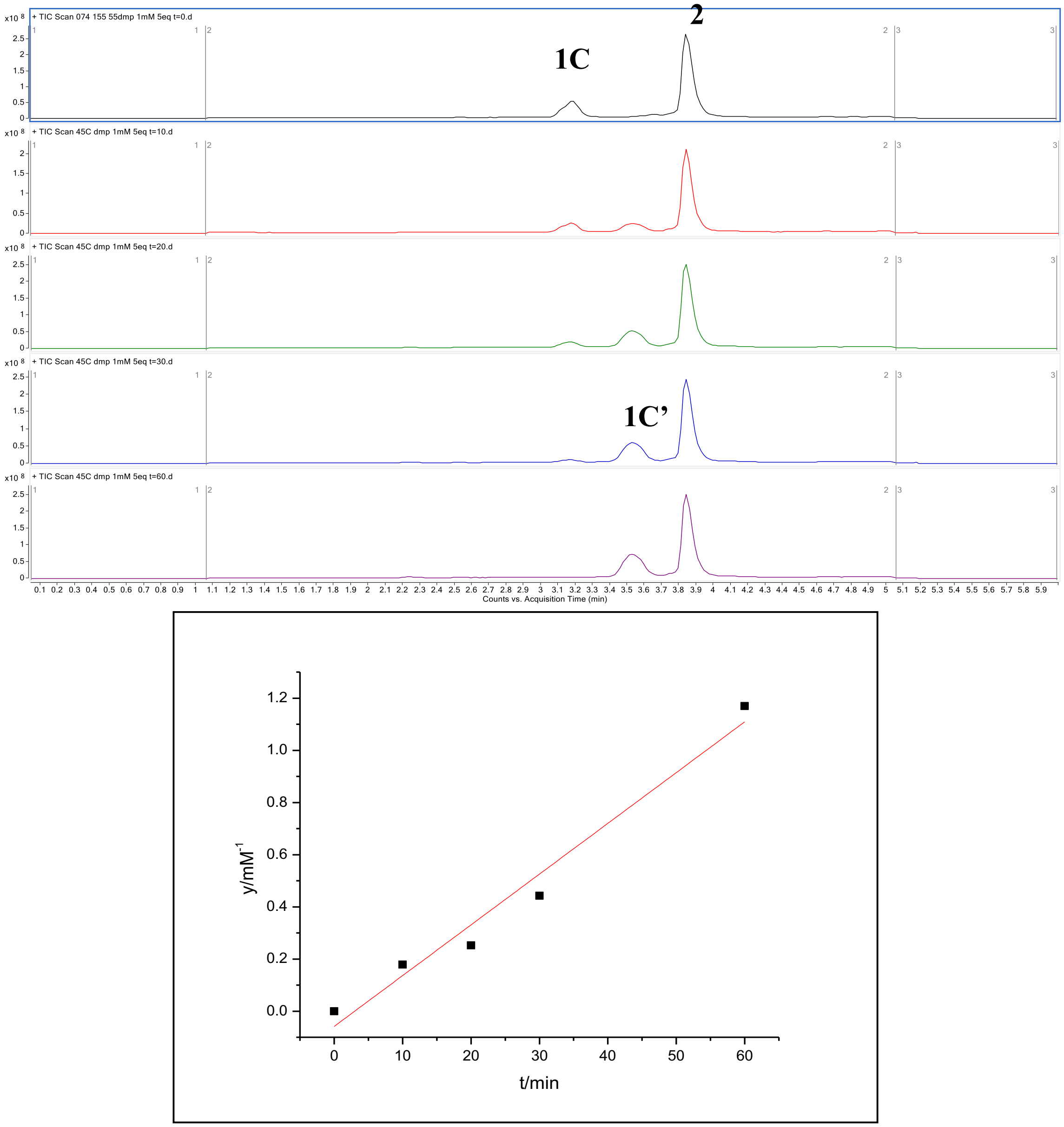

Figure S21. **Kinetics analysis for peptide 1C reacting with probe 2 at 44.8 °C.** Top, LC-MS chromatograms for the reaction between peptide **1C** and probe **2** at different time points (minutes). Reaction conditions: 1 mM **1C**, 5 mM probe **2**, 200 mM phosphate, 20 mM TCEP, pH 8.0, 44.8 °C. Bottom, linear fitting of the kinetics data to second-order rate equation. Slope = 0.0195, R^2 = 0.96. **1C'** refers to the product of the arylation reaction between **1C** and probe **2**.

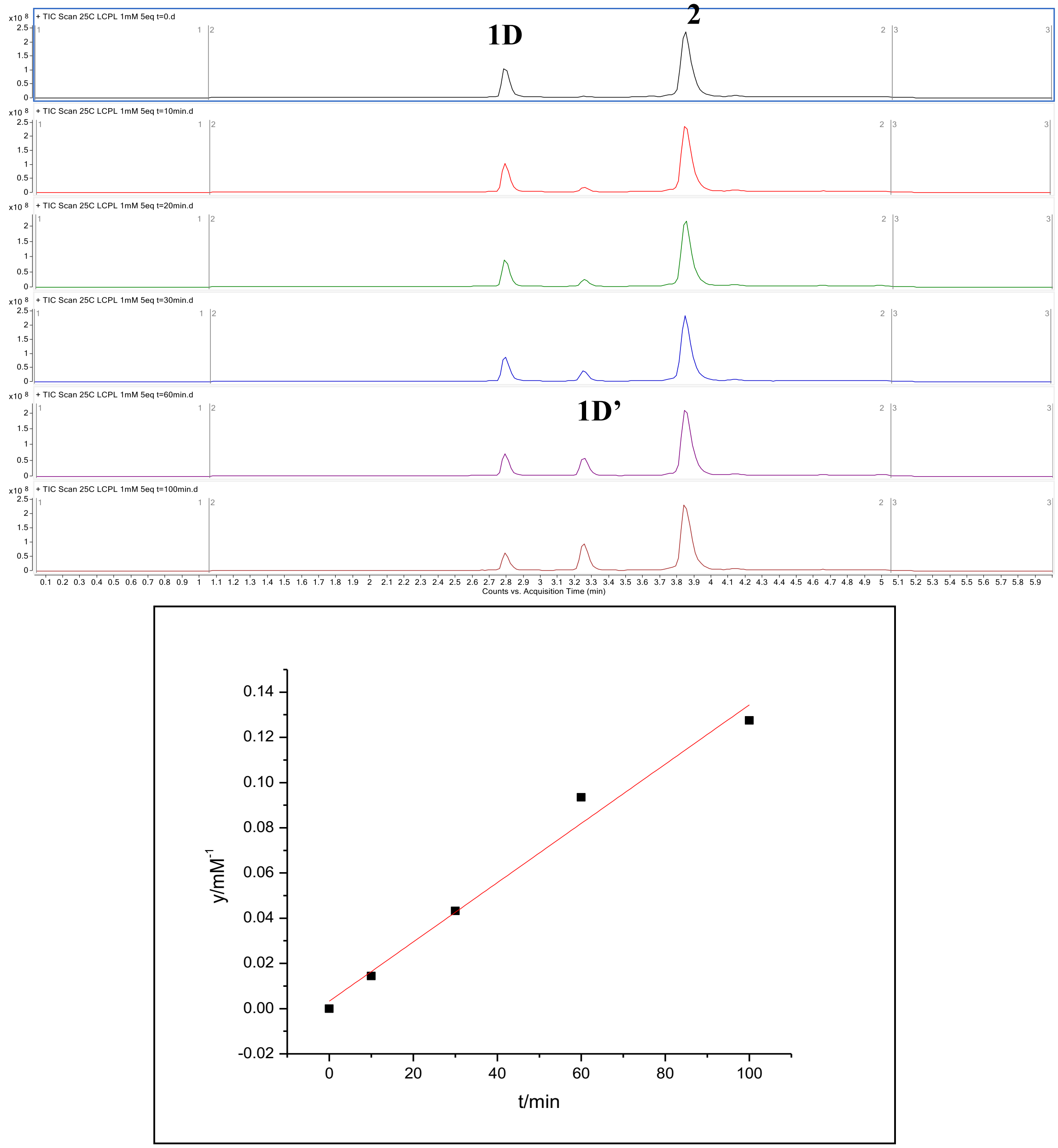

Figure S22. **Kinetics analysis for peptide 1D reacting with probe 2 at 24.4 °C.** Top, LC-MS chromatograms for the reaction between peptide **1D** and probe **2** at different time points (minutes). Reaction conditions: 1 mM **1D**, 5 mM probe **2**, 200 mM phosphate, 20 mM TCEP, pH 8.0, 24.4 °C. Bottom, linear fitting of the kinetics data to second-order rate equation. Slope = 0.0013, R^2 = 0.97. **1D'** refers to the product of the arylation reaction between **1D** and probe **2**.

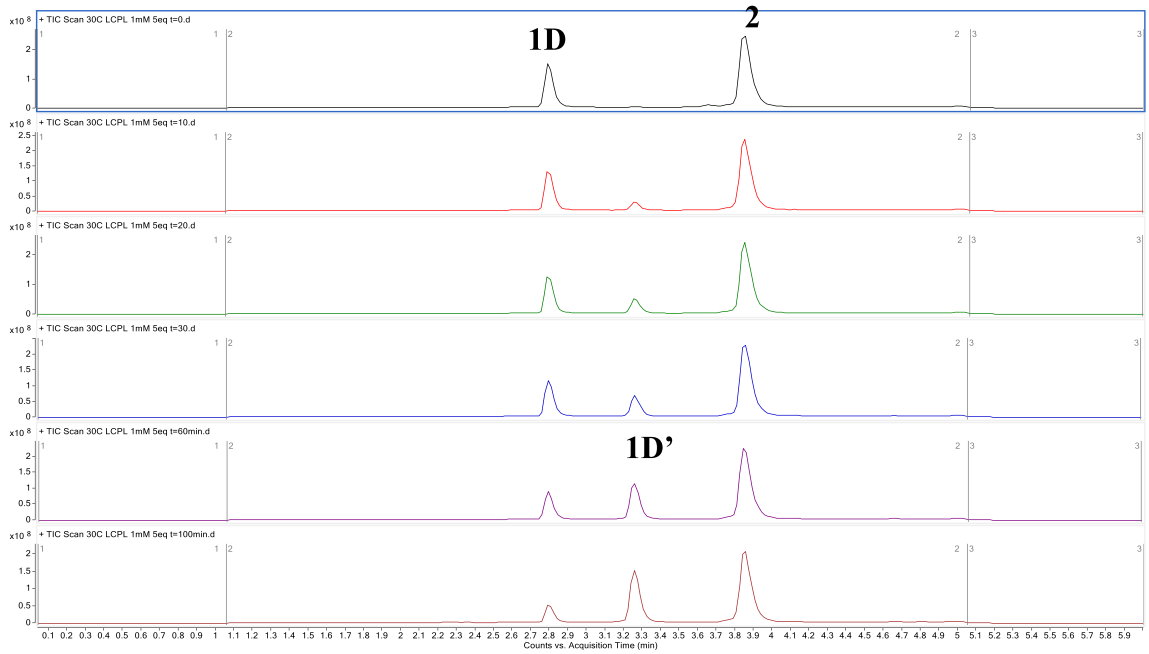

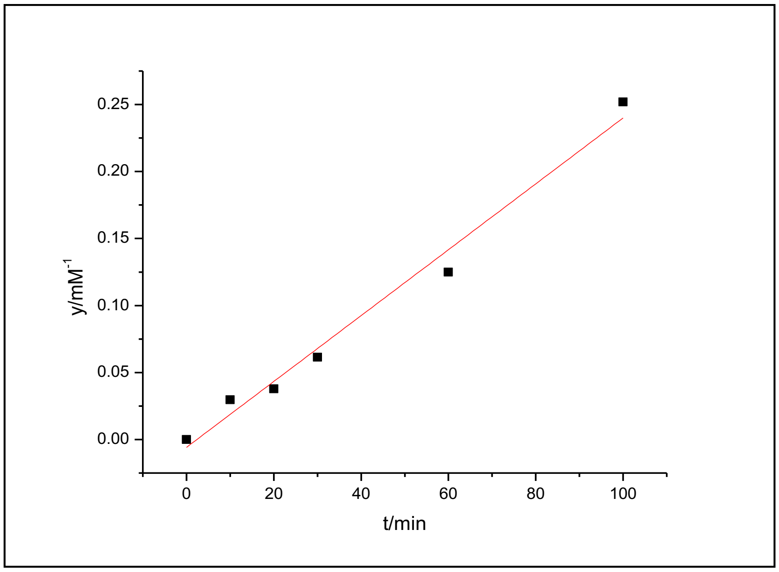

Figure S23. **Kinetics analysis for peptide 1D reacting with probe 2 at 29.0 °C.** Top, LC-MS chromatograms for the reaction between peptide **1D** and probe **2** at different time points (minutes). Reaction conditions: 1 mM **1D**, 5 mM probe **2**, 200 mM phosphate, 20 mM TCEP, pH 8.0, 29 °C. Bottom, linear fitting of the kinetics data to second-order rate equation. Slope = 0.0025, R^2 = 0.98. **1D’** refers to the product of the arylation reaction between **1D** and probe **2**.

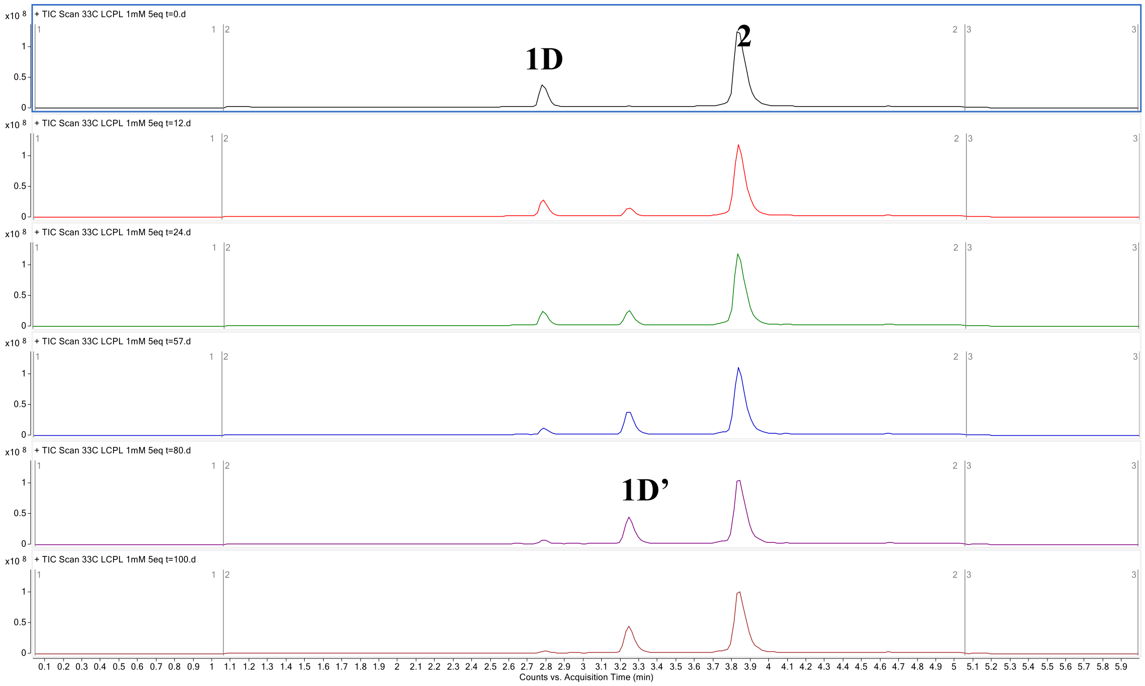

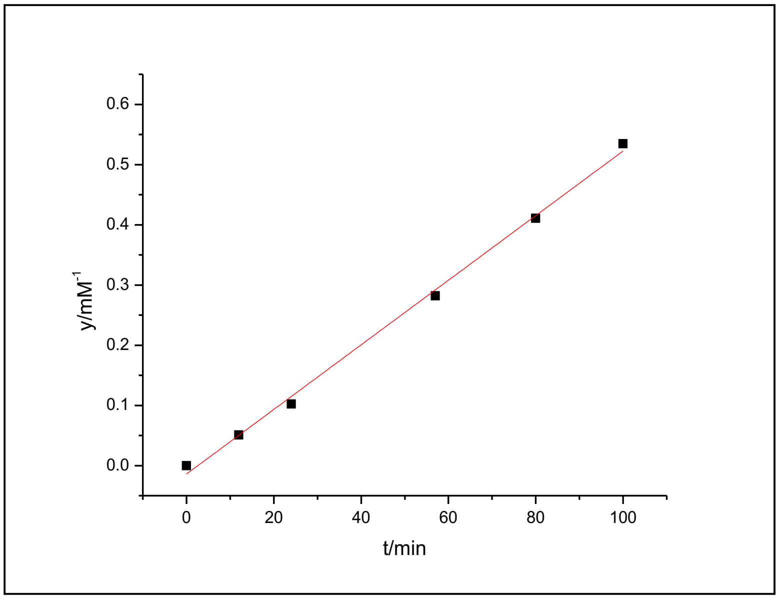

Figure S24. **Kinetics analysis for peptide 1D reacting with probe 2 at 33.3 °C.** Top, LC-MS chromatograms for the reaction between peptide **1D** and probe **2** at different time points (minutes). Reaction conditions: 1 mM **1D**, 5 mM probe **2**, 200 mM phosphate, 20 mM TCEP, pH 8.0, 33.3 °C. Bottom, linear fitting of the kinetics data to second-order rate equation. Slope = 0.0053, R^2 = 0.996. **1D’** refers to the product of the arylation reaction between **1D** and probe **2**.

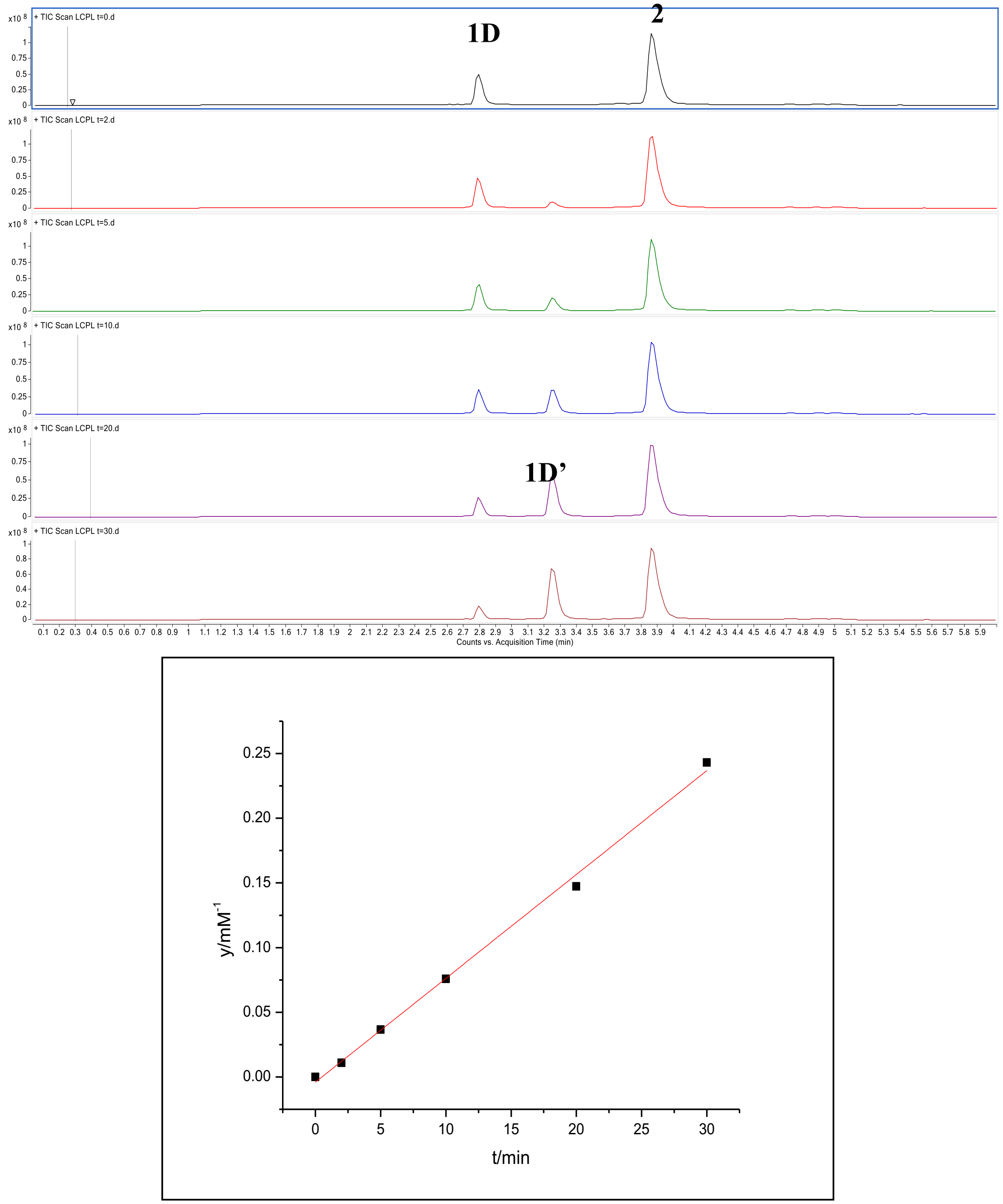

Figure S25. **Kinetics analysis for peptide 1D reacting with probe 2 at 37.7 °C.** Top, LC-MS chromatograms for the reaction between peptide **1D** and probe **2** at different time points (minutes). Reaction conditions: 1 mM **1D**, 5 mM probe **2**, 200 mM phosphate, 20 mM TCEP, pH 8.0, 37.7 °C. Bottom, linear fitting of the kinetics data to second-order rate equation. Slope = 0.0080, R^2 = 0.996. **1D’** refers to the product of the arylation reaction between **1D** and probe **2**.

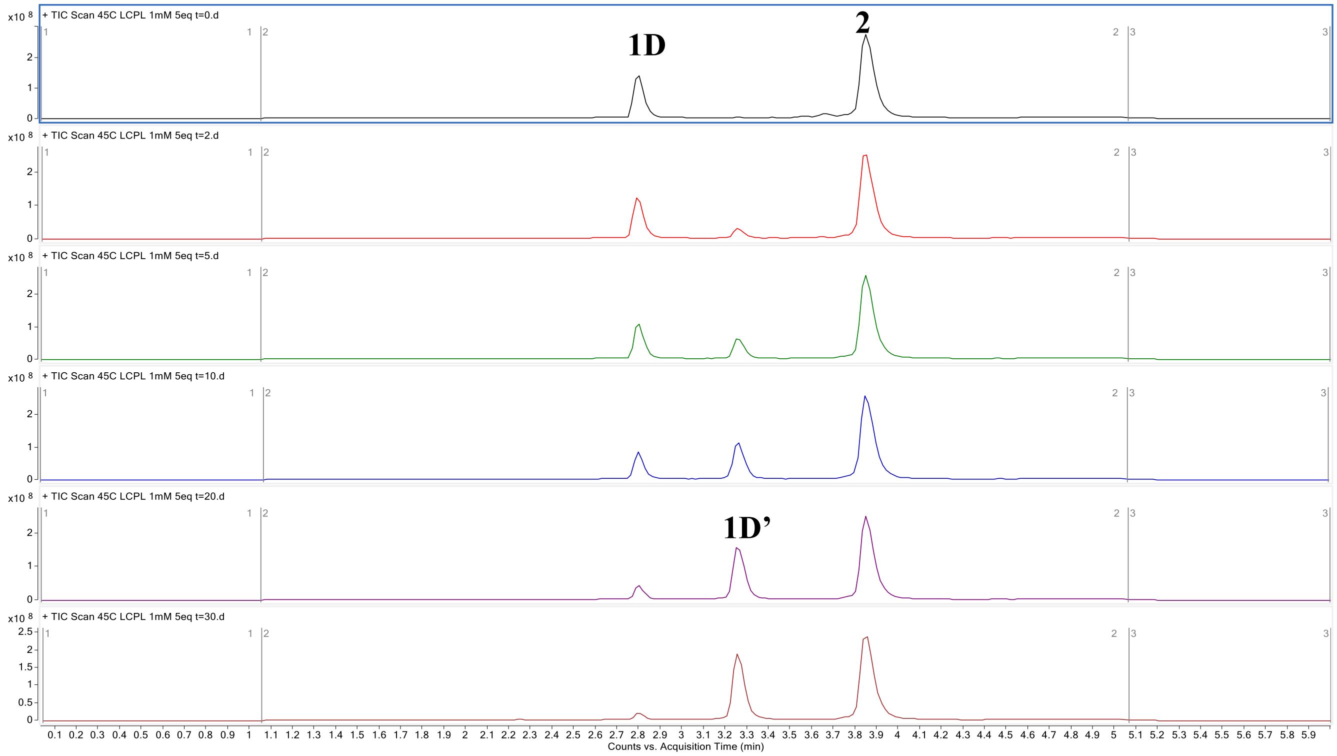

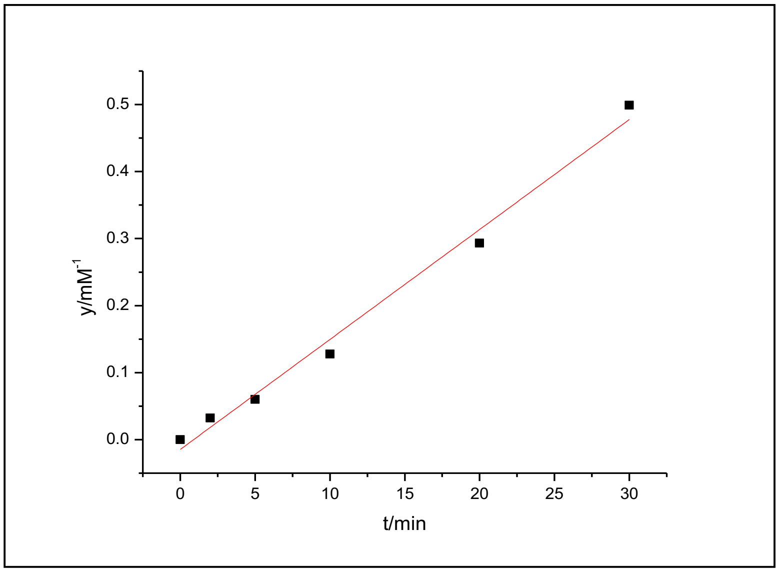

Figure S26. **Kinetics analysis for peptide 1D reacting with probe 2 at 44.8 °C.** Top, LC-MS chromatograms for the reaction between peptide **1D** and probe **2** at different time points (minutes). Reaction conditions: 1 mM **1D**, 5 mM probe **2**, 200 mM phosphate, 20 mM TCEP, pH 8.0, 44.8 °C. Bottom, linear fitting of the kinetics data to second-order rate equation. Slope = 0.0164, R^2 = 0.98. **1D’** refers to the product of the arylation reaction between **1D** and probe **2**.

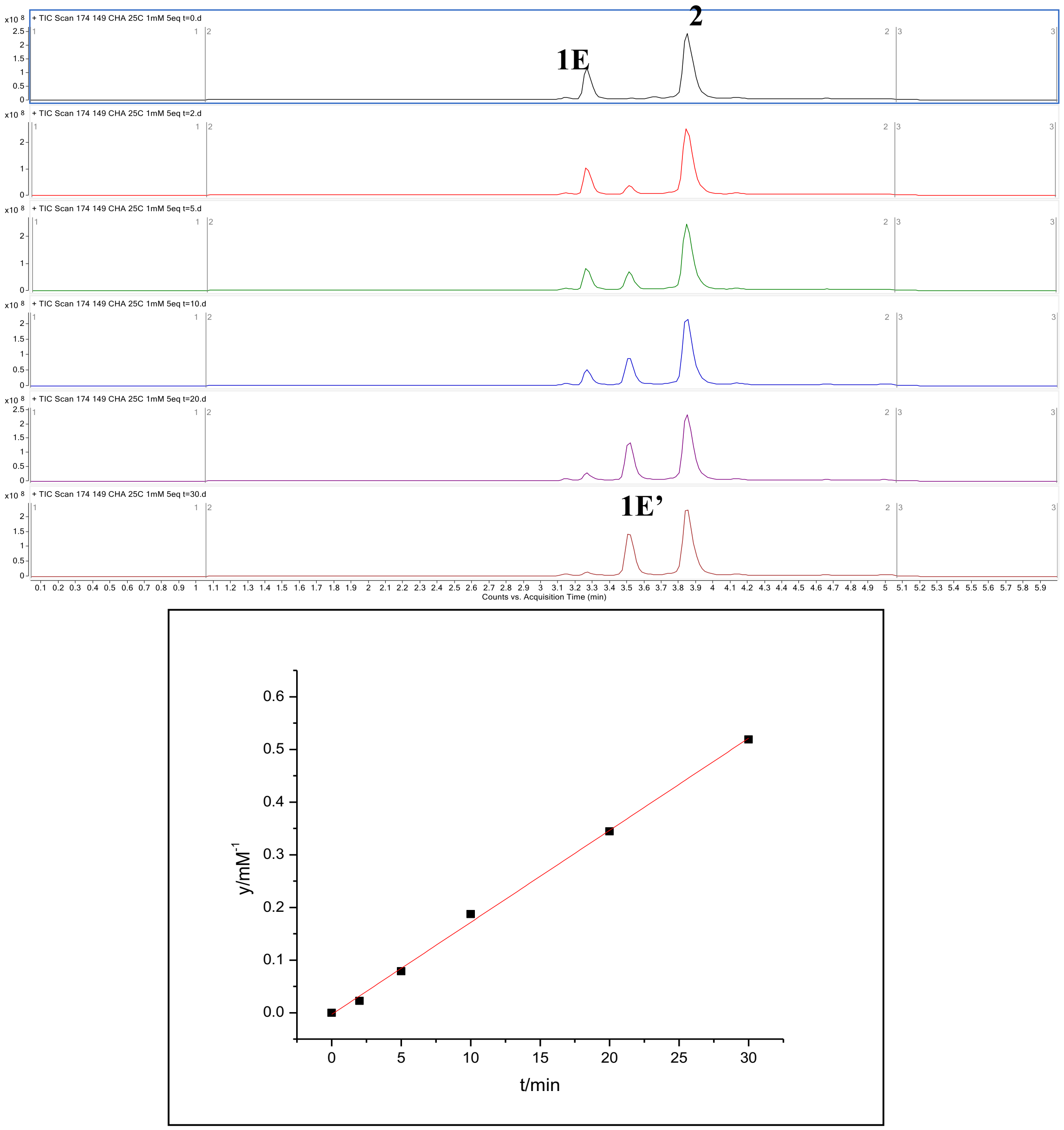

Figure S27. **Kinetics analysis for peptide 1E reacting with probe 2 at 24.4 °C.** Top, LC-MS chromatograms for the reaction between peptide **1E** and probe **2** at different time points (minutes). Reaction conditions: 1 mM **1E**, 5 mM probe **2**, 200 mM phosphate, 20 mM TCEP, pH 8.0, 24.4 °C. Bottom, linear fitting of the kinetics data to second-order rate equation. Slope = 0.0175, R^2 = 0.997. **1E'** refers to the product of the arylation reaction between **1E** and probe **2**.

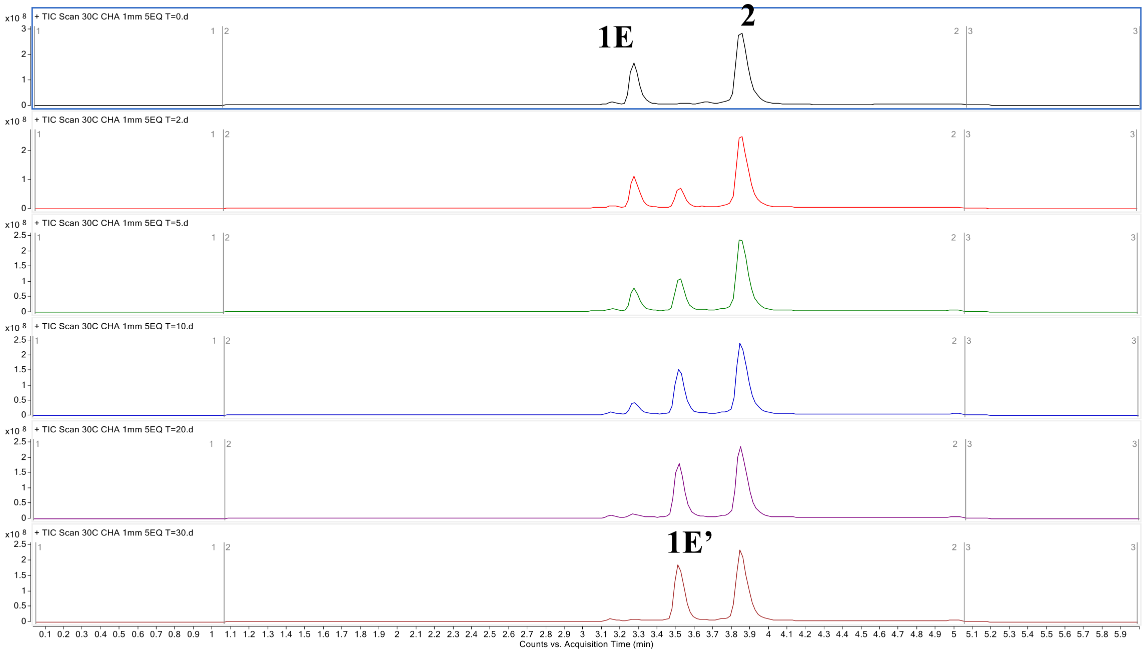

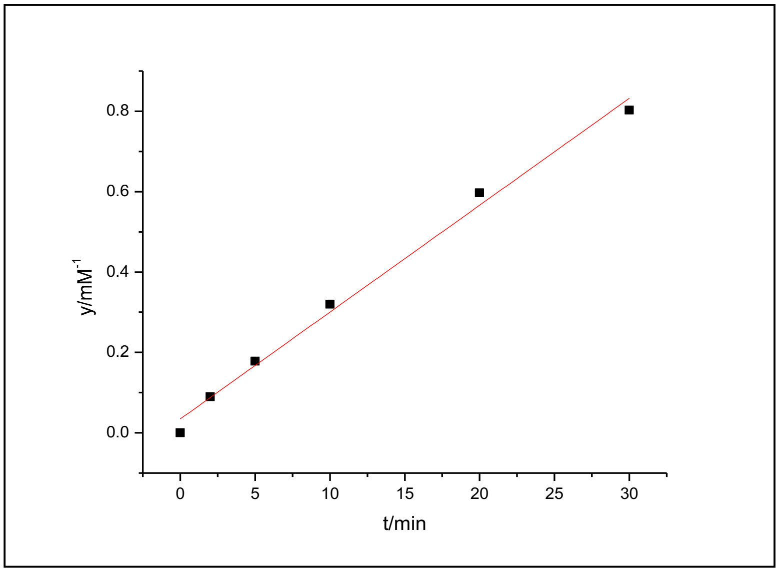

Figure S28. **Kinetics analysis for peptide 1E reacting with probe 2 at 29.0 °C.** Top, LC-MS chromatograms for the reaction between peptide **1E** and probe **2** at different time points (minutes). Reaction conditions: 1 mM **1E**, 5 mM probe **2**, 200 mM phosphate, 20 mM TCEP, pH 8.0, 29 °C. Bottom, linear fitting of the kinetics data to second-order rate equation. Slope = 0.027, R^2 = 0.991. **1E'** refers to the product of the arylation reaction between **1E** and probe **2**.

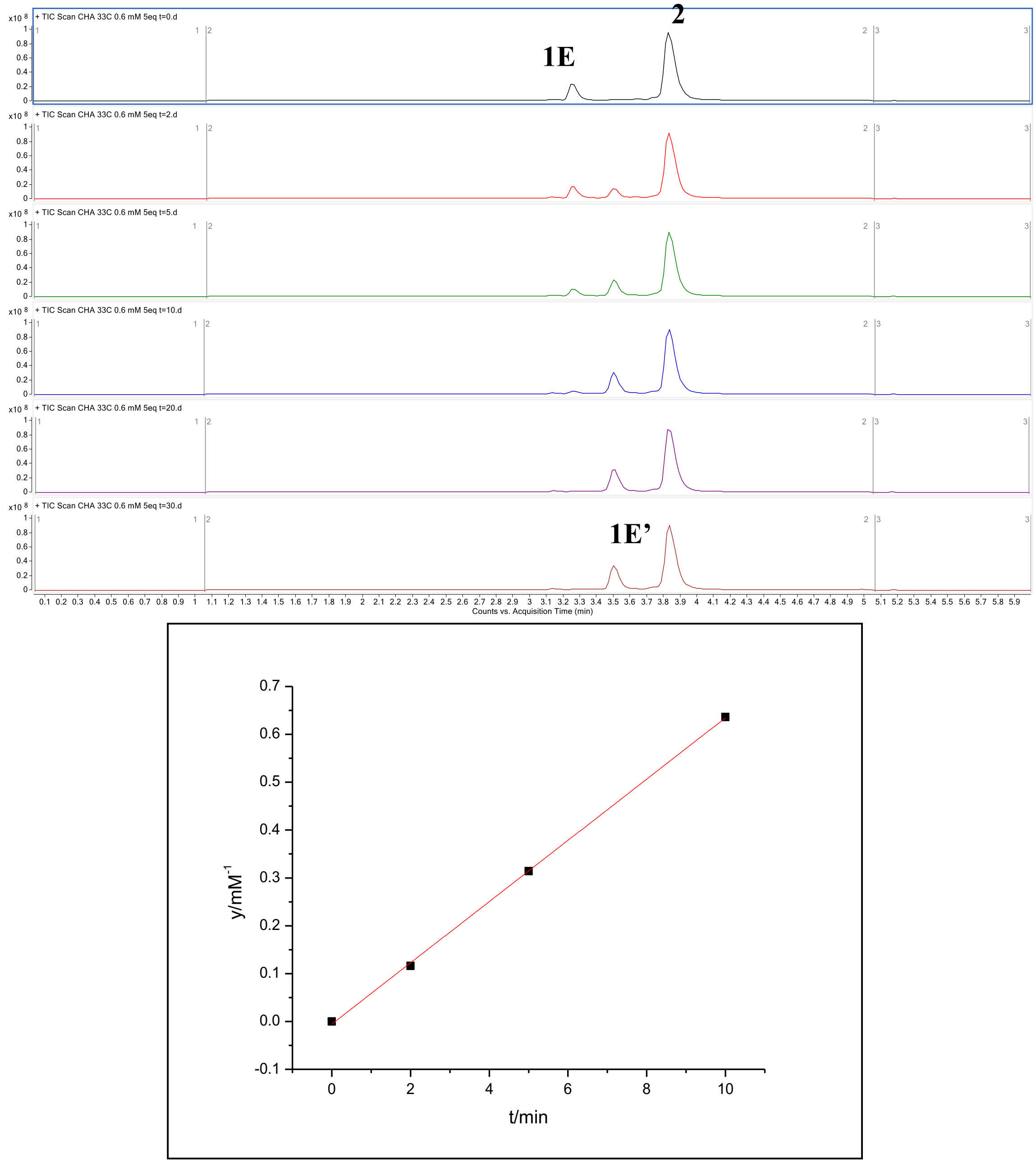

Figure S29. **Kinetics analysis for peptide 1E reacting with probe 2 at 33.0 °C.** Top, LC-MS chromatograms for the reaction between peptide **1E** and probe **2** at different time points (minutes). Reaction conditions: 0.6 mM **1E**, 3 mM probe **2**, 200 mM phosphate, 20 mM TCEP, pH 8.0, 33.0 °C. Bottom, linear fitting of the kinetics data to second-order rate equation. Slope = 0.064, R^2 = 0.999. **1E'** refers to the product of the arylation reaction between **1E** and probe **2**.

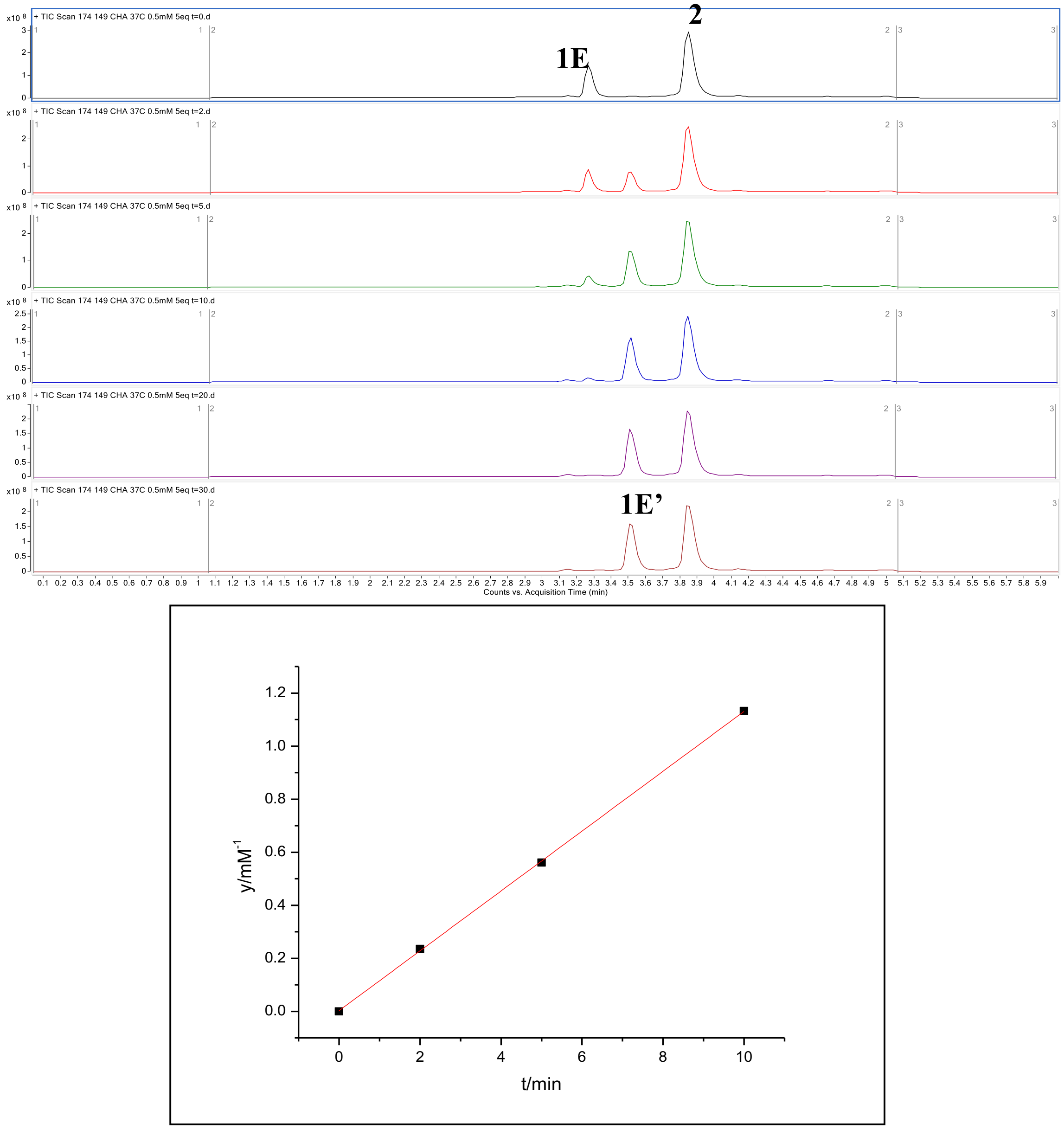

Figure S30. **Kinetics analysis for peptide 1E reacting with probe 2 at 37.7 °C.** Top, LC-MS chromatograms for the reaction between peptide **1E** and probe **2** at different time points (minutes). Reaction conditions: 0.5 mM **1E**, 2.5 mM probe **2**, 200 mM phosphate, 20 mM TCEP, pH 8.0, 37.7 °C. Bottom, linear fitting of the kinetics data to second-order rate equation. Slope = 0.113, R^2 = 0.999. **1E’** refers to the product of the arylation reaction between **1E** and probe **2**.

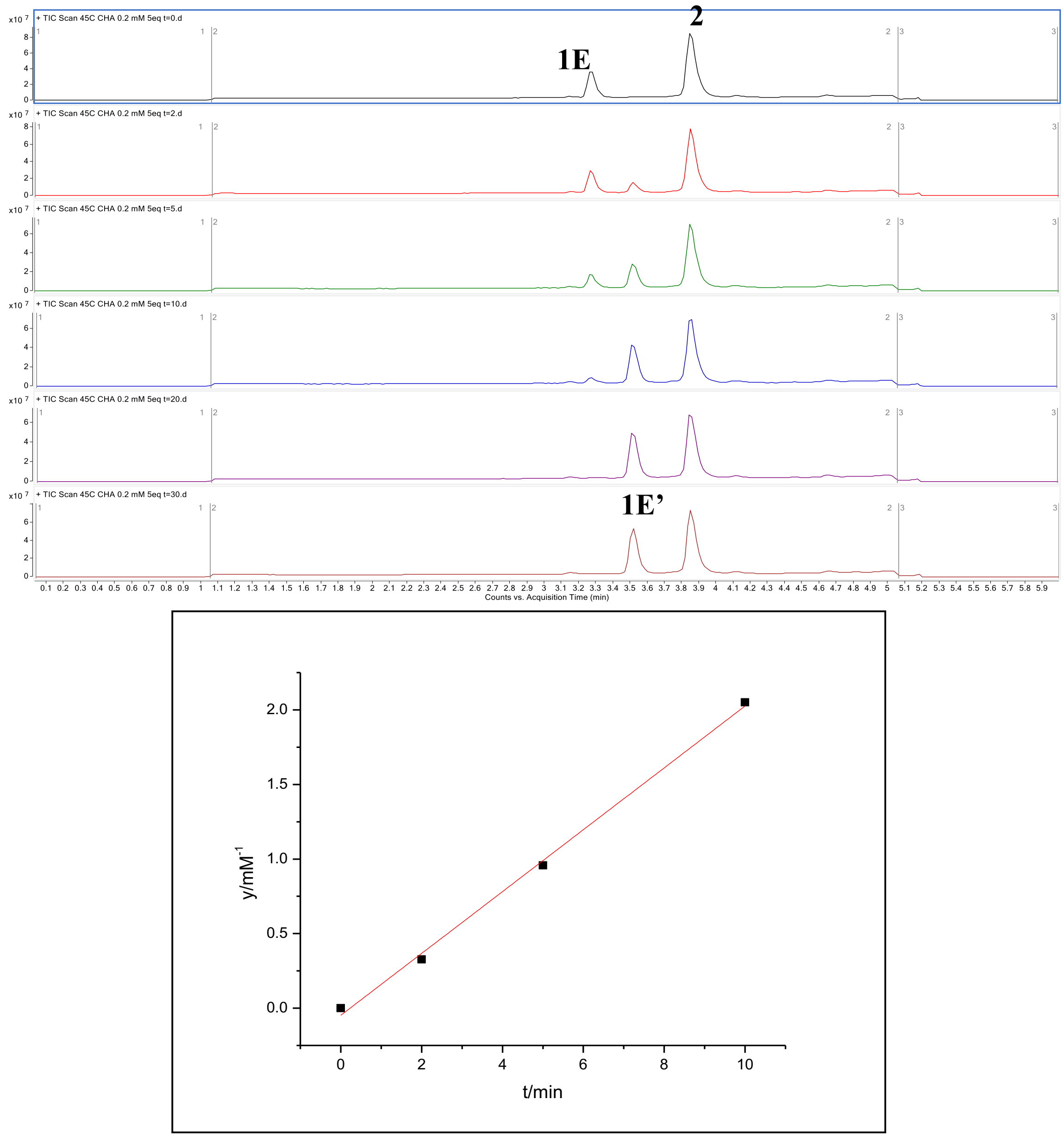

Figure S31. **Kinetics analysis for peptide 1E reacting with probe 2 at 44.8 °C.** Top, LC-MS chromatograms for the reaction between peptide **1E** and probe **2** at different time points (minutes). Reaction conditions: 1 mM **1E**, 5 mM probe **2**, 200 mM phosphate, 20 mM TCEP, pH 8.0, 44.8 °C. Bottom, linear fitting of the kinetics data to second-order rate equation. Slope = 0.21, R^2 = 0.997. **1E'** refers to the product of the arylation reaction between **1E** and probe **2**.

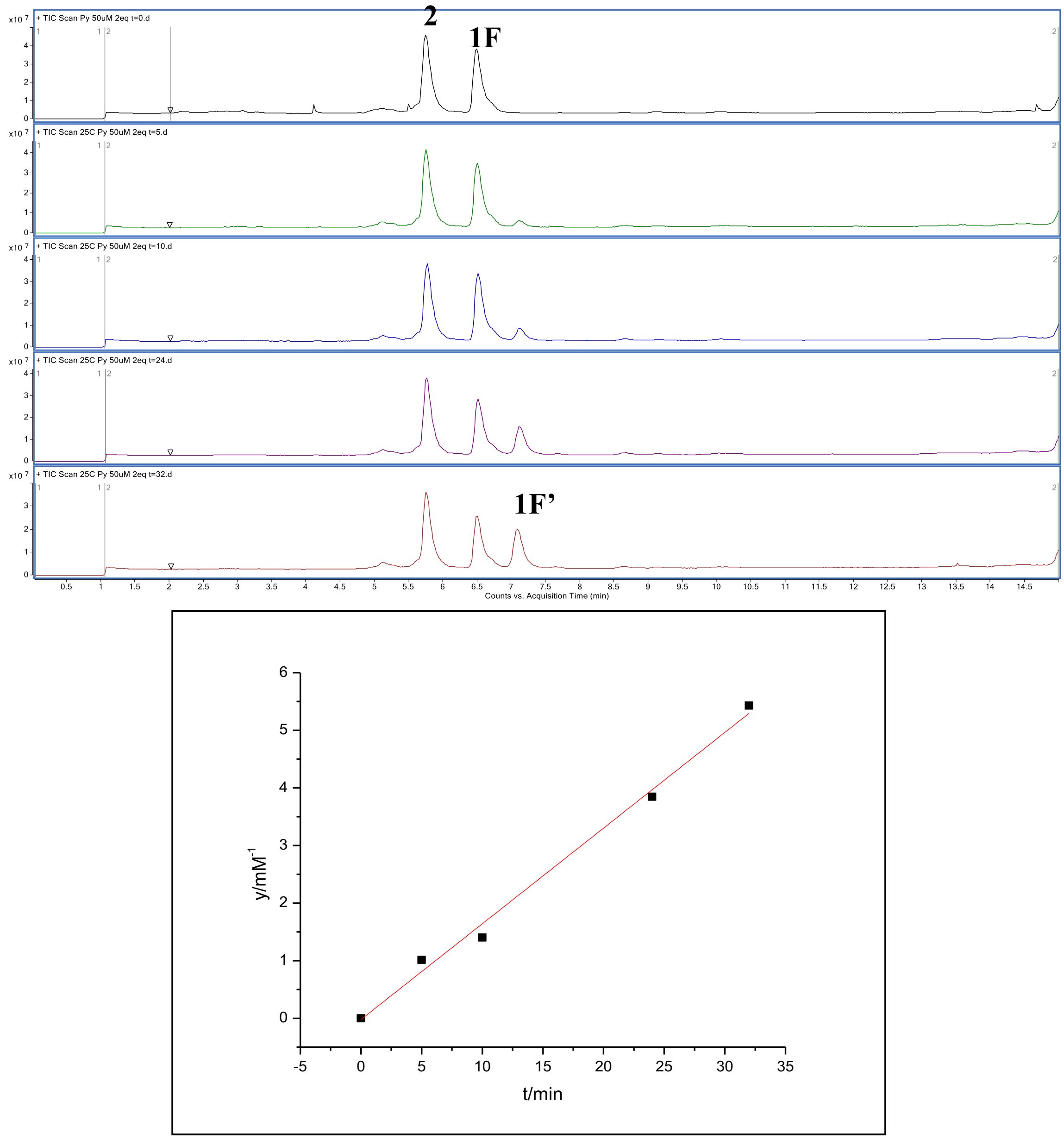

Figure S32. **Kinetics analysis for peptide 1F reacting with probe 2 at 24.4 °C.** Top, LC-MS chromatograms for the reaction between peptide **1F** and probe **2** at different time points (minutes). Reaction conditions: 0.05 mM **1F**, 0.1 mM probe **2**, 200 mM phosphate, 20 mM TCEP, pH 8.0, 24.4 °C. Bottom, linear fitting of the kinetics data to second-order rate equation. Slope = 0.166, R^2 = 0.991. **1F'** refers to the product of the arylation reaction between **1F** and probe **2**.

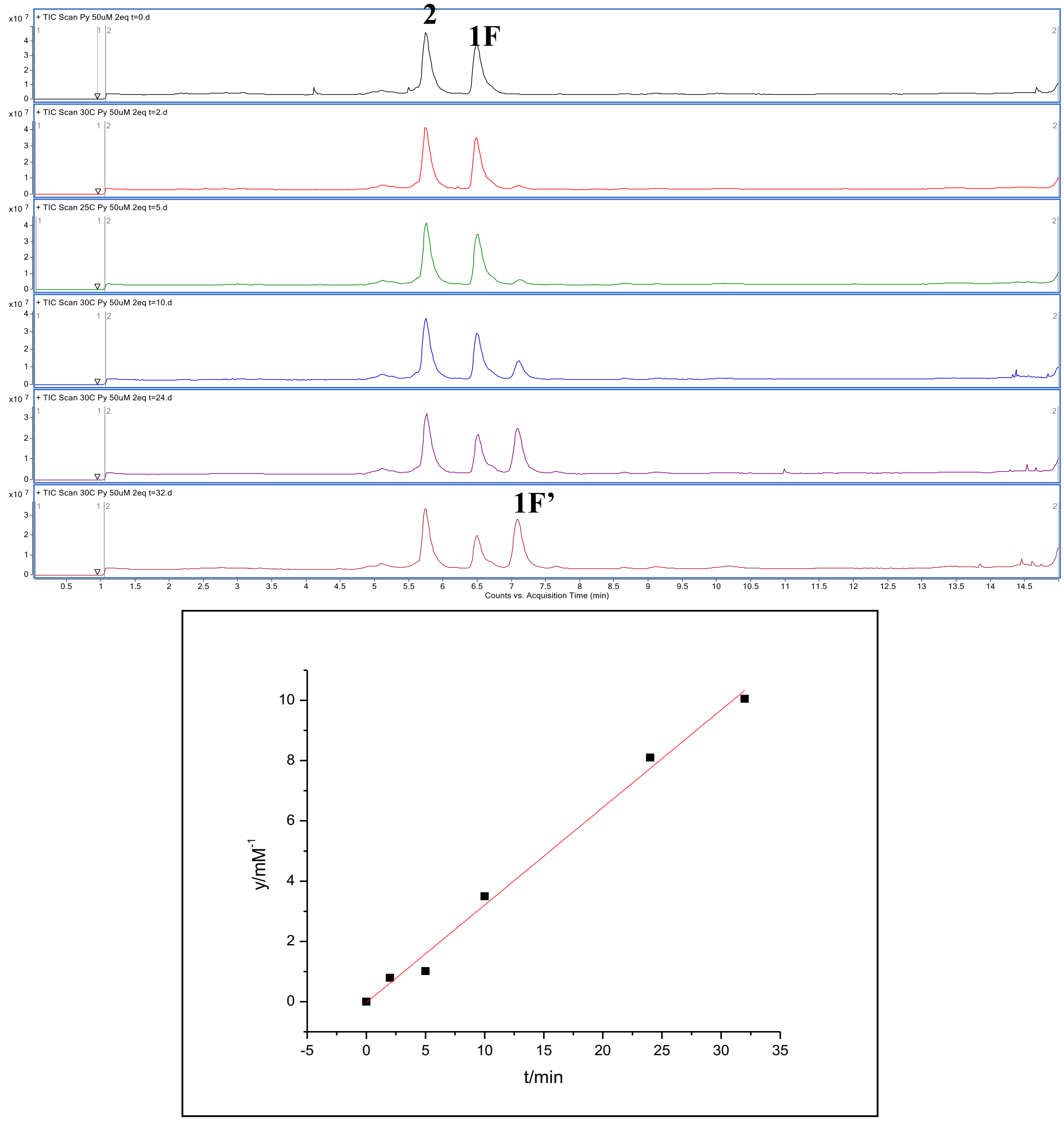

Figure S33. **Kinetics analysis for peptide 1F reacting with probe 2 at 29.0 °C.** Top, LC-MS chromatograms for the reaction between peptide **1F** and probe **2** at different time points (minutes). Reaction conditions: 0.05 mM **1F**, 0.1 mM probe **2**, 200 mM phosphate, 20 mM TCEP, pH 8.0, 29 °C. Bottom, linear fitting of the kinetics data to second-order rate equation. Slope = 0.324, R^2 = 0.99. **1F'** refers to the product of the arylation reaction between **1F** and probe **2**.

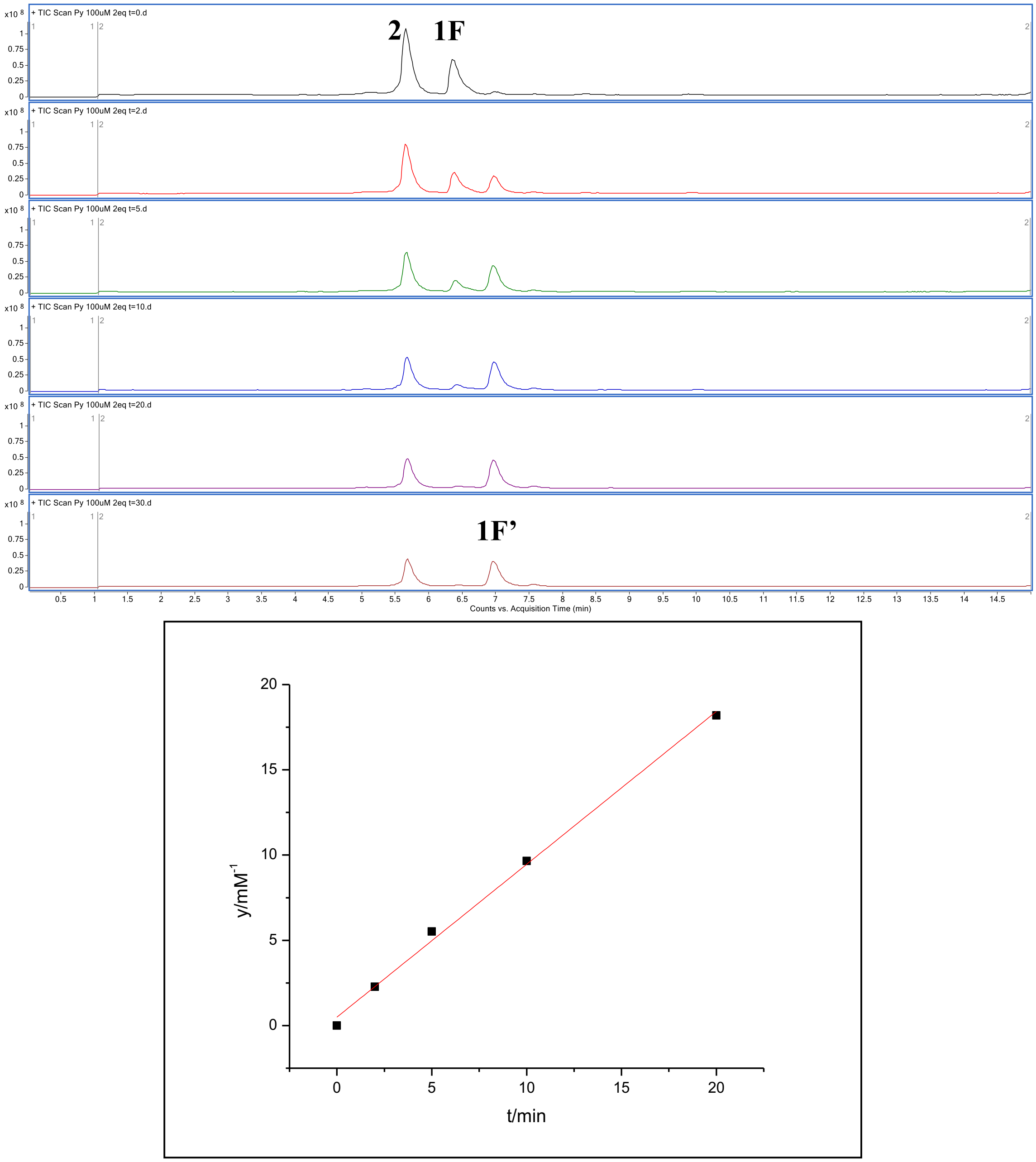

Figure S34. **Kinetics analysis for peptide 1F reacting with probe 2 at 33.3 °C.** Top, LC-MS chromatograms for the reaction between peptide **1F** and probe **2** at different time points (minutes). Reaction conditions: 0.1 mM **1F**, 0.2 mM probe **2**, 200 mM phosphate, 20 mM TCEP, pH 8.0, 33.3 °C. Bottom, linear fitting of the kinetics data to second-order rate equation. Slope = 0.896, R^2 = 0.994. **1F’** refers to the product of the arylation reaction between **1F** and probe **2**.

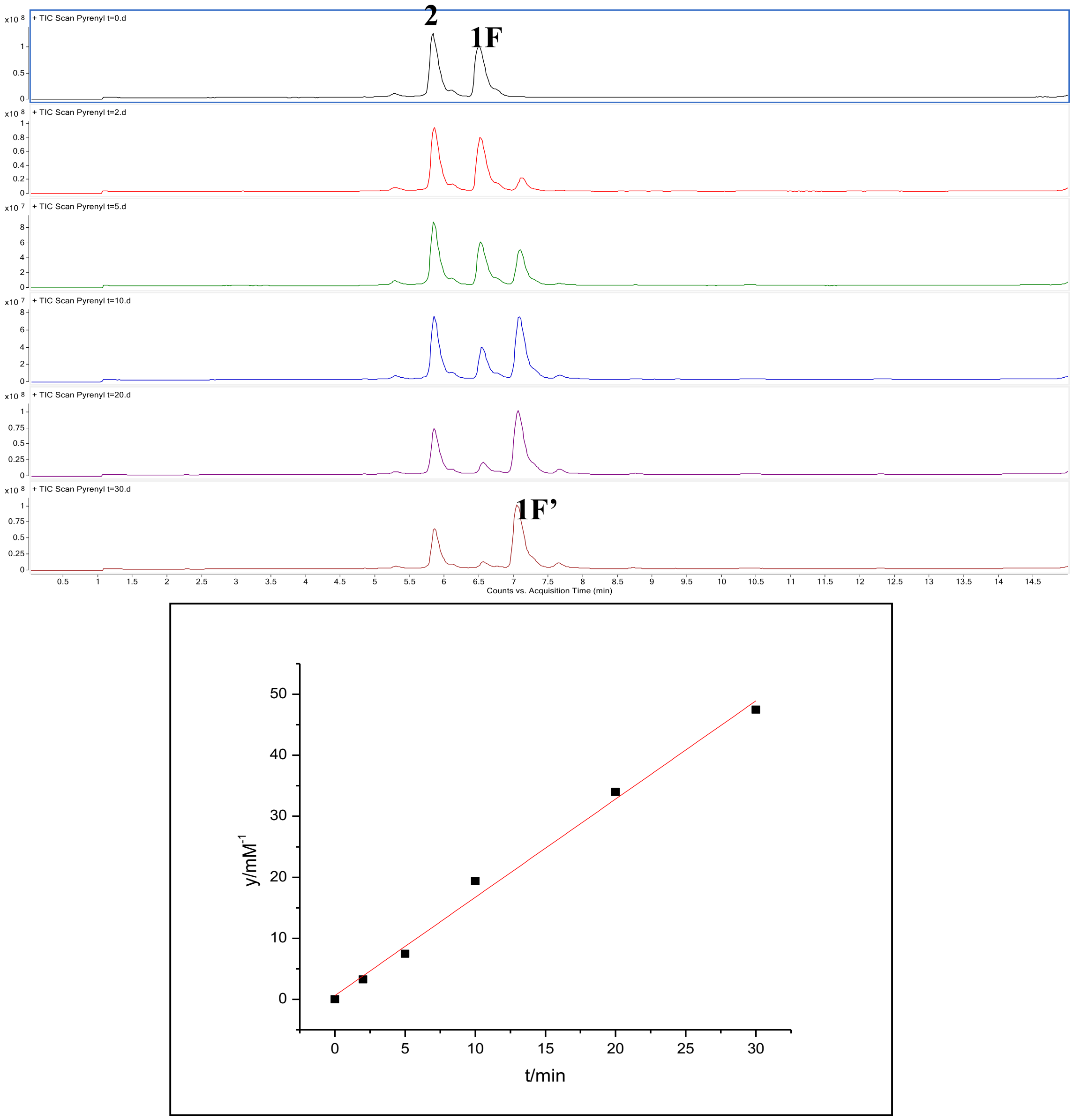

Figure S35. **Kinetics analysis for peptide 1F reacting with probe 2 at 37.7 °C.** Top, LC-MS chromatograms for the reaction between peptide **1F** and probe **2** at different time points (minutes). Reaction conditions: 0.05 mM **1F**, 0.1 mM probe **2**, 200 mM phosphate, 20 mM TCEP, pH 8.0, 37.7 °C. Bottom, linear fitting of the kinetics data to second-order rate equation. Slope = 1.61, R^2 = 0.991. **1F'** refers to the product of the arylation reaction between **1F** and probe **2**.

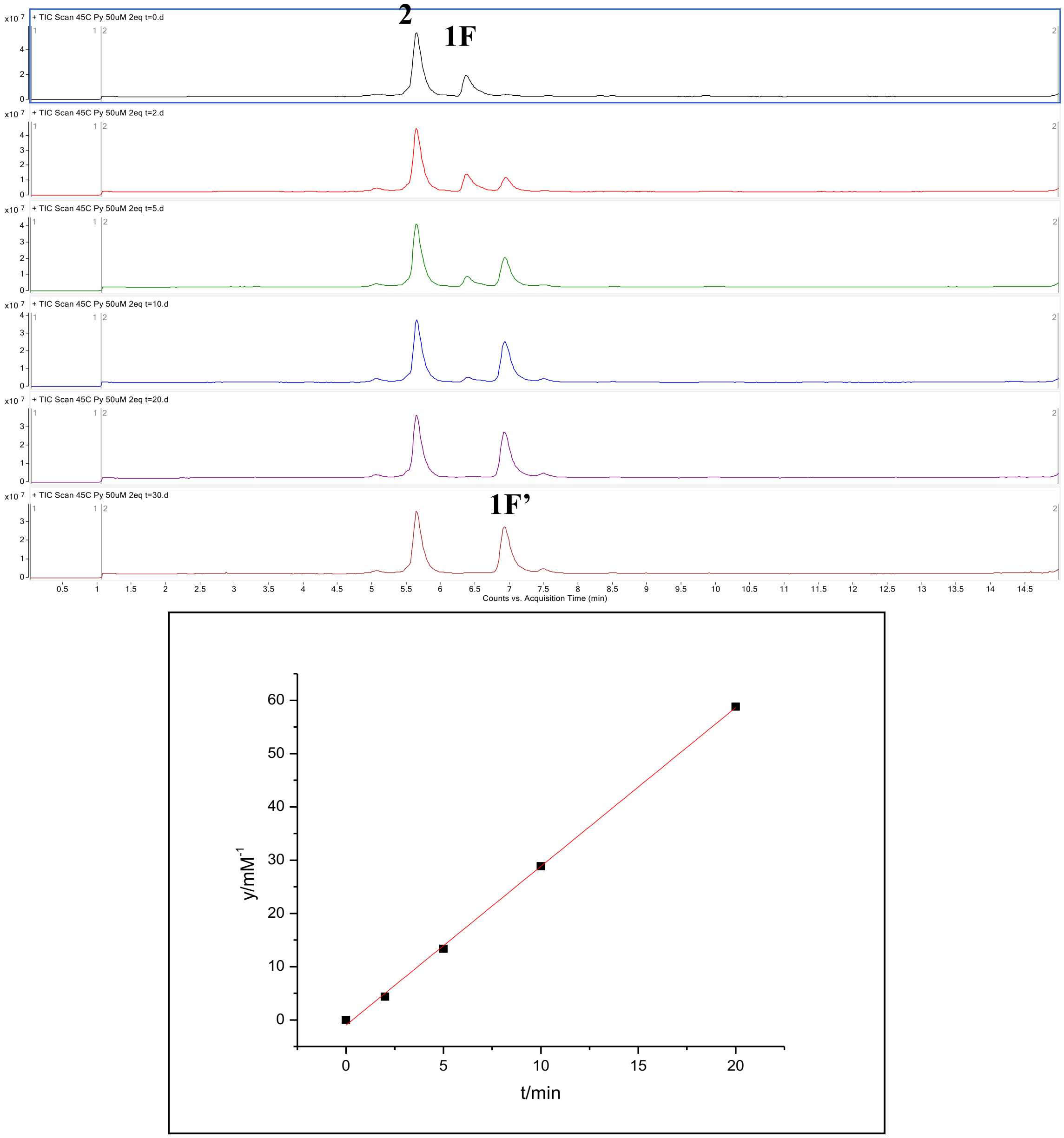

Figure S36. **Kinetics analysis for peptide 1F reacting with probe 2 at 44.3 °C.** Top, LC-MS chromatograms for the reaction between peptide **1F** and probe **2** at different time points (minutes). Reaction conditions: 0.05 mM **1F**, 0.1 mM probe **2**, 200 mM phosphate, 20 mM TCEP, pH 8.0, 44.3 °C. Bottom, linear fitting of the kinetics data to second-order rate equation. Slope = 3.03, R^2 = 0.999. **1F'** refers to the product of the arylation reaction between **1F** and probe **2**.

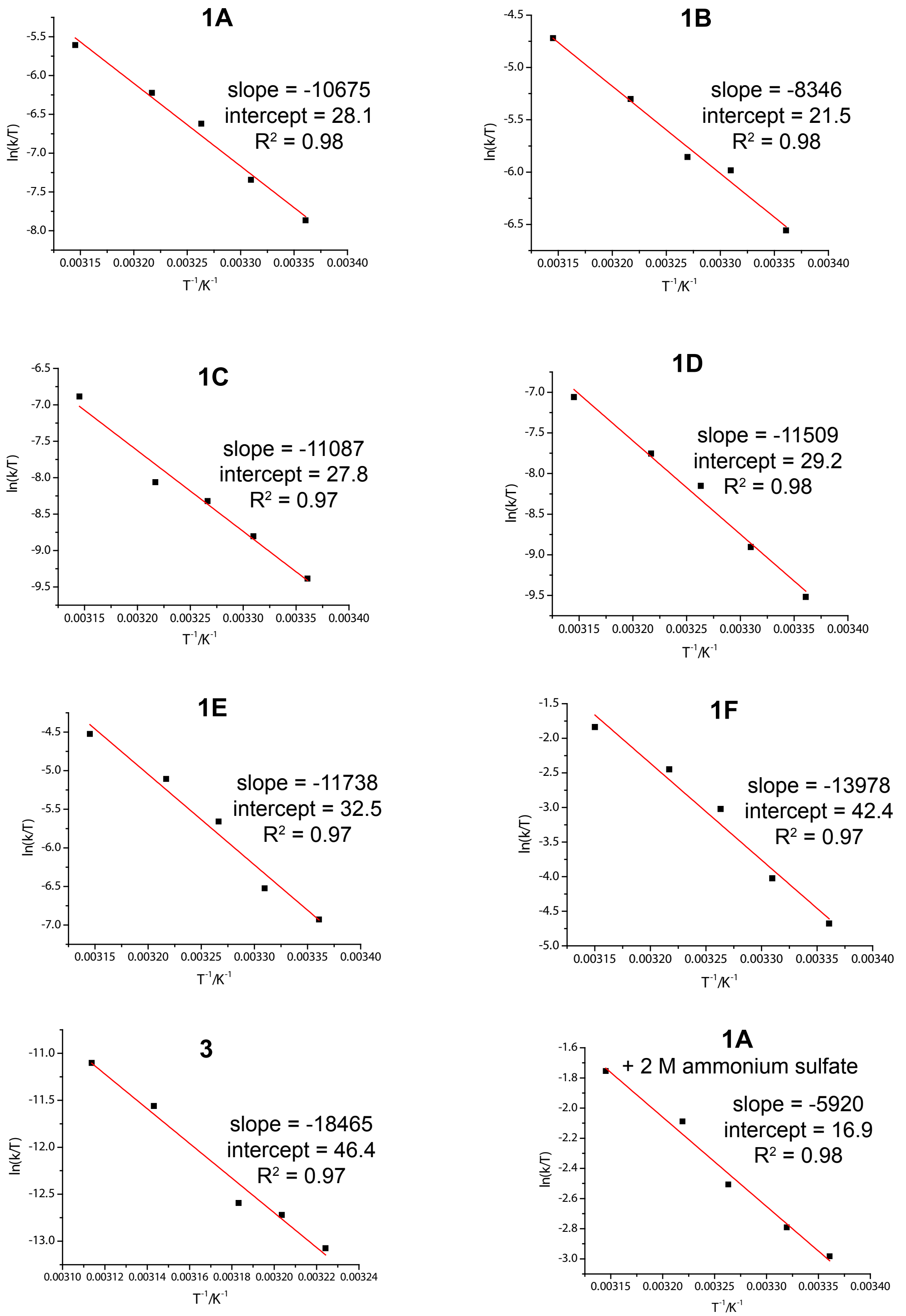

Figure S37. Fitting of the reaction rate constants at different temperature with Eyring equation for the calculation of reaction thermodynamic parameters.

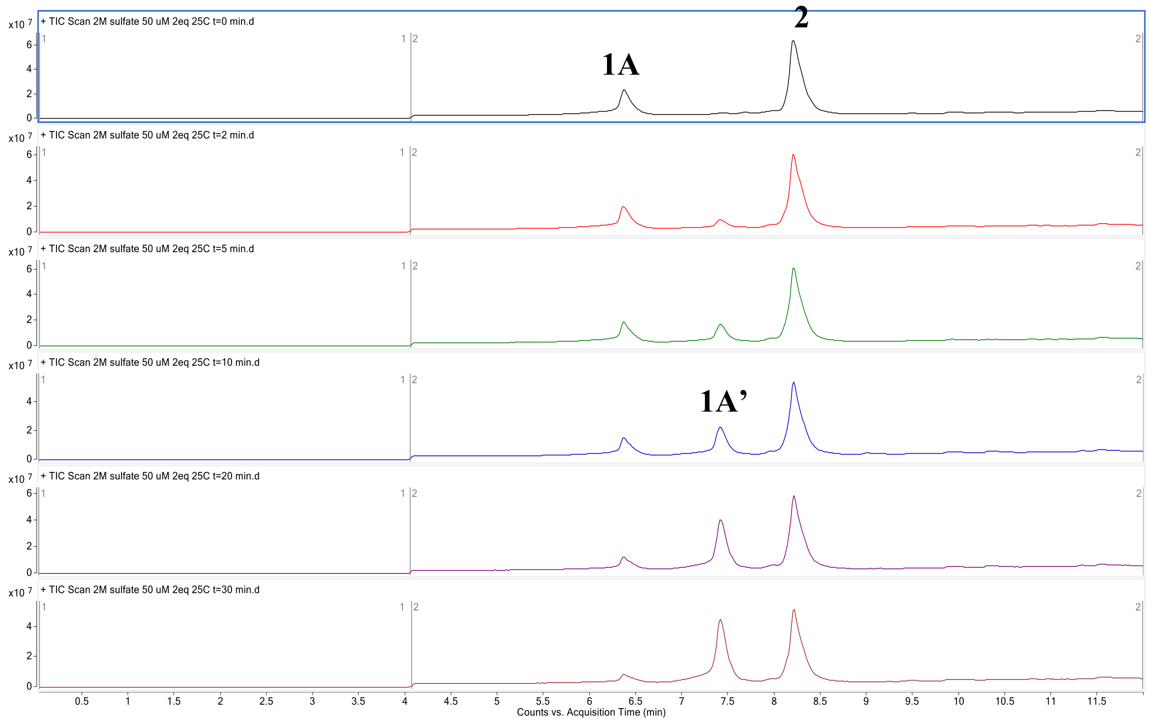

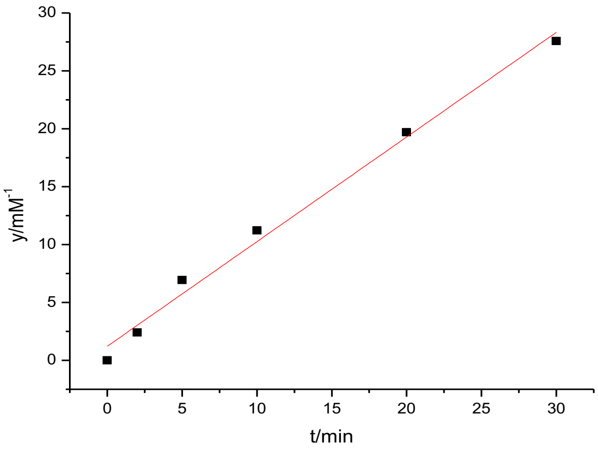

Figure S38. **Kinetics analysis for peptide 1A reacting with probe 2 with 2 M $(NH_4)_2SO_4$ at 24.4 °C.** Top, LC-MS chromatograms for the reaction between peptide **1A** and probe **2** at different time points (minutes). Reaction conditions: 0.05 mM **1A**, 0.1 mM probe **2**, 200 mM phosphate, 20 mM TCEP, 2 M $(NH_4)_2SO_4$, pH 8.0, 24.4 °C. Bottom, linear fitting of the kinetics data to second-order rate equation. Slope = 0.904, R^2 = 0.98. **1A'** refers to the product of the arylation reaction between **1A** and probe **2**.

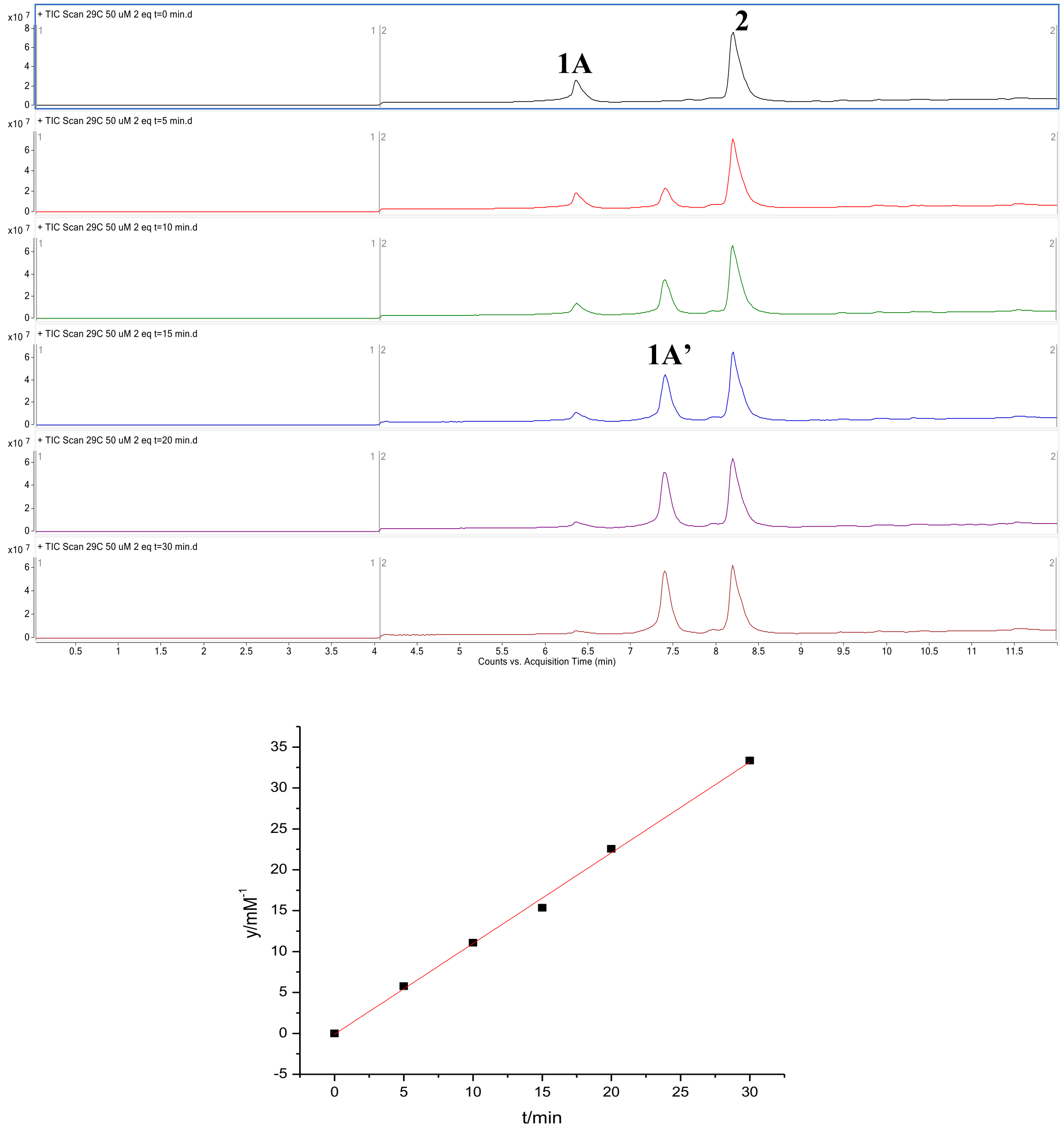

Figure S39. **Kinetics analysis for peptide 1A reacting with probe 2 with 2 M $(NH_4)_2SO_4$ at 28.1 °C.** Top, LC-MS chromatograms for the reaction between peptide **1A** and probe **2** at different time points (minutes). Reaction conditions: 0.05 mM **1A**, 0.1 mM probe **2**, 200 mM phosphate, 20 mM TCEP, 2 M $(NH_4)_2SO_4$, pH 8.0, 28.1 °C. Bottom, linear fitting of the kinetics data to second-order rate equation. Slope = 1.11, R^2 = 0.997. **1A'** refers to the product of the arylation reaction between **1A** and probe **2**.

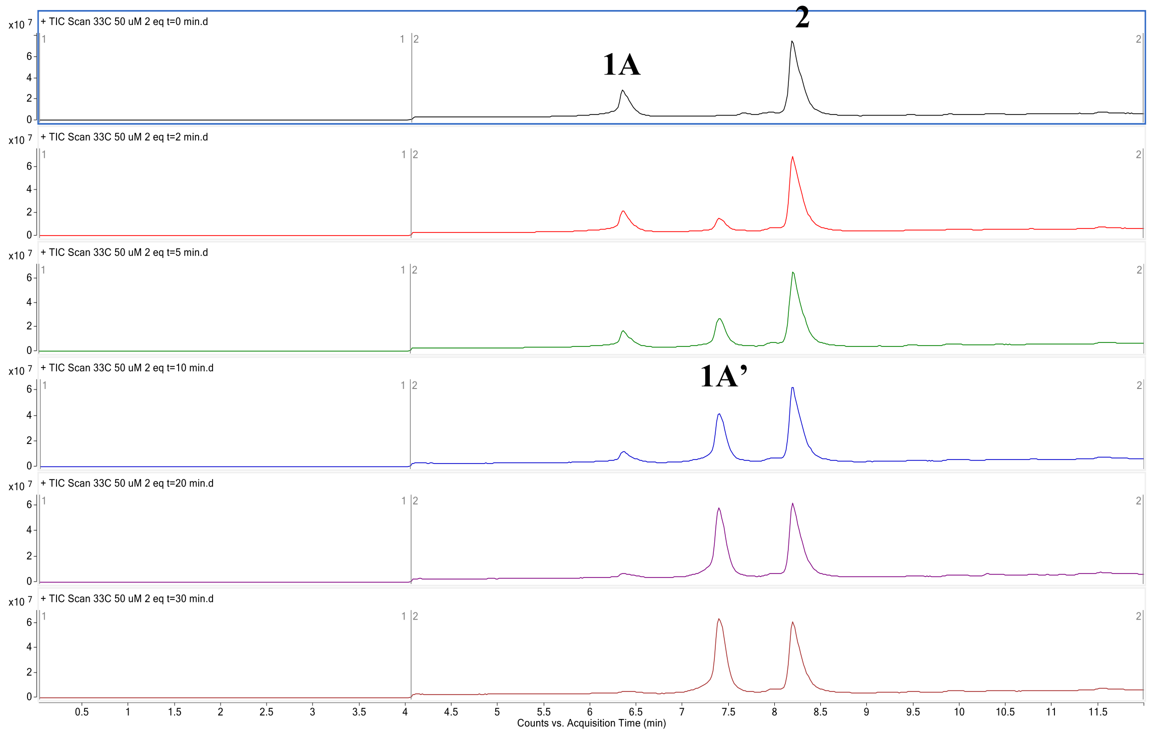

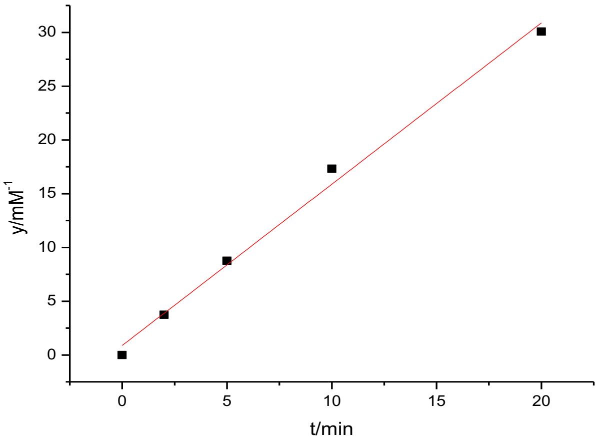

Figure S40. **Kinetics analysis for peptide 1A reacting with probe 2 with 2 M $(NH_4)_2SO_4$ at 33.3 °C.** Top, LC-MS chromatograms for the reaction between peptide **1A** and probe **2** at different time points (minutes). Reaction conditions: 0.05 mM **1A**, 0.1 mM probe **2**, 200 mM phosphate, 20 mM TCEP, 2 M $(NH_4)_2SO_4$, pH 8.0, 33.3 °C. Bottom, linear fitting of the kinetics data to second-order rate equation. Slope = 1.5, R^2 = 0.992. **1A'** refers to the product of the arylation reaction between **1A** and probe **2**.

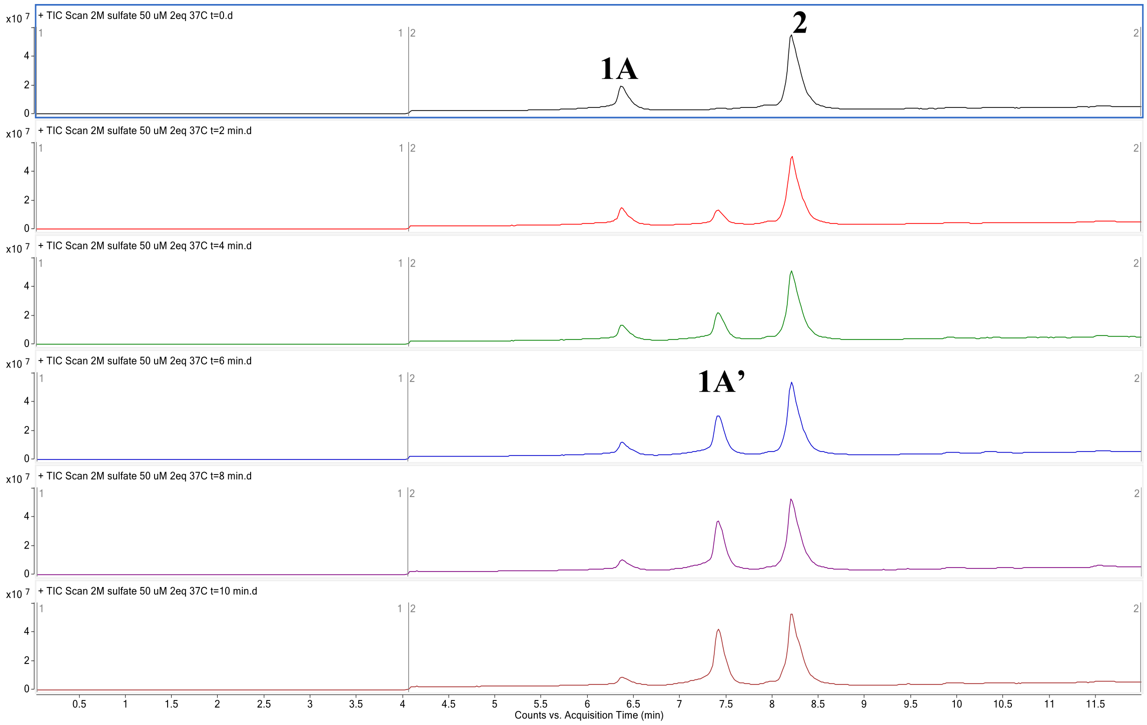

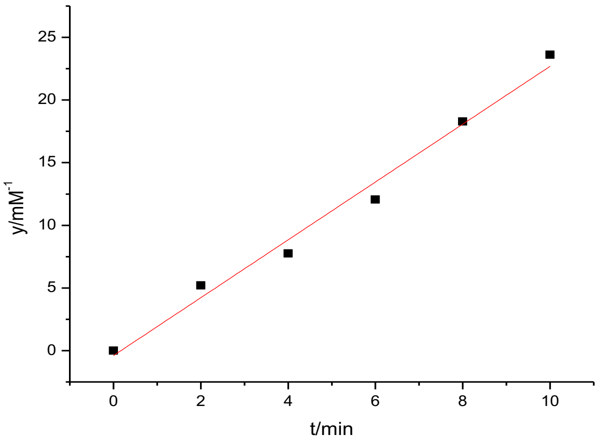

Figure S41. **Kinetics analysis for peptide 1A reacting with probe 2 with 2 M $(NH_4)_2SO_4$ at 37.5 °C.** Top, LC-MS chromatograms for the reaction between peptide **1A** and probe **2** at different time points (minutes). Reaction conditions: 0.05 mM **1A**, 0.1 mM probe **2**, 200 mM phosphate, 20 mM TCEP, 2 M $(NH_4)_2SO_4$, pH 8.0, 37.5 °C. Bottom, linear fitting of the kinetics data to second-order rate equation. Slope = 2.31, R^2 = 0.98. **1A'** refers to the product of the arylation reaction between **1A** and probe **2**.

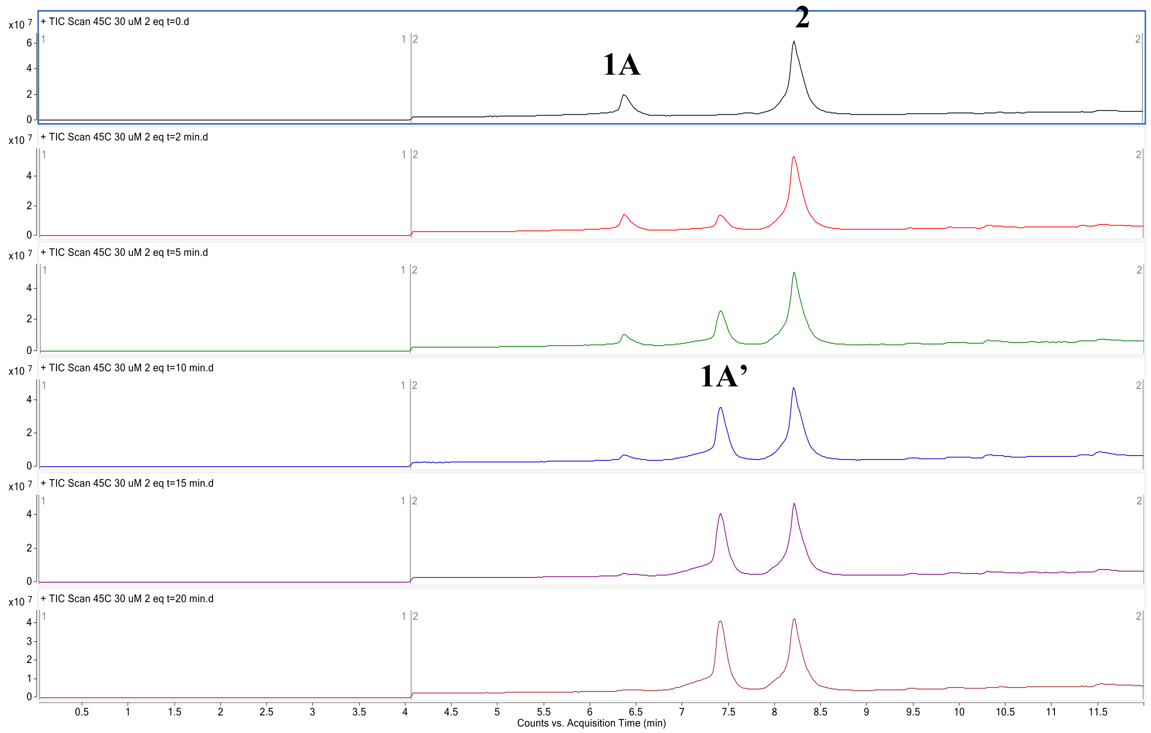

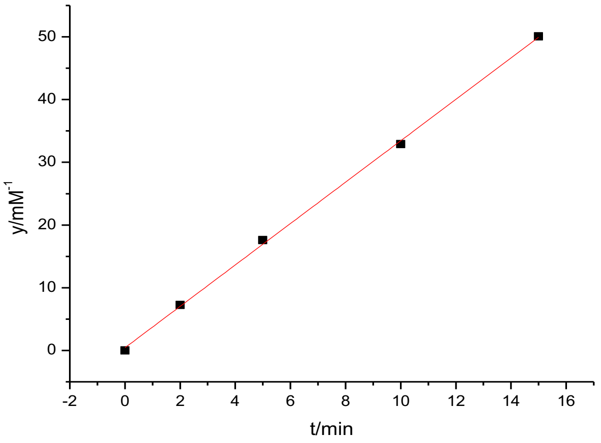

Figure S42. **Kinetics analysis for peptide 1A reacting with probe 2 with 2 M $(NH_4)_2SO_4$ at 44.8 °C.** Top, LC-MS chromatograms for the reaction between peptide **1A** and probe **2** at different time points (minutes). Reaction conditions: 0.03 mM **1A**, 0.06 mM probe **2**, 200 mM phosphate, 20 mM TCEP, 2 M $(NH_4)_2SO_4$, pH 8.0, 44.8 °C. Bottom, linear fitting of the kinetics data to second-order rate equation. Slope = 3.3, R^2 = 0.9992. **1A'** refers to the product of the arylation reaction between **1A** and probe **2**.

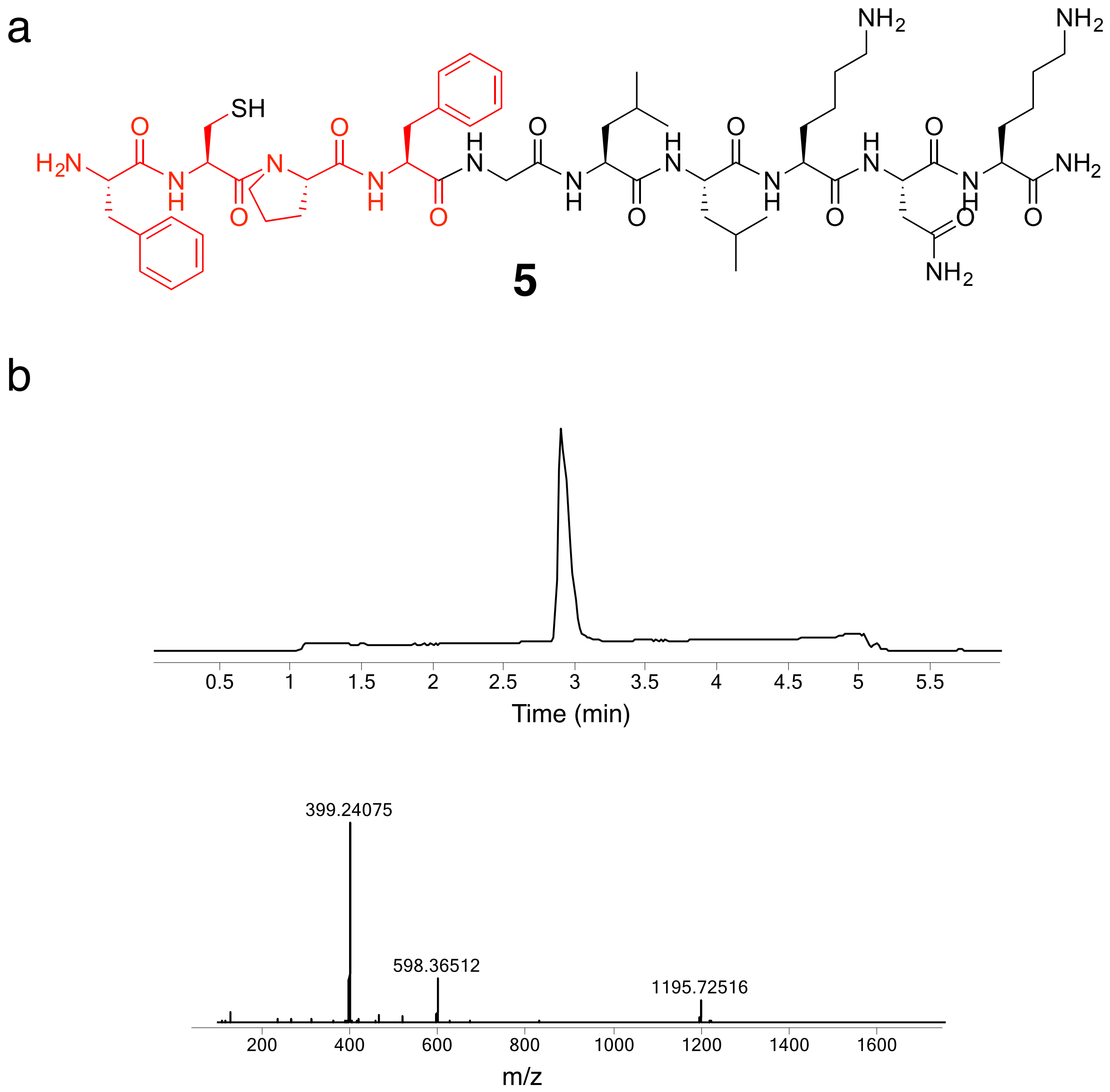

Figure S43. a) Chemical structure of peptide **5**. The part highlighted in red is universally 13C, 15N isotopic labeled. b) LC-MS chromatograms and the mass spectrum of the purified peptide. Calculated m/z for $[M+H]^+$: 1195.7, found 1195.7.

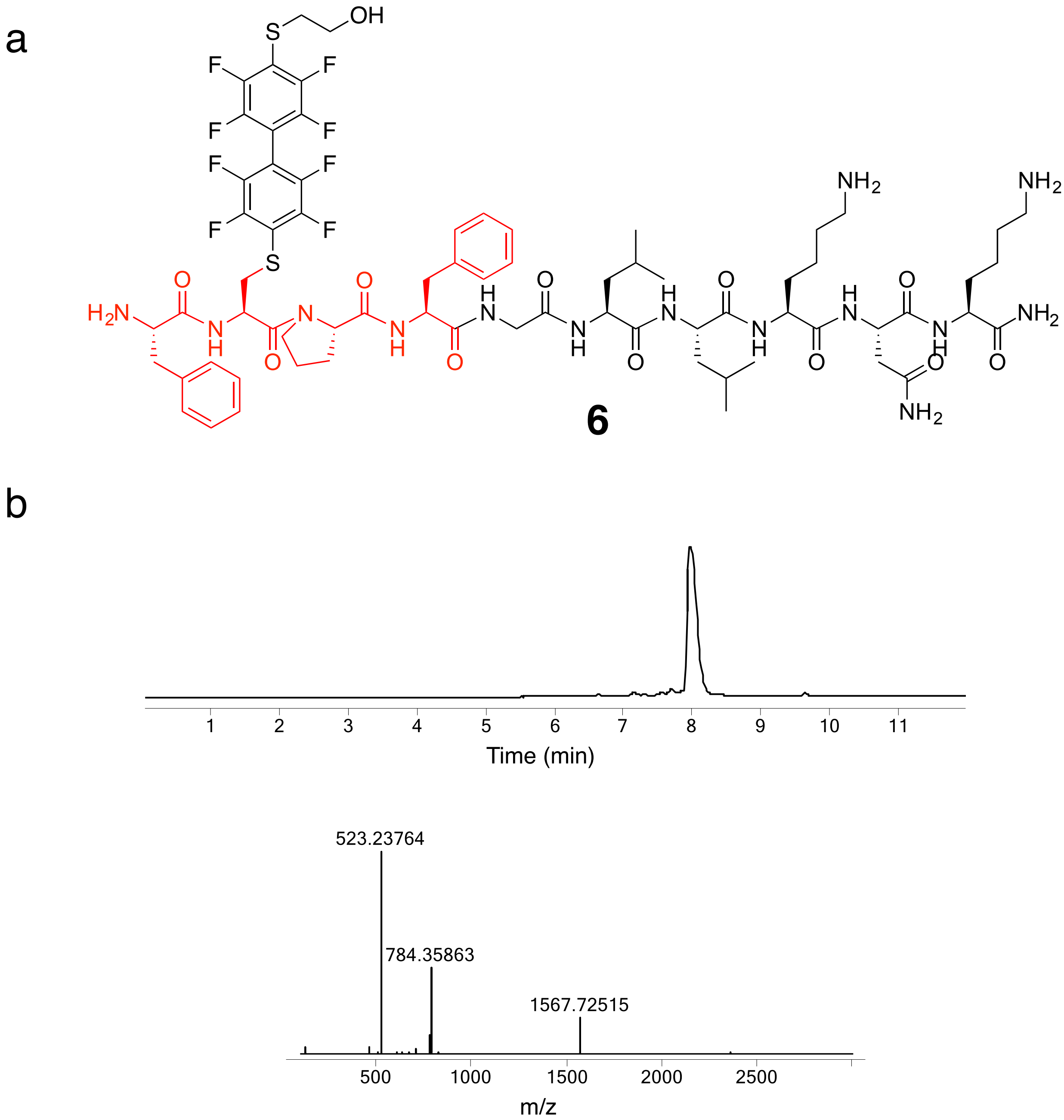

Figure S44. a) Chemical structure of peptide **6**. The part highlighted in red is universally 13C, 15N isotopic labeled. b) LC-MS chromatograms and the mass spectrum of the purified peptide. Calculated m/z for $[M+H]^+$: 1567.7, found 1567.7.

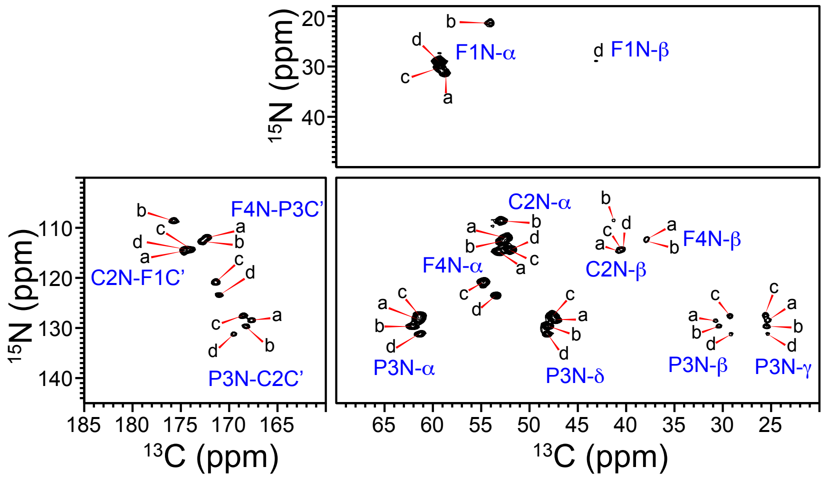

Figure S45. 2D ^{15}N-^{13}C correlation spectra of PFA-tagged peptide **6** show four distinct species. The spectra were acquired at 298 K and 16.5 kHz MAS, with a mixing time of 1.1 ms.

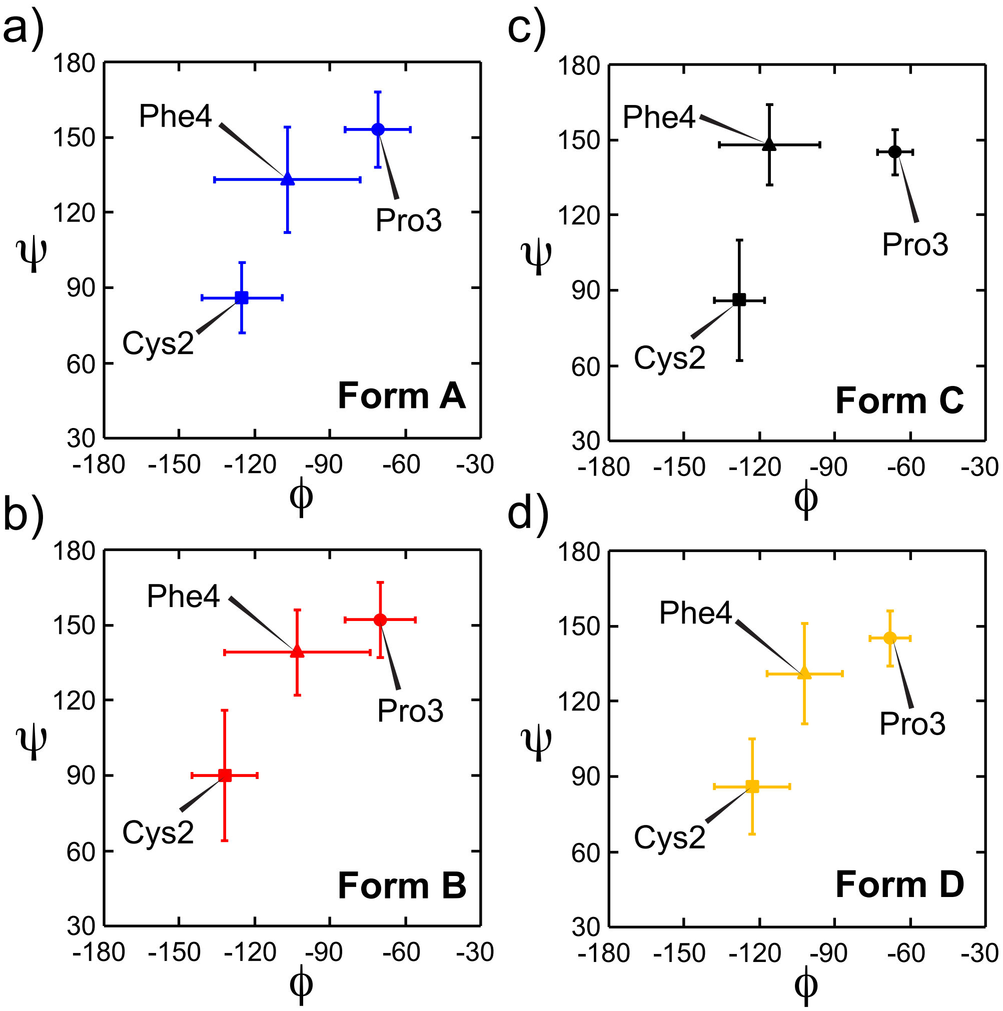

Figure S46. TALOSN prediction yields similar phi (ϕ) and psi (ψ) backbone torsion angles of the four forms of PFA-tagged peptide **6** based on the experimental chemical shifts. The predicted torsion angles of the four conformations of peptide **6** are shown in (a-d). The error bars in each plot are +/- one standard deviation, as given by the TALOSN output.

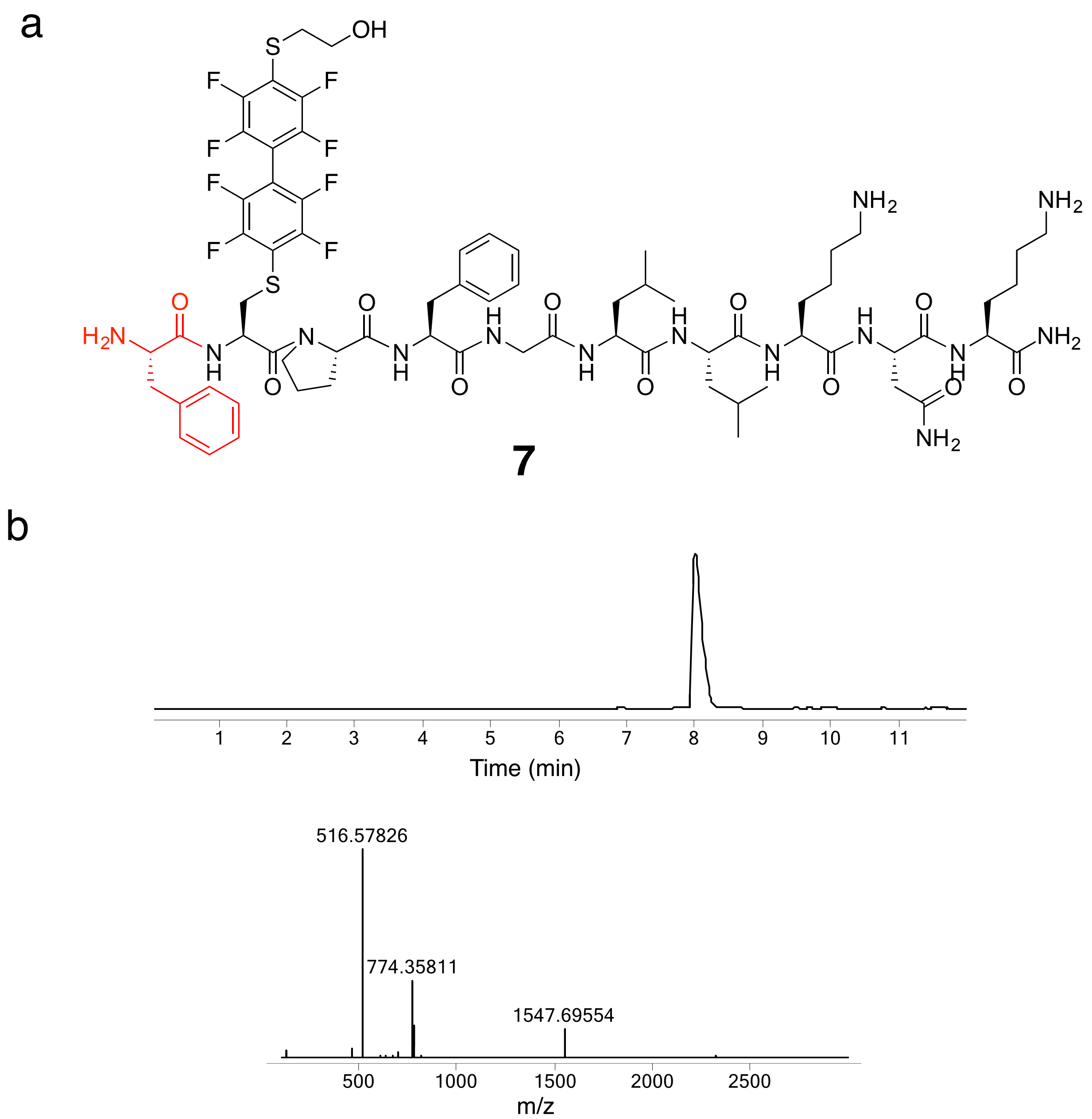

Figure S47. a) Chemical structure of peptide **7**. The part highlighted in red is universally 13C, 15N isotopic labeled. b) LC-MS chromatograms and the mass spectrum of the purified peptide. Calculated m/z for $[M+H]^+$: 1547.7, found 1547.7.

a

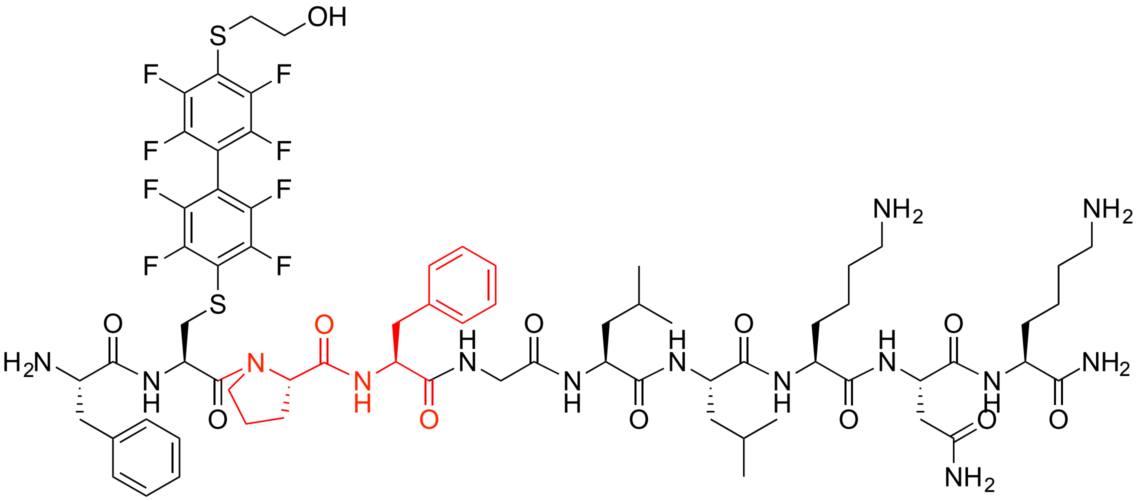

8

b

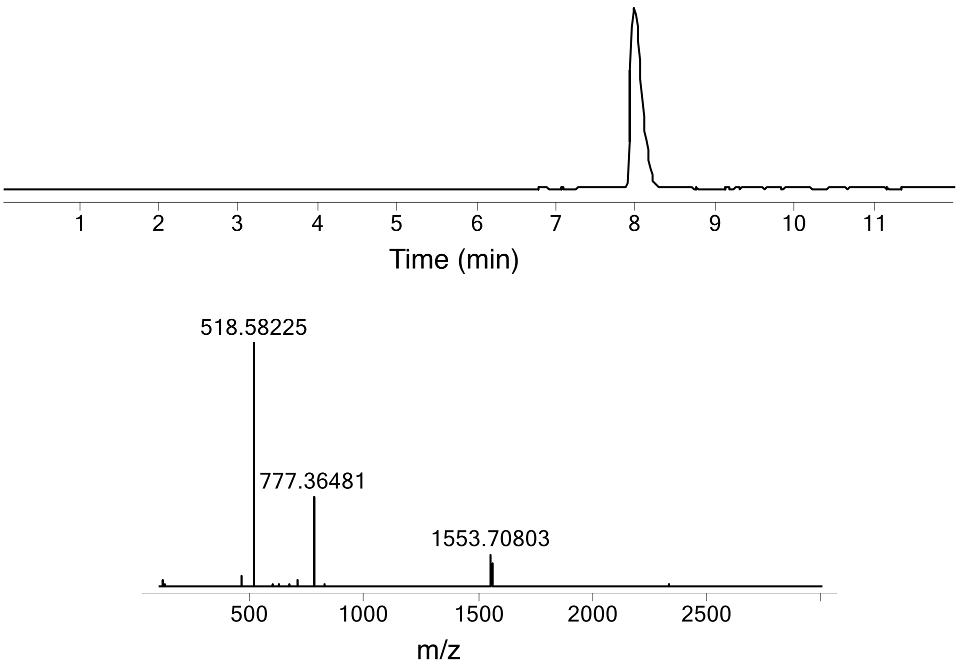

Figure S48. a) Chemical structure of peptide **8**. The part highlighted in red is universally 13C, 15N isotopic labeled. b) LC-MS chromatograms and the mass spectrum of the purified peptide. Calculated m/z for $[M+H]^+$: 1553.7, found 1553.7.

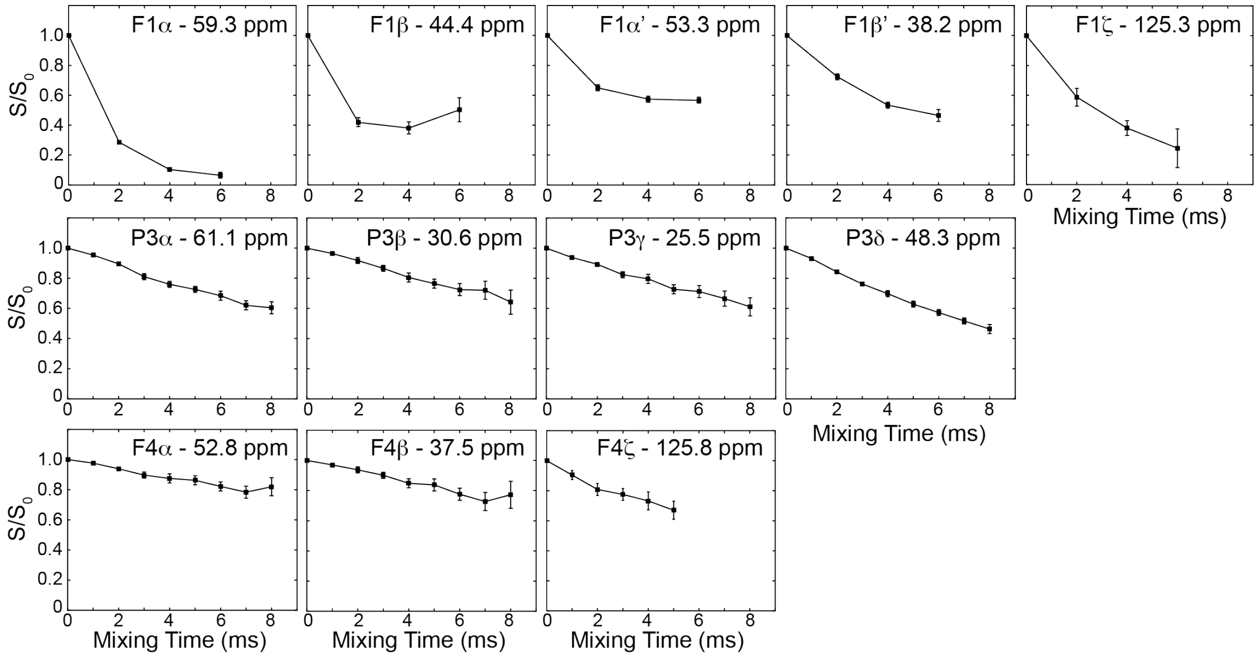

Figure S49. Experimental REDOR S/S$_0$ values as a function of mixing time for Phe1 (top), Pro3 (middle) and Phe4 (bottom). Phe1 was labeled in one peptide, and Pro3 and Phe4 were labeled in a second peptide. Note that two forms of Phe1 Cα and Cβ are resolved from each other, denoted with or without a prime. The Phe1 peaks show interesting dephasing behavior, most likely due to the complex nature of the system. The possibility of incomplete inversion of the ^{19}F spectrum is less likely, since the ^{19}F inversion pulses in the REDOR pulse train have field strengths of 71 kHz, compared to the ^{19}F chemical shift anisotropy of 130-150 ppm or 49-56 kHz at the temperature of the experiment. We also utilize composite 90°-180°-90° ^{19}F inversion pulses to compensate for B$_1$ field inhomogeneity.

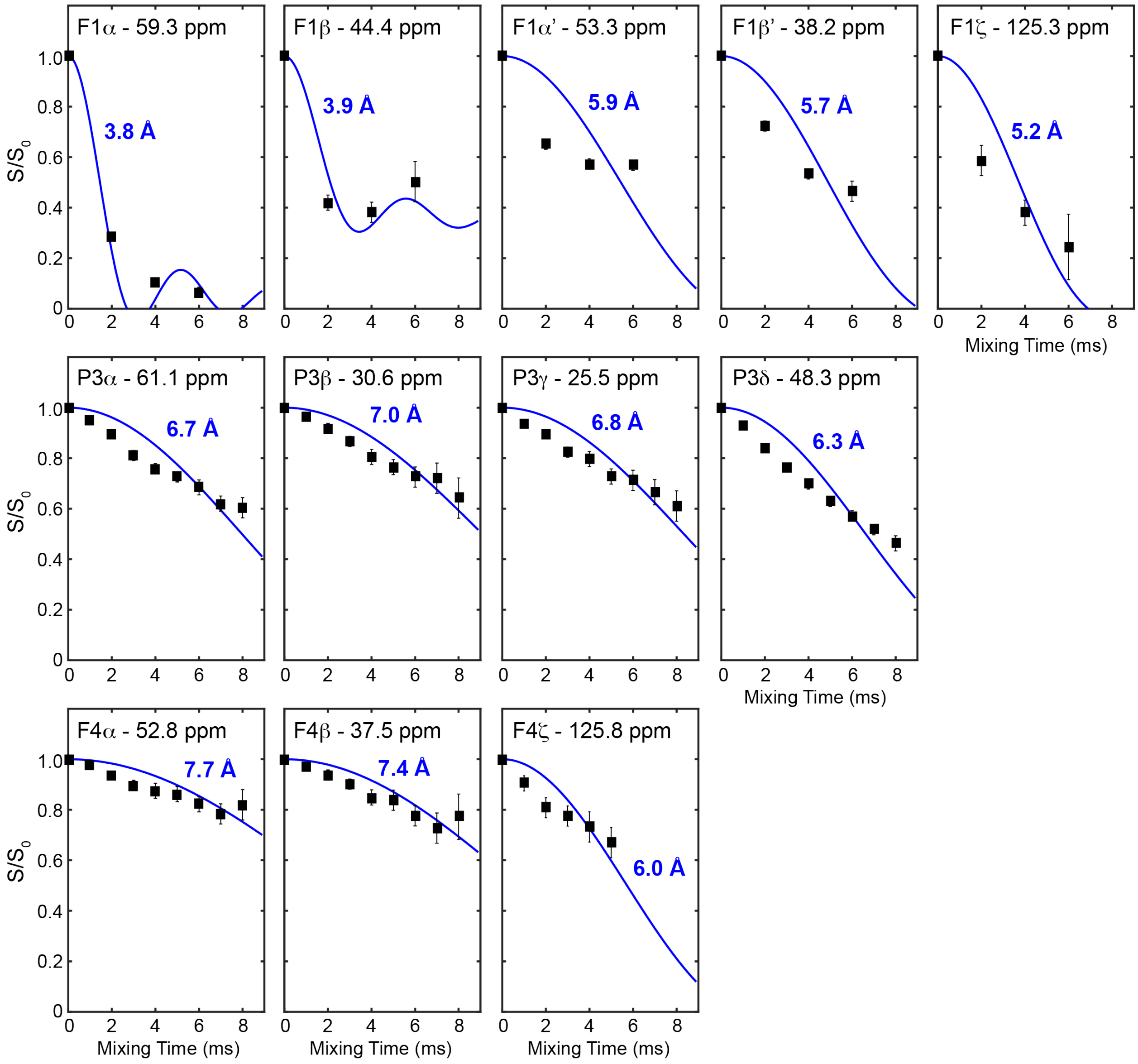

Figure S50. 2-spin REDOR simulations (blue) overlaid on the experimental S/S_0 data for Phe1 (top), Pro3 (middle) and Phe4 (bottom). Note that the simulated curve for F1β has been scaled by 0.5 to fit the data.

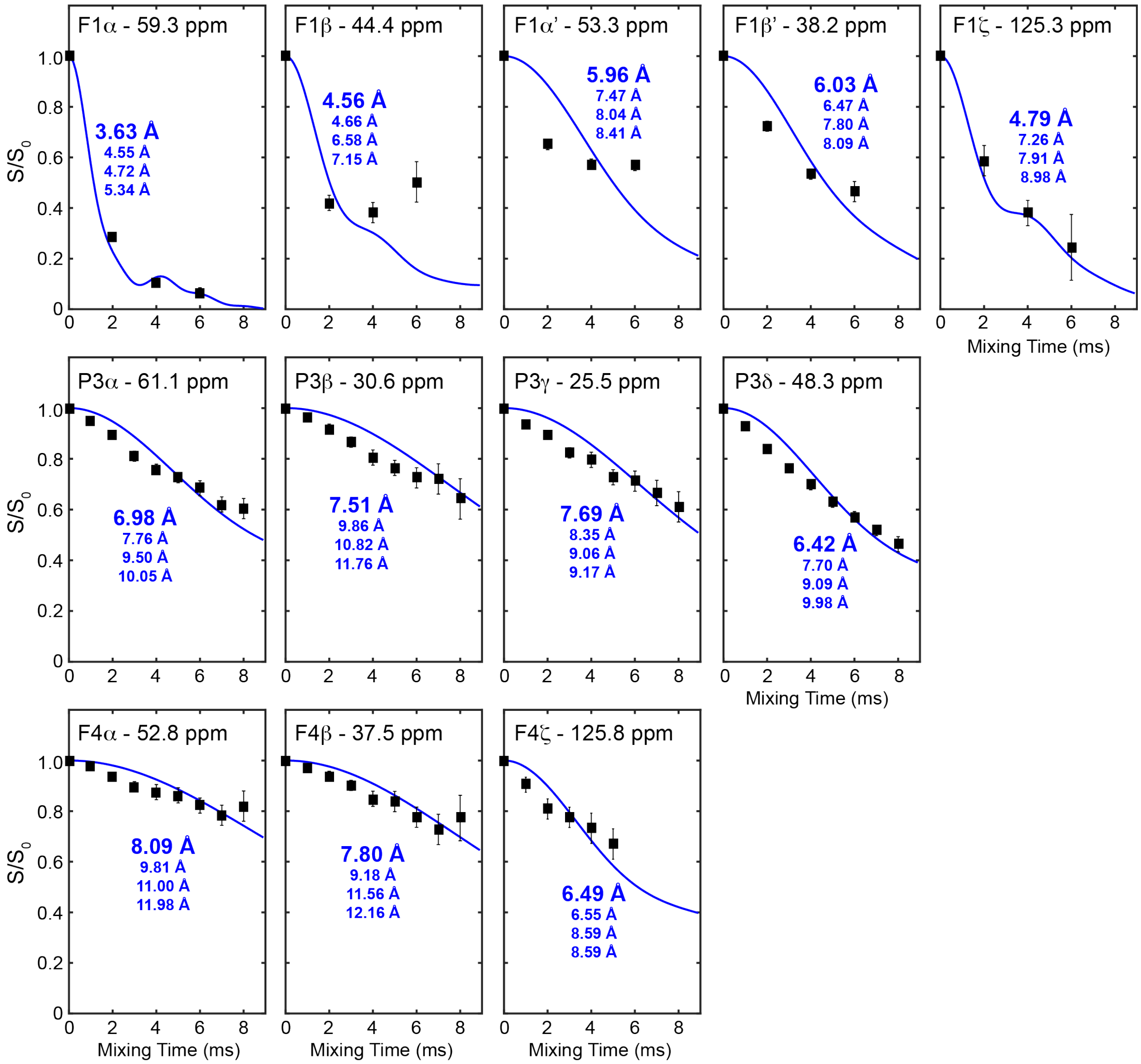

Figure S51. Best fit 5-spin model-dependent REDOR curves. The four shortest ^{13}C-^{19}F distances are listed in each panel.

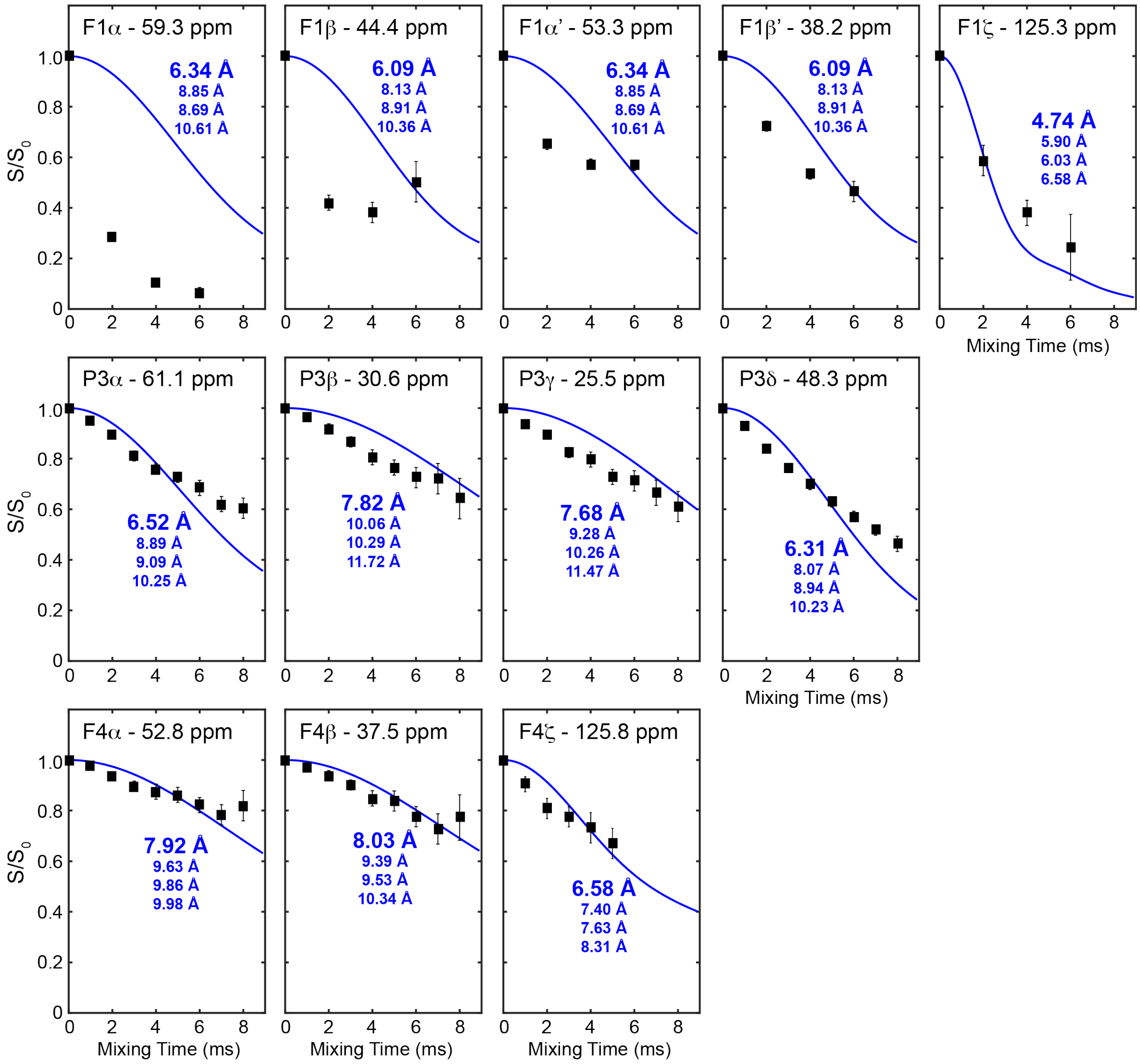

Figure S52. 5-spin REDOR simulations using dihedral angles of ψ = -60° and χ_1 = 150° angle of Phe1, χ_1 = -120°, χ_2 = 180° and χ_3 = 180° of Cys2 and χ_1 = -120° of Phe4. The four shortest ^{13}C-^{19}F distances are listed in each panel.

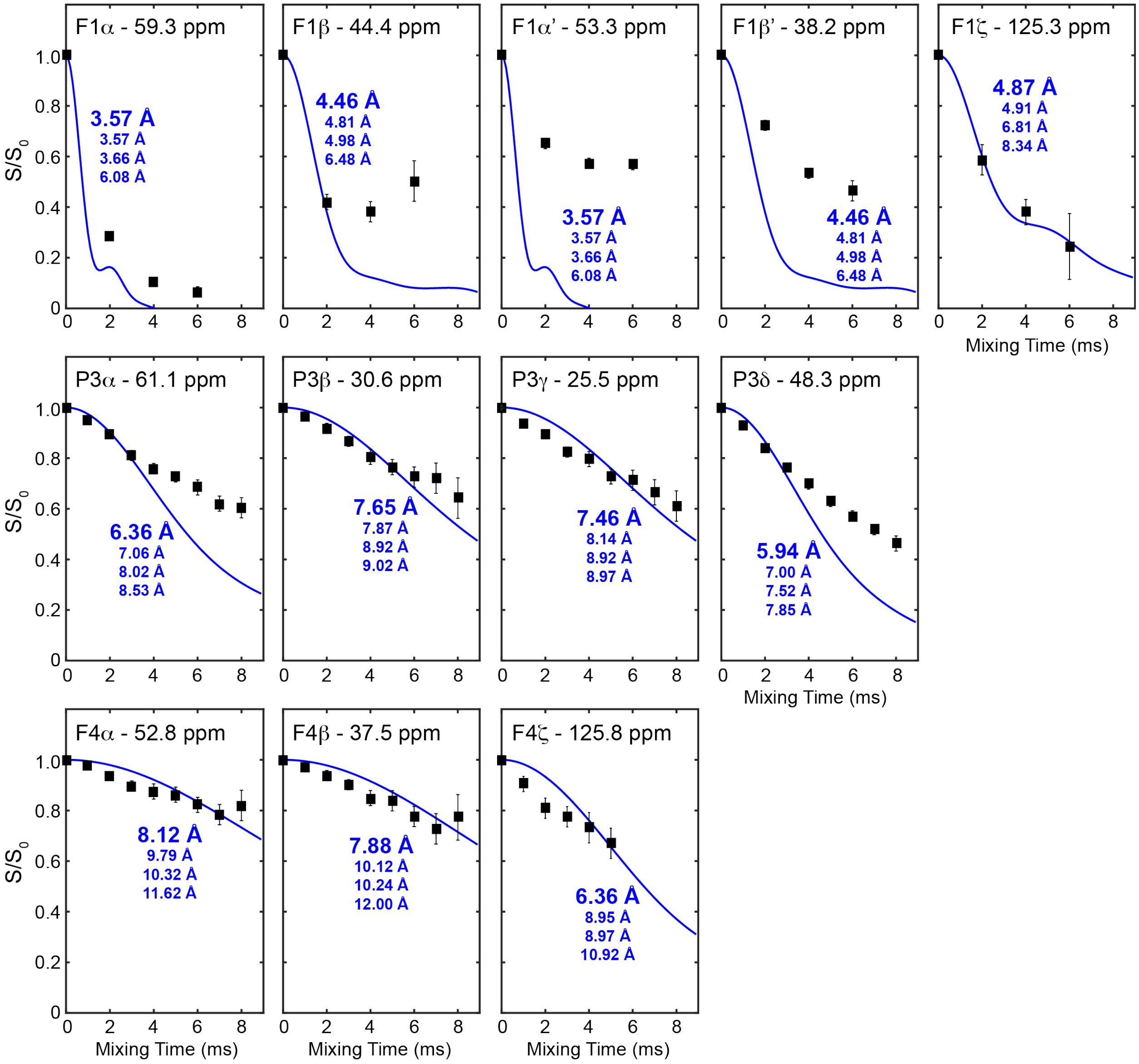

Figure S53. 5-spin REDOR simulations using dihedral angles of ψ = 90° and χ_1 = -60° angle of Phe1, χ_1 = 60°, χ_2 = -60° and χ_3 = -30° of Cys2 and χ_1 = -90° of Phe4. The four shortest ^{13}C-^{19}F distances are listed in each panel.

a) Calculated structure A   Torsion angles

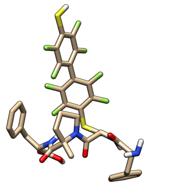

	ϕ	ψ	χ_1	χ_2	χ_3
**F1**	N/A	***+168***	-166	+63	N/A
**C2**	***-53***	***+119***	+176	+49	-140
**P3**	***-70***	***-20***	N/A	N/A	N/A
**F4**	***-125***	N/A	-40	-63	N/A

5-spin REDOR simulations

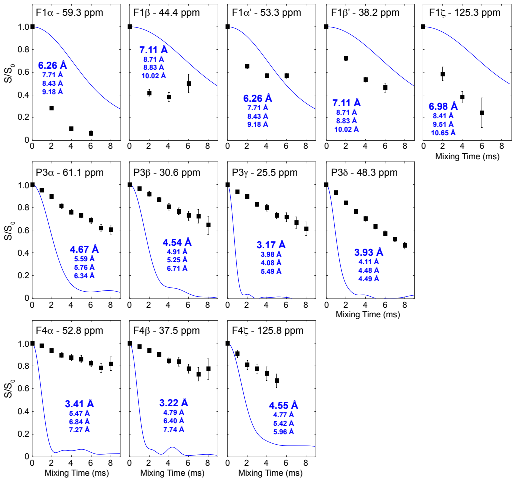

b) Calculated structure B   Torsion angles

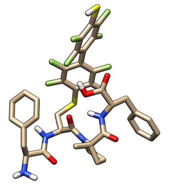

	ϕ	ψ	χ_1	χ_2	χ_3
**F1**	N/A	***+158***	-171	-114	N/A
**C2**	***-64***	***+102***	+172	-99	+143
**P3**	***-83***	***+39***	N/A	N/A	N/A
**F4**	***-135***	N/A	-52	-86	N/A

5-spin REDOR simulations

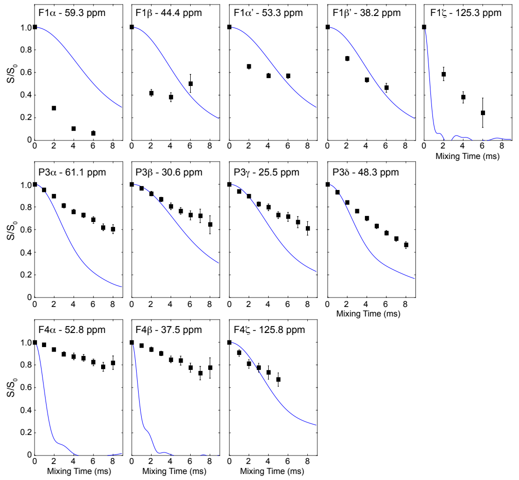

c) Calculated structure C   Torsion angles

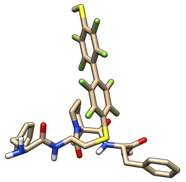

	ϕ	ψ	χ_1	χ_2	χ_3
**F1**	N/A	***+125***	-180	-95	N/A
**C2**	***-135***	***+114***	+175	+50	+50
**P3**	***-76***	***+63***	N/A	N/A	N/A
**F4**	***-61***	N/A	-172	+88	N/A

5-spin REDOR simulations

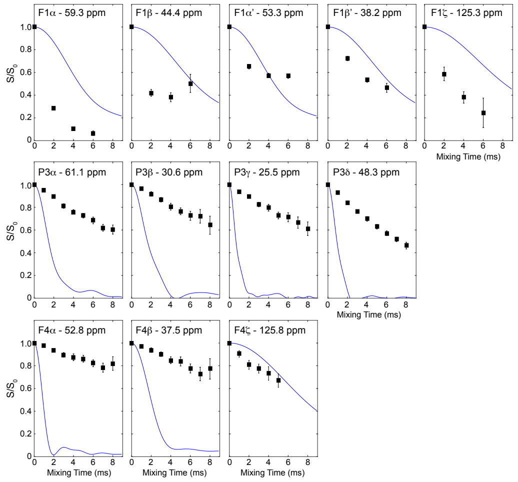

d) NMR structure model 2 Torsion angles

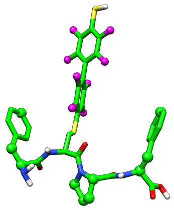

	ϕ	ψ	χ_1	χ_2	χ_3
**F1**	N/A	***-60***	+150	-180	N/A
**C2**	***-125***	***+86***	-120	-180	-180
**P3**	***-73***	***+153***	N/A	N/A	N/A
**F4**	***-106***	N/A	-120	-180	N/A

5-spin REDOR simulations

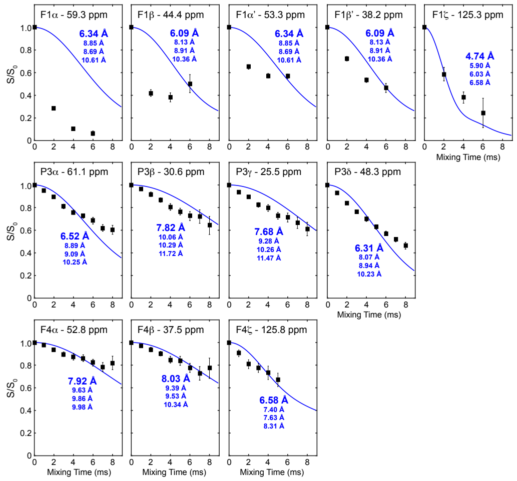

Figure S54. Comparison of the structural models from ssNMR study and calculation.

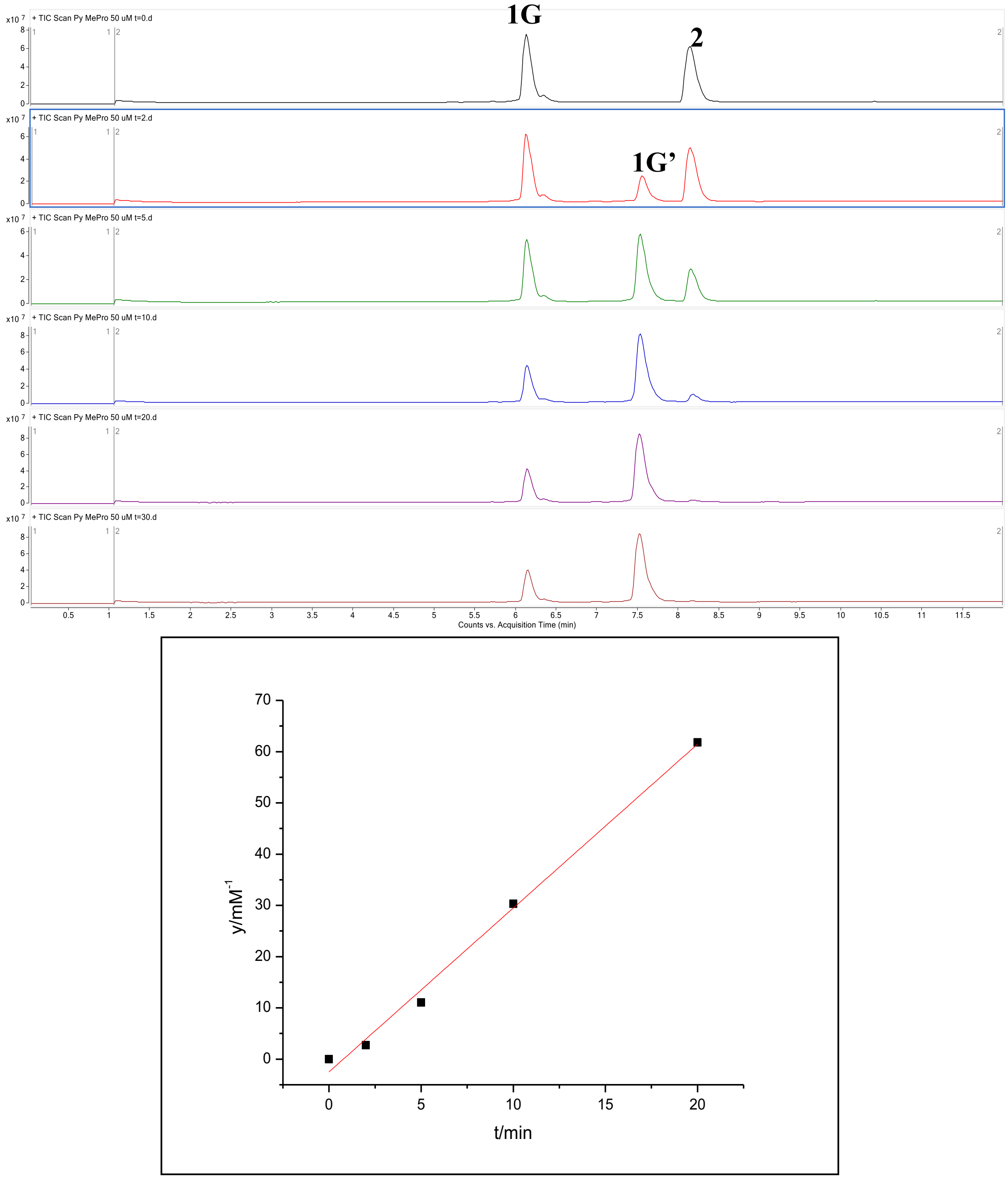

Figure S55. **Kinetics analysis for peptide 1G reacting with probe.** Top, LC-MS chromatograms for the reaction between peptide **1G** and probe **2** at different time points (minutes). Reaction conditions: 0.05 mM **1G**, 0.1 mM probe **2**, 200 mM phosphate, 20 mM TCE, pH 8.0, 37 °C. Bottom, linear fitting of the kinetics data to second-order rate equation. Slope = 3.2, R^2 = 0.992. **1G'** refers to the product of the arylation reaction between **1G** and probe **2**.

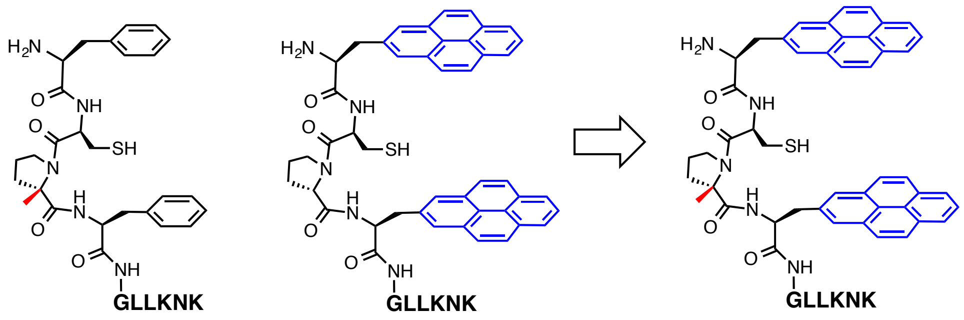

1B 1F 1G

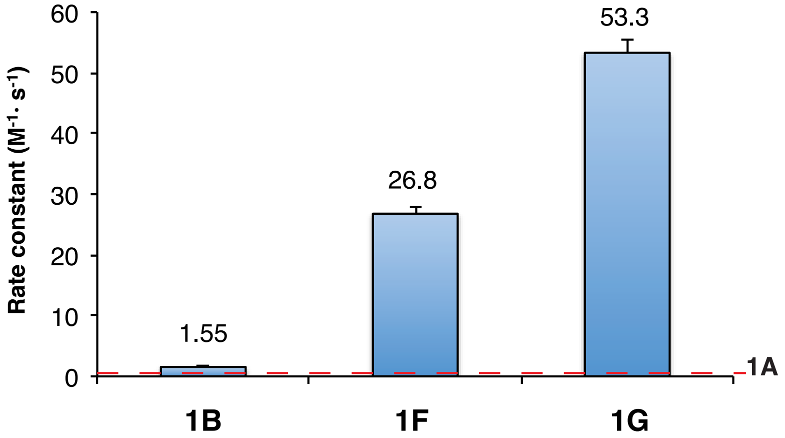

Figure S56. Convergent effect of α–methylproline and hydrophobic side chain further enhances the reactivity. The rate constants for peptide **1B**, **1F** and **1G** reacting with probe **2**. The red dashed line indicates the rate constant for reaction involving **1A**. The π-clamp mutant **1G** shows a rate constant 85-fold higher than π-clamp peptide **1A**.
